# Baseline depressive symptoms, personal control, and concern moderate the effects of preoperative psychological interventions: the randomized controlled PSY-HEART trial

**DOI:** 10.1007/s10865-022-00319-0

**Published:** 2022-05-06

**Authors:** Nicole Horn, Johannes A. C. Laferton, Meike C. Shedden-Mora, Rainer Moosdorf, Winfried Rief, Stefan Salzmann

**Affiliations:** 1 Department of Clinical Psychology and Psychotherapy, Philipps University of Marburg, Gutenbergstraße 18, 35032 Marburg, Germany; 2 Division of Medical Psychology, Department of Medicine, Health and Medical University Potsdam, Potsdam, Germany; 3 Department of Psychology, Medical School Hamburg, Hamburg, Germany; 4 Department of Psychosomatic Medicine and Psychotherapy, University Medical Center Hamburg-Eppendorf, Hamburg, Germany; 5 Department for Cardiovascular Surgery, Heart Center, Philipps University of Marburg, Marburg, Germany

**Keywords:** Expectation, Depression, Personal control, Concern, Placebo, Preoperative psychological intervention, CABG, PSY-HEART

## Abstract

This study examined whether baseline (3–14 days pre-surgery) levels of (i) depressive or (ii) anxiety symptoms and (iii) illness beliefs moderate the effects of additional preoperative interventions before coronary artery bypass graft surgery on (i) depressive or (ii) anxiety symptoms and (iii) illness beliefs 1 day before surgery, 1 week and 6 months after surgery. In the PSY-HEART trial, 115 patients were assessed. They were randomized into one of three groups: 1. receiving standard medical care only (SMC), additional psychological interventions: 2. aiming to optimize patients’ expectations (EXPECT), or 3. focusing on emotional support. Patients with a higher baseline level of depressive symptoms receiving a preoperative psychological intervention indicated lower depressive symptoms 6 months after surgery compared to SMC. EXPECT increased personal control and concern levels in patients with low baseline personal control/concern 1 day before surgery. Brief preoperative psychological interventions can improve psychological outcomes in heart surgery patients. Baseline status may moderate these effects. The study has been approved by the medical ethics committee of the Philipps University of Marburg and has been pre-registered at www.clinicaltrials.gov (NCT01407055) on August 1, 2011.

**Supplementary Information:**

The online version contains supplementary material available at 10.1007/s10865-022-00319-0.

## Introduction

Cardiovascular diseases are associated with a high risk of mortality, disability, a decreased quality of life, and increased costs for the healthcare system (Murray & Lopez, 2013; Virani et al., 2020). Coronary artery bypass grafting (CABG) is an established treatment option for patients with advanced coronary artery disease that has been thoroughly studied over several decades (Hawkes et al., 2006). However, it is still unknown why a substantial number of patients faces problems in the recovery process and does not benefit as much from the surgery as surgeons would predict (Blumenthal et al., 2003; Burg et al., 2003; Hawkes & Mortensen, 2006; Hawkes et al., 2006; Salzmann, Euteneuer, et al., 2020; Salzmann, Salzmann-Djufri, et al., 2020). Patients’ recovery after surgery is not explained by medical factors alone; recovery seems to be a multidimensional phenomenon in which physical, psychological, and social factors play important roles as well (Auer et al., 2016; Hawkes et al., 2006; Sadeghi et al., 2017). Growing evidence suggests the importance of psychological preparation for improving post-surgery physical outcomes and psychological outcomes (i.e., quality of life, disability, pain, morbidity, length of hospital stay) (Auer et al., 2017; Levett & Grimmett, 2019; Salzmann, Euteneuer, et al., 2020; Salzmann, Salzmann-Djufri, et al., 2020; Wynter-Blyth & Moorthy, 2017). A better understanding of whether and when psychological interventions affect specific outcomes may help design even more powerful interventions and make better predictions of which patients will benefit from which psychological intervention. More specifically, in this article, we assessed whether baseline depressive symptoms moderated the intervention effects on depressive symptoms, whether baseline anxiety levels moderated the intervention effects on anxiety levels, and whether baseline illness beliefs moderated the intervention effects on illness beliefs (i.e., whether baseline control beliefs moderated the intervention effects on control beliefs).

The Common Sense Model (CSM) aims to explain how people react to a perceived threat. It describes that patients have illness beliefs or perceptions about the experience of their illness. According to the CSM, patients experience surgery as a threat, triggering cognitive and emotional processes (Leventhal & Cameron, 1987; Leventhal et al., 1992). These processes affect illness behavior by triggering strategies to cope with the threat. The cognitive response to a health threat consists of a person's subjective illness beliefs and expectations about the identity (symptoms), the timeline (how long it will continue), the consequences (results of the symptoms), and the perceived controllability (personal and treatment control—possibility of recovery through my acting or medical treatment) of an illness (Kidd et al., 2016; Leventhal et al., 2001).

Illness beliefs have been consistently shown to be related to short-term and long-term heart surgery outcomes, the recovery process, and behavioral change and therefore may help to explain why surgery is more effective for some patients than for other patients (Broadbent et al., 2009; Juergens et al., 2010; Parfeni et al., 2013; Petrie et al., 1996, 2002; Poole et al., 2015; Salzmann, Euteneuer, et al., 2020; Salzmann, Salzmann-Djufri, et al., 2020; Weinman et al., 2000). In particular, personal control describes a person’s belief that he/she is confident to execute a specific behavior and that this behavior will affect one’s health (Laferton et al., 2017). Personal control is a behavior outcome expectation that describes how much patients are convinced that they can recover from or control the disease by their own action (Lau & Hartman, 1983; Leventhal et al., 2001). An association was found between higher personal control beliefs and better quality of life/well-being, lower depression and anxiety in CABG patients (Broadbent et al., 2015; Gallagher & McKinley, 2009; Kidd et al., 2016; Petrie et al., 2002, 2012). Changing illness beliefs has enhanced health outcomes in several studies with cardiac patients (Davies et al., 2008; Juergens et al., 2010; Keogh et al., 2011; Petrie et al., 2002, 2012). Research may benefit from focusing more on patient beliefs and expectations, especially about personal control, in exploring the recovery process. Higher scores of preoperative perceived control have been shown to predict postoperative quality of life and lower levels of depression in CABG patients (Kidd et al., 2016). Nonetheless, little is known about the question of which preoperative psychological intervention can influence what kind of illness beliefs and who will benefit from such an intervention specifically (Kidd et al., 2016).

Besides the cognitive responses to a perceived health threat, the CSM highlights the importance of emotional factors in coping with a disease, e.g., illness beliefs such as concern or emotions (Leventhal & Cameron, 1987; Leventhal et al., 1992). Other emotional factors such as depression or anxiety are also highly relevant in cardiac surgery patients: Depression is highly prevalent in patients undergoing CABG (Blumenthal et al., 2003; Head et al., 2013; Poole et al., 2014, 2017; Tully et al., 2009; Young et al., 2019). 20–40% of CABG surgery patients are affected by depression (Blumenthal et al., 2003; Connerney et al., 2001; Young et al., 2019). Depressed patients undergoing CABG surgery report a lower health-related quality of life, have a higher postoperative rate of depression, a higher risk of rehospitalization and death, and stay longer in hospital after the surgical procedure independent from medical factors compared to non-depressed patients (Auer et al., 2017; Blumenthal et al., 2003; Connerney et al., 2001; Contrada et al., 2004; Mallik et al., 2005; Morone et al., 2010; Oxlad et al., 2006; Rollman et al., 2009; Rumsfeld et al., 2004; Timberlake et al., 1997). Dunkel et al. (2011) suggest that patients with higher depression levels might benefit most from additional psychological intervention. Similarly, preoperative anxiety seems to be associated with a negative postoperative course, yet fewer is known about this relationship, especially in CABG patients (Arthur et al., 2000; Heilmann et al., 2016; Lamarche et al., 1998; Székely et al., 2007). Since preoperative anxiety and depressive symptoms seem to be important predictors of the postoperative recovery process, psychological interventions targeting these symptoms could improve postoperative physical and psychological outcomes (i.e., such as depression or anxiety). Heilmann et al. (2016) reported that a preoperative intervention reduced the preoperative and postoperative state anxiety compared to a control group. However, it is mainly unknown how specific interventions can be tailored to the individual needs of CABG patients to reduce patients anxiety and depression levels.

The PSY-HEART trial indicated that receiving a preoperative psychological intervention aiming to optimize patients’ expectations (EXPECT) led to reduced illness-related disability as the primary outcome (Rief et al., 2017). Several positive effects on secondary outcomes were also found: For instance, the EXPECT intervention indicated increased physical and mental quality of life 6 months after CABG surgery and fewer days of hospitalization in comparison to standard medical care (SMC) only (for further information, see Auer et al., 2017; Rief et al., 2017). For depression (another secondary outcome), a non-significant trend was found in favor of EXPECT and SUPPORT (an attention control group receiving the same amount of time and attention by the psychologist but without working specifically on expectations) compared to SMC only, when assessing baseline and follow-up scores 6 months after surgery. However, it is still unclear who benefitted the most from the preoperative psychological interventions in the PSY-HEART trial and when these interventions seemed to work regarding patients’ depressive and anxiety symptoms as well as illness-beliefs. A meta-analysis of Bower et al. (2013) indicated that patients who had higher levels of depression at baseline showed greater treatment effects than patients with lower levels of depression at baseline. For anxiety, baseline anxiety was the most frequently examined moderator of the effectiveness of psychological and psychoeducational interventions for anxiety in a meta-analysis (Moreno-Peral et al., 2020). Since patients’ depressive and anxiety symptoms and patients’ illness beliefs (i.e., perceived personal control or concern) are considered important outcome predictors, especially in heart surgery patients (Salzmann, Euteneuer, et al., 2020; Salzmann, Salzmann-Djufri, et al., 2020), a more thorough understanding of the (psychological) intervention effects over time regarding these psychological factors is crucial. To better understand how, when and for whom the preoperative psychological interventions (EXPECT: optimizing expectation group; SUPPORT: emotional support/attention control group) seemed to improve depressive and anxiety symptoms, and illness beliefs in the PSY-HEART trial (Rief et al., 2017), this secondary exploratory analysis examined whether (i) baseline scores of depressive symptoms moderated the effects of the preoperative psychological interventions on depressive symptoms in heart surgery patients 1 day before surgery, 4 to 6 days after surgery and 6 months after surgery, whether (ii) baseline anxiety symptom scores moderated the effects of the preoperative psychological interventions on anxiety symptoms in heart surgery patients 1 day before surgery, 4 to 6 days after surgery and 6 months after surgery, and whether (iii) baseline scores of the illness beliefs (i.e. perceived personal control or concern) moderated the effects of the preoperative psychological interventions on illness beliefs (i.e. perceived personal control or concern) in heart surgery patients 1 day before surgery, 4 to 6 days after surgery and 6 months after surgery.

## Methods

### Study design

The study is part of the randomized controlled clinical PSY-HEART trial (Rief et al., 2017) (see Laferton et al., 2013, for the study protocol). The PSY-HEART trial was approved by the medical ethics committee of the Philipps University of Marburg and was pre-registered at ClinicalTrials.gov (Identifier: NCT01407055). It examined the effects of preoperative psychological interventions on postoperative physical and psychological outcomes in heart surgery patients (CABG or CABG plus heart valve surgery). Participants were randomly assigned to one of three groups: (1) receiving the standard medical treatment (Standard Medical Care—SMC); (2) receiving SMC and additional psychological treatment focusing on optimizing patients’ expectations (EXPECT); (3) receiving SMC and additional psychological treatment providing attention and emotional support by a psychologist (SUPPORT). Patients were assessed at four measurement time points: 3–14 days pre-surgery (T0, baseline), 1 day pre-surgery [after the psychological intervention, but before surgery (T1)], 6–8 days (“1 week”) post-surgery (T2), approximately 6 months post-surgery (T3, follow-up).

The data collection took place from April 2011 to May 2015 in the Department of Cardiovascular Surgery, Philipps University of Marburg, Germany.

### Participants

Participants have been recruited from the waiting list of the Heart Surgery center. If a CABG surgery (with or without heart valve surgery) was planned, the patients were contacted before hospital admission. Interested patients were invited to a first appointment. Thereby they were informed and gave written informed consent.

Inclusion criteria were age between 18 and 80 years, speaking and understanding German fluently, being able to give informed consent. Exclusion criteria were a serious comorbid psychiatric or physical (non-cardiac) condition (e.g. acute psychosis, dementia) that hampers the participation at baseline or will do most likely within 6 months until study completion at follow-up. Further exclusion criteria were previous cardiac surgeries and participation in a different research program.

**Fig. 1:**
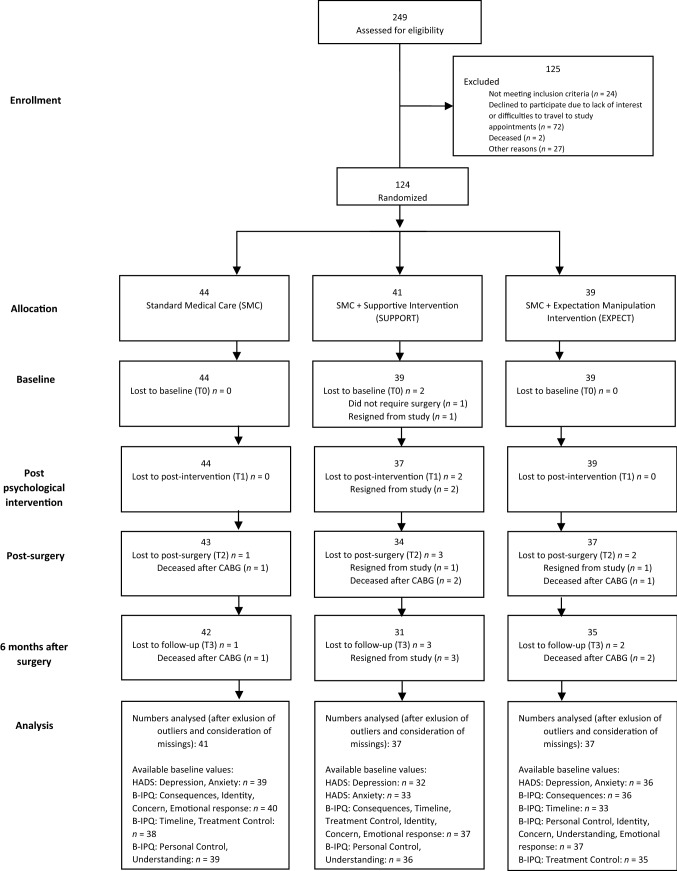
Flow chart (CONSORT). Study’s flow chart of the PSY-HEART trial (Rief et al., 2017). The “Analysis” is adapted to this articles aim (analyzing the Hospital Anxiety and Depression Scale (HADS) (Zigmond & Snaith, 1983) and the Brief Illness Perception Questionnaire (B-IPQ) (Broadbent et al., 2006)

In Fig. 1, the CONSORT flow-chart shows the recruitment and the count of participated patients for each measurement time point (Rief et al., 2017).

The calculated sample size for the primary analysis was *N* = 180 (time by group interaction, *f* = 0.2, *α* = 0.05, 1 − *β* = 0.8) (Rief et al., 2017). Recruitment goal was not achieved and the trial ended with *N* = 124 participants, this reflects a post hoc power of 85% (*f* > 0.15/*d* > 0.30/number needed to treat < 6, *α* = 0.05). After excluding 9 patients (for details, please see statistics), a sample size of *N* = 115 resulted. A post hoc power analysis with a sample size of *N* = 115 resulted in a post hoc power of 81% (*f* > 0.15/*d* > 0.30/number needed to treat < 6, *α* = 0.05). Since the Helsinki recommendation implies that trials investigating innovative interventions should not be oversized, this was considered adequate.

### Procedure

After having been contacted and consenting to participate, patients were informed about the study both orally and in writing at least three to 14 days before surgery. Before participation patients had to sign the informed consent.

At baseline measurement, psychologists performed the Structured Clinical Interview for DSM-IV (First et al., 1996) to screen for psychiatric comorbidities. Medical information as the EuroScore were taken out of the patient’s files. Afterwards, patients completed questionnaires, and blood samples were taken. More information about the questionnaires can be found in the Study Protocol (Laferton et al., 2013; Rief et al., 2017). For more information about the blood samples, please refer to Salzmann, Euteneuer, et al. (2020).

Patients were then randomized into one of the three intervention groups following a permuted block randomization in WINPEPI (block size of 9) (Abramson, 2011). Strata were age (≤ 65 years and more than 65 years) and the New Heart Association class (NYHA, I/II and III/IV). The psychologists opened one closed envelope for each patient when a patient had finished the baseline assessments to avoid bias. Patients and the study team were aware of the treatment allocation, while surgeons and all other routine care personal were blinded. Generally, patients randomized to one of the two intervention groups had their first session with the psychologist subsequently.

### Psychological interventions

Patients were randomized into one of three groups: either the SMC alone (control group) or one of two additional preoperative psychological interventions [EXPECT (IG) or SUPPORT (attention control group)]. Both psychological interventions lasted at least around 140 min (one individual session á 50 min, two phone calls á 20 min and another individual session á 50 min). They both were conducted by three clinical psychologists (2 male, 1 female) with advanced cognitive behavioral therapy skills. The manual-based interventions had a high treatment fidelity (Laferton et al., 2016). EXPECT focused on positive and realistic expectations regarding the patients’ disease, surgery-benefits, and recovery process. The EXPECT intervention is based on the Common Sense Model described in the introduction, with expectations as an inherent component of illness beliefs (Cameron & Leventhal, 2003). The EXPECT intervention focuses on several expectation facets. These expectations are summarized in the integrative model of expectations in patients undergoing medical treatment (Laferton et al., 2017). In particular, the intervention aimed to encourage the patients in developing a realistic understanding of disease and positive outcome expectations (separated in behavior-related expectations as self-efficacy and behavior outcome expectation and treatment-related expectations as structural and process expectations). Patients received psychoeducation about the CABG-procedure (structural expectations), planned when they will be able to return to which positive activities (process expectations), how they can influence controllable risk factors (behavior outcome expectation), and cope with handling side effects of the surgery (self-efficacy). A better understanding of patients’ disease was achieved, and false assumptions were corrected. In the end, patients imagined a positive scene after long-term recovery to strengthen their outcome expectations. Detailed information about the intervention, work sheets and examples of patients’ thoughts can be found elsewhere (Salzmann et al., 2018).

By comparing the intervention group EXPECT and the SMC control group, it would still be unknown if the outcome effects are due to the specific content of the EXPECT intervention (working on patients’ expectations) or are the result of the unspecific intervention ’ingredients’ such as establishing a therapeutic relationship, providing emotional support, and paying attention to the patient. Therefore, an active control group controlling for these unspecific effects (attention control group) was included. The recommended procedure for trials of psychological interventions includes realizing an attention control group in which patients receive the same amount of time and attention as the patients in the intervention group (Guidi et al., 2018). For this reason, the attention control group SUPPORT was implemented in the study. In the SUPPORT intervention, patients received the same amount of time, attention, and emotional support from the therapists as did the patients in the EXPECT group, but without specifically targeting expectations. Since attention and emotional support can lead to positive outcome effects by itself (Guidi et al., 2018), and the primary analysis of the PSY-HEART trial indicated some beneficial effects of the SUPPORT intervention compared to the SMC group (Rief et al., 2017), the attention control group SUPPORT could also be seen as another psychological intervention group. In sum, both preoperative psychological intervention groups provided the patients with emotional support, while only the EXPECT intervention specifically targeted patients’ expectations and illness beliefs which have been described as relevant mediators in the CSM.

### Outcome criteria

The primary outcome of the main trial was illness-related disability measured with an adapted version of the Pain Disability index (PDI) (Tait et al., 1990). Secondary outcomes were quality of life (Short Form 12, SF-12) (Ware et al., 1996), anxiety and depressive symptoms (Hospital Anxiety and Depression Scale, HADS) (Zigmond & Snaith, 1983), illness beliefs (Brief Illness Perception Questionnaire, B-IPQ) (Broadbent et al., 2006), subjective ability to work and the increase of metabolic equivalents of physical activity after surgery (International Physical Activity Questionnaire, IPAQ) (Craig et al., 2003). Results of these and other outcomes are published elsewhere (Rief et al., 2017). As this study examines further results and moderators for change in depression, anxiety and illness perceptions, these variables will be described in more detail.

The HADS examines anxiety and depression in patients with (psycho)somatic conditions. Each subscale has seven items that are scored at a 4-Item-Likert-Scale (0 to 3). Each scale was evaluated on its own and a sum score of the general psychological distress was analyzed (Zigmond & Snaith, 1983). Higher values mean a higher score of anxiety, depression or general psychological distress.

The B-IPQ surveys the cognitive and emotional representations of illness (Broadbent et al., 2006). It is composed of 8 closed items (range from 0 to 10). One additional question asks for possible reasons for the disease. Each item is evaluated individually. Higher scores reflect an increase of the respective dimension. Item 1–5 measure cognitive illness representations (consequences, timeline, personal control, treatment control, and identity). Item 6 and 8 quantify emotional representations (concern and emotions). Item 7 assesses illness comprehensibility. Item 9 is an open question (three most important causal factors in their illness).

### Statistics

As can be seen in Fig. 1, 9 patients were excluded from the ITT-sample for statistical analysis (violation of design requirements: 1; did not require CABG surgery: 1; resigned from study before baseline assessment: 1; multivariate outliers: 6; for details, please see Rief et al., 2017). Boxplots were screened to identify outliers (> 3 interquartile ranges). Only one outlier was identified for a T1-score of B-IPQ-Understanding, it was excluded before analysis. The following baseline scores were available: HADS – Depression: *n* = 107, HADS – Anxiety: *n* = 108, BIPQ – Identity, concern and emotional response: *n* = 114, B-IPQ – Consequences: *n* = 113, B-IPQ – Personal control and understanding: *n* = 112, B-IPQ – Treatment control: *n* = 110, B-IPQ – Timeline: *n* = 108.

To examine the psychological interventions effects as well as the potential moderation effects of baseline anxiety, depressive symptoms and illness beliefs on the intervention effects, multilevel models were used. Each outcome variable was explored in a separate model. Fixed effects were calculated for group (EXPECT, SMC, SUPPORT), time (1-day pre-surgery, 1-week post-surgery, 6 months post-surgery), baseline scores of the respective outcomes, group*time*baseline-scores and all other lower interaction terms (especially group*time-interactions). The analyses were adjusted for baseline differences, as the baseline scores were considered as a covariate.

In each model, a random intercept was included to allow for interindividual effects. First, two-way-interactions were examined (group*time) and in case of significant two-way-interactions post-hoc simple slope analyses were evaluated (*α* ≤ 0.05). Second, three-way-interactions were examined (group*time*baseline score of outcome variable). If significant or three-way-interactions were found, for continuous moderators simple-slope follow-up analyses were evaluated (Preacher et al., 2006). By doing so the significance of the intervention effects for conditional values of the moderator [for low (− 1 SD), average (mean) and high (+ 1 SD) baseline scores] were calculated.

Due to the small sample size missing data were imputed using restricted maximum likelihood (REML) methods. Co-variance structures were theoretically assumed in a first step and then empirically verified by goodness of fit using the Akaike information criterion (*AIC*) (Hox et al., 2018). For all statistical analyses SPSS 26 was used (IBM Corp., 2019). Due to the explored character of the article, we did not correct for multiple testing.

## Results

### Baseline characteristics

Table 1 shows the baseline characteristics as published in Rief et al. (2017), supplemented with the baseline levels of the outcome criteria of the analyses in this article. At baseline the groups did not differ significantly.

**Table 1:** Demographical, medical and psychological characteristics at baseline of patients receiving Standard Medical Care (SMC), Supportive Intervention (SUPPORT) or Expectation Manipulation Intervention (EXPECT) (Rief et al., 2017)

	SMC	SUPPORT	EXPECT	
Age in years, *M* (*SD*)	67.07 (8.9)	64.62 (8.1)	65.76 (7.8)	
Sex, male, *n* (%)	36 (87.8)	30 (81)	32 (86.5)	
Education, high school, *n* (%; MD = 1)	7 (17.1)	10 (27)	10 (27)	
Marital status, married, *n* (%; MD = 1)	33 (80.5)	34 (91.9)	31 (83.8)	
BMI, *M* (*SD*) (MD = 3)	29.67 (5.2)	29.5 (6.6)	29.03 (5.01)	
Smoking status, *n* (%)	6 (14.6)	2 (5.4)	6 (16.2)	
EuroSCORE II, *M* (*SD*) (MD = 11)	1.53 (0.8)	1.47 (0.8)	1.25 (0.8)	
NYHA, *n* (%) (MD = 10)				
I	1 (2.4)	1 (2.7)	0 (0)	
II	9 (22.0)	11 (29.7)	12 (32.4)	
III	28 (68.3)	20 (54.1)	17 (45.9)	
IV	1 (2.4)	2 (5.4)	3 (8.1)	
LVEF *n*, (%) (MD = 9)				
≥ 50	23 (48.8)	19 (51.4)	30 (78.4)	
49–30	13 (31.7)	13 (35.1)	4 (10.8)	
< 30	2 (4.9)	2 (5.4)	0 (0)	
Previous myocardial infarction, *n* (%) (MD = 5)	9 (23.1)	6 (17.1)	6 (16.7)	
Combined surgery, *n* (%)	6 (14.6)	6 (16.2)	3 (8.1)	
Anxiety, *M* (*SD*) (MD = 7)	4.03 (3.0)	4.55 (3.2)	5.17 (4.0)	
Depression, *M* (*SD*) (MD = 8)	4.59 (3.1)	4.0 (3.1)	5.11 (4.0)	
Consequences, *M* (*SD*) (MD = 2)	5.00 (2.96)	5.00 (3.38)	5.33 (3.03)	
Timeline, *M* (*SD*) (MD = 7)	3.03 (3.04)	3.95 (3.26)	1.39 (1.35)	
Personal Control, *M* (*SD*) (MD = 3)	4.64 (2.57)	4.31 (3.19)	3.84 (2.88)	
Treatment Control, *M* (*SD*) (MD = 5)	8.84 (1.42)	8.65 (1.51)	9.31 (0.90)	
Identity, *M* (*SD*) (MD = 1)	4.33 (2.71)	4.81 (2.94)	4.86 (2.64)	
Concern, *M* (*SD*) (MD = 1)	5.95 (3.06)	6.46 (3.23)	5.84 (3.71)	
Understanding, *M* (*SD*) (MD = 4)	6.72 (2.60)	7.00 (3.31)	7.30 (2.73)	
Emotional response, *M* (*SD*) (MD = 1)	4.43 (2.63)	4.41 (3.29)	4.70 (3.27)	

*Notes*. SMC = Standard Medical Care. SUPPORT = Supportive Intervention. EXPECT = Expectation Manipulation Intervention. MD = missing data. Body Mass Index (BMI). EuroSCORE (European System for Cardiac Operative Risk Evaluation). NYHA (New York Heart Association functional classification. LVEF (Left ventricular ejection fraction). Anxiety and Depression (Hamilton Anxiety and Depression Scale; HADS) range = 0–21. Disability (Pain Disability Index; PDI) range = 0–70. Mental quality of Life (Mental component of the Short-Form Health Survey; SF-12). Physical quality of Life (Physical component of the Short-Form Health Survey; SF-12). Physical activity (International physical activity questionnaire (IPAQ) weighted estimate of total physical activity per week. Cardiac Anxiety (Cardiac Anxiety Questionnaire) range = 0–4. Consequences, Timeline, Personal Control, Treatment Control, Identity, Concern, Understanding, Emotional response (Brief Illness Perception Questionnaire, B-IPQ) range 0–10

### Intervention effects over time

Two-way interactions were assessed to test for intervention effects over time (Table 2). Significant group by time-interactions were indicated for consequences (*p* = 0.028), personal control (*p* = 0.028), identity (*p* = 0.044) and concern (*p* = 0.030). No significant two-way interactions were observed for depression (*p* = 0.371), anxiety (*p* = 0.583), HADS sum score (*p* = 0.800), timeline (*p* = 0.588), treatment control (*p* = 0.165), understanding (*p* = 0.150) and emotional response (*p* = 0.335).

**Table 2:** Outcome measures at baseline, 1 day before surgery, 1 week after surgery and 6 months after surgery of patients receiving standard medical care (SMC), supportive intervention (SUPPORT) or expectation manipulation intervention (EXPECT) (observed measures) and test statistics for two-way interactions between intervention group and assessment time and also three-way-interactions between intervention group, assessment time and baseline score

	SMC *M (SD)*	SUPPORT *M (SD)*	EXPECT *M* (*SD*)	Test statistic (F scores of two-way interaction terms)	Test statistic (F scores of three-way interaction terms)
HADS					
Depressive symptoms				*F*(4, 163.827) = 1.075, *p* = .371	***F*(4, 162.184) = 2.569, *p* = .040**
Baseline	4.54	4.54	4.54		
1 day before surgery	5.00 (0.79)	4.21 (0.80)	4.77 (0.81)		
1 week after surgery	5.40 (0.79)	3.64 (0.83)	4.70 (0.79)		
6 months after surgery	3.65 (0.78)	2.12 (0.83)	2.48 (0.81)		
Anxiety				*F*(4, 190.192) = 0.714, *p* = .583	*F*(4, 181.434) = 1.547, *p* = .191
Baseline	4.55	4.55	4.55		
1 day before surgery	5.03 (0.44)	4.72 (0.46)	4.98 (0.47)		
1 week after surgery	4.18 (0.45)	3.06 (0.48)	3.53 (0.45)		
6 months after surgery	3.36 (0.43)	2.57 (0.47)	3.23 (0.45)		
Total score				*F*(4, 133.152) = 0.411, *p* = .800	*F*(4, 136.404) = 1.178, *p* = .323
Baseline	8.94	8.94	8.94		
1 day before surgery	10.01 (0.67)	8.68 (0.74)	9.34 (0.73)		
1 week after surgery	9.68 (0.74)	6.35 (0.84)	8.02 (0.76)		
6 months after surgery	7.01 (0.68)	4.42 (0.81)	5.20 (0.74)		
B-IPQ					
Consequences				***F*(4, 196.677) = 2.791, *p* = .028**	*F*(4, 194.173) = 2.215, *p* = .069
Baseline	5.09	5.09	5.09		
1 day before surgery	5.22 (0.51)	4.94 (0.52)	5.64 (0.53)		
1 week after surgery	6.97 (0.52)	6.36 (0.53)	6.50 (0.53)		
6 months after surgery	3.28 (0.51)	3.10 (0.53)	2.82 (0.53)		
Timeline				*F*(4, 121.741) = 0.708, *p* = .588	*F*(4, 121.999) = 0.541, *p* = .706
Baseline	2.65	2.65	2.65		
1 day before surgery	2.86 (0.29)	2.44 (0.32)	3.11 (0.43)		
1 week after surgery	4.82 (0.39)	4.61 (0.43)	3.73 (0.56)		
6 months after surgery	4.64 (0.55)	4.34 (0.62)	6.96 (0.94)		
Personal control				***F*(4, 127.549) = 2.805, *p* = .028**	***F*(4, 127.373) = 2.511, *p* = .045**
Baseline	4.41	4.41	4.41		
1 day before surgery	3.83 (2.09)	4.96 (2.09)	5.84 (2.09)		
1 week after surgery	5.45 (2.09)	6.05 (2.10)	6.22 (2.09)		
6 months after surgery	4.23 (2.10)	5.02 (2.10)	4.71 (2.11)		
Treatment control				*F*(4, 117.891) = 1.654, *p* = .165	*F*(4, 117.259) = 1.308, *p* = .271
Baseline	8.98	8.98	8.98		
1 day before surgery	8.83 (0.24)	8.58 (0.25)	8.86 (0.32)		
1 week after surgery	8.02 (0.27)	8.69 (0.28)	8.39 (0.29)		
6 months after surgery	7.77 (0.38)	8.77 (0.42)	7.37 (0.45)		
Identity				***F*(4, 152.844) = 2.519, *p* = .044**	*F*(4, 152.141) = 2.216, *p* = .070
Baseline	4.60	4.60	4.60		
1 day before surgery	3.97 (3.70)	4.21 (3.70)	4.58 (3.70)		
1 week after surgery	5.56 (3.71)	4.44 (3.72)	5.53 (3.71)		
6 months after surgery	3.22 (3.71)	2.67 (3.71)	2.27 (3.71)		
Concern				***F*(4, 152.853) = 2.763, *p* = .030**	***F*(4, 150.618) = 2.492, *p* = .046**
Baseline	6.15	6.15	6.15		
1 day before surgery	5.93 (1.01)	5.71 (1.02)	6.06 (1.01)		
1 week after surgery	5.74 (1.04)	4.62 (1.05)	4.75 (1.03)		
6 months after surgery	3.40 (1.01)	3.23 (1.03)	3.15 (1.02)		
Understanding				*F*(4, 182.219) = 1.706, *p* = .150	*F*(4, 181.222) = 1.337, *p* = .258
Baseline	7.10	7.10	7.10		
1 day before surgery	7.11 (0.54)	7.74 (0.56)	8.25 (0.55)		
1 week after surgery	7.31 (0.54)	7.60 (0.56)	7.55 (0.53)		
6 months after surgery	7.06 (0.54)	7.99 (0.56)	7.20 (0.54)		
Emotional response				*F*(4, 165.681) = 1.149, *p* = .335	*F*(4, 162.035) = 0.845, *p* = .498
Baseline	4.56	4.56	4.56		
1 day before surgery	4.42 (1.19)	3.99 (1.20)	4.64 (1.20)		
1 week after surgery	4.55 (1.22)	4.44 (1.23)	4.20 (1.22)		
6 months after surgery	2.84 (1.19)	2.50 (1.20)	2.20 (1.20)		

Intervention groups: Standard Medical Care (SMC), Supportive Intervention (SUPPORT) or Expectation Manipulation Intervention (EXPECT); Assessment times (adjusted for baseline scores): 1 day pre-surgery, 1 week post-surgery and 6 months after surgery. Statistically significant results are displayed in bold

The significant group by time-interaction of perceived personal control implied an intervention effect over time (*p* = 0.028, Table 2). Post-hoc simple slope analyses indicated that patients receiving EXPECT or SUPPORT showed significant higher personal control values 1 day before surgery compared to SMC (EXPECT vs. SMC: *p* < 0.001, SUPPORT vs. SMC: *p* = 0.045). There were no statistically significant differences at other measurement timepoints for personal control (*p* ≥ 0.127). The significant group by time-interaction of perceived consequences (*p* = 0.028, Table 2), perceived identity (*p* = 0.044, Table 2) and perceived concern (*p* = 0.030, Table 2) implied intervention effects over time. However, no significant group differences were indicated in post-hoc simple slope analyses for all three outcomes (consequences: *p* ≥ 0.192, identity: *p* ≥ 0.060, concern: *p* ≥ 0.101). The results of the significant group by time-interactions are diagrammed in the Supplementary Fig. 5.

### Moderation effects of baseline scores

Three-way interactions were assessed to test for moderating effects of the baseline score regarding the intervention effects (Table 2). Significant group by time by baseline scores of the outcome-interactions were indicated for depressive symptoms (*p* = 0.040), personal control (*p* = 0.045), and concern (*p* = 0.046). No significant three-way interactions were observed for anxiety (*p* = 0.191), HADS sum score (*p* = 0.323), consequences (*p* = 0.069), timeline (*p* = 0.706), treatment control (*p* = 0.271), identity (*p* = 0.070), understanding (*p* = 0.258) and emotional response (*p* = 0.498). The model fit statistics are accessible in Table 1 of the Supplementary material. The full results of the analyses were included in the Supplementary material.

### Moderation effects of baseline depressive symptoms

**Fig. 2:**
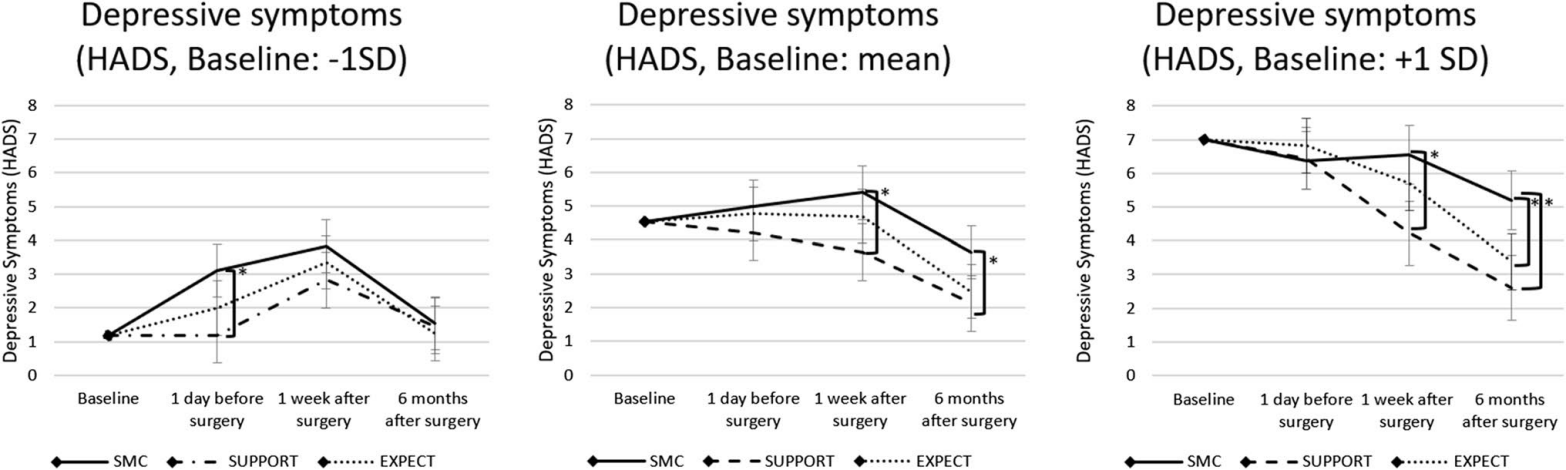
Post-hoc tests comparing intervention groups. Patients values of depressive symptoms (HADS) for low (− 1 SD), average (mean) and high (+ 1 SD) baseline rates receiving SMC, SUPPORT or EXPECT at baseline, 1 day before surgery, 1 week after surgery and 6 months after surgery. *p < 0.05

The baseline score of depressive symptoms (HADS) moderated the intervention effects on depressive symptoms. The significant group by time by baseline depressive symptoms-interaction implied this moderation (*p* = 0.040, Table 2). For all baseline scores of depressive symptoms significant group differences were reported in post-hoc simple slope analyses (see Fig. 2): Patients with high baseline scores of depressive symptoms (+ 1 SD) receiving SUPPORT or EXPECT showed significant lower scores of depressive symptoms 6 months after surgery compared to the control group (EXPECT vs. SMC: *p* = 0.015, SUPPORT vs. SMC: *p* = 0.004). 1 week after surgery patients receiving SUPPORT also showed significant lower scores of depressive symptoms compared to SMC (*p* = 0.009), while there were no significant differences for EXPECT vs. SMC (*p* = 0.246). No statistically significant differences were observed 1 day before surgery for high baseline scores of depressive symptoms. There were no statistically significant differences between the intervention groups (*p* ≥ 0.077). Patients with average scores of depressive symptoms at baseline (mean) receiving SUPPORT showed significant lower scores of depressive symptoms 1 week (*p* = 0.007) and also 6 months (*p* = 0.020) after surgery but not 1 day before surgery (*p* = 0.201) compared to SMC, while there were no significant differences for EXPECT vs. SMC at any time (1 day pre-surgery: *p* = 0.741, 1 week post-surgery: *p* = 0.243, 6 months post-surgery: *p* = 0.057). No significant group differences were found between EXPECT and SUPPORT (*p* ≥ 0.105). Patients with low baseline scores of depressive symptoms (− 1 SD) receiving SUPPORT showed significant lower scores of depressive symptoms 1 day before surgery compared to the control group (SMC; *p* = 0.028). No statistically significant differences were observed regarding the other measurement time points for low baseline scores of depressive symptoms (SUPPORT vs. SMC: 1 week post-surgery: *p* = 0.255, 6 months post-surgery: *p* = 0.927; EXPECT vs. SMC: 1 day pre-surgery: *p* = 0.217, 1 week post-surgery: *p* = 0.564, 6 months post-surgery: *p* = 0.735). There were no statistically significant differences between the intervention groups (*p* ≥ 0.363). Confidence intervals and further details of post-hoc tests can be found in the Supplementary Table 2.

### Moderation effects of baseline personal control

**Fig. 3:**
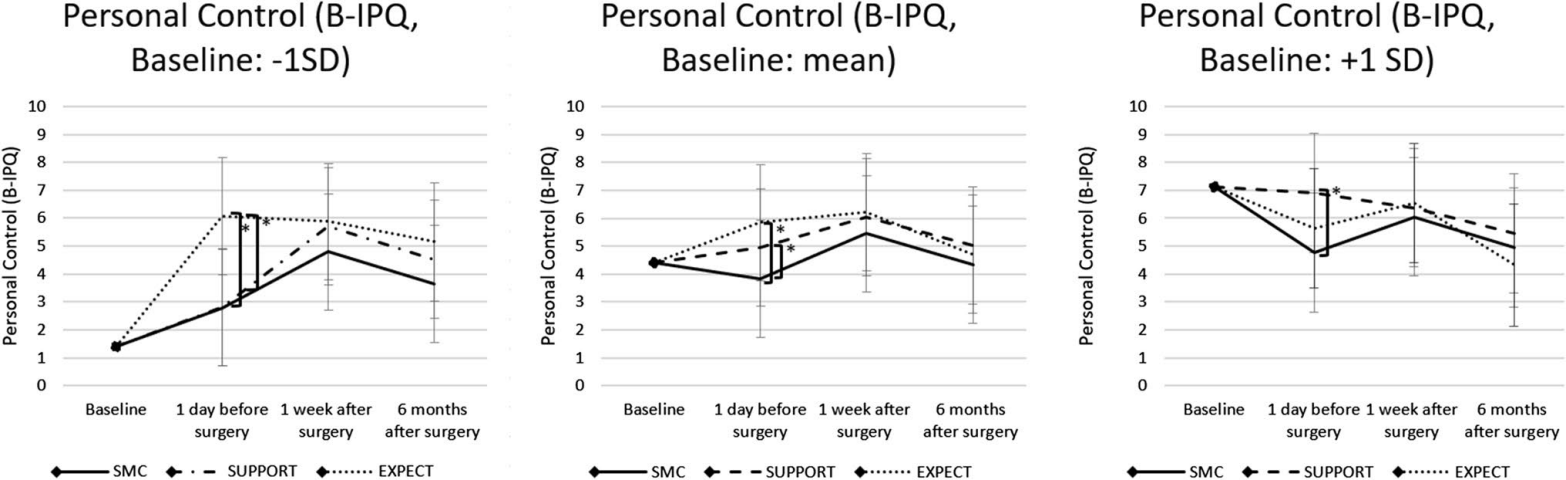
Post-hoc tests comparing intervention groups. Patients scores of personal control (B-IPQ) for low (− 1 SD), average (mean) and high (+ 1 SD) baseline values receiving SMC, SUPPORT or EXPECT at baseline, 1 day before surgery, 1 week after surgery and 6 months after surgery. *p < .05

Perceived personal control (B-IPQ) at baseline moderated the effects of the preoperative psychological interventions significantly. The significant group by time by baseline level of personal-control-interaction indicated this moderation (*p* = 0.045, Table 2). For all baseline scores (low, average, high) of personal control post-hoc simple slope analyses indicated statistically significant group differences (see Fig. 3): Patients with a low baseline personal control score (− 1 SD) receiving EXPECT showed significant higher personal control scores 1 day before surgery compared to SMC and SUPPORT (both: *p* < 0.001), while there were no significant differences for SUPPORT vs. SMC (*p* = 0.964). No statistically significant differences were observed regarding the other measurement time points for low baseline personal control (*p* ≥ 0.127). Patients with an average personal control score at baseline (mean) receiving EXPECT or SUPPORT showed significant higher personal control values 1 day before surgery compared to SMC (EXPECT: *p* < 0.001, SUPPORT: *p* = 0.045). There were no statistically significant differences at other measurement timepoints for average baseline personal control (*p* ≥ 0.127). No significant group differences were found between EXPECT and SUPPORT (*p* ≥ 0.118). Patients with a high baseline personal control score (+ 1 SD) receiving SUPPORT showed significant higher personal control values 1 day before surgery compared to SMC (*p* = 0.005), while there were no significant differences for EXPECT vs. SMC (*p* = 0.284). No statistically significant differences were observed regarding the other measurement time points for high baseline personal control (*p* ≥ 0.118). There were no statistically significant differences between the intervention groups (*p* ≥ 0.118). Confidence intervals and further details of post-hoc tests can be found in the Supplementary Table 3.

### Moderation effects of baseline concern

**Fig. 4:**
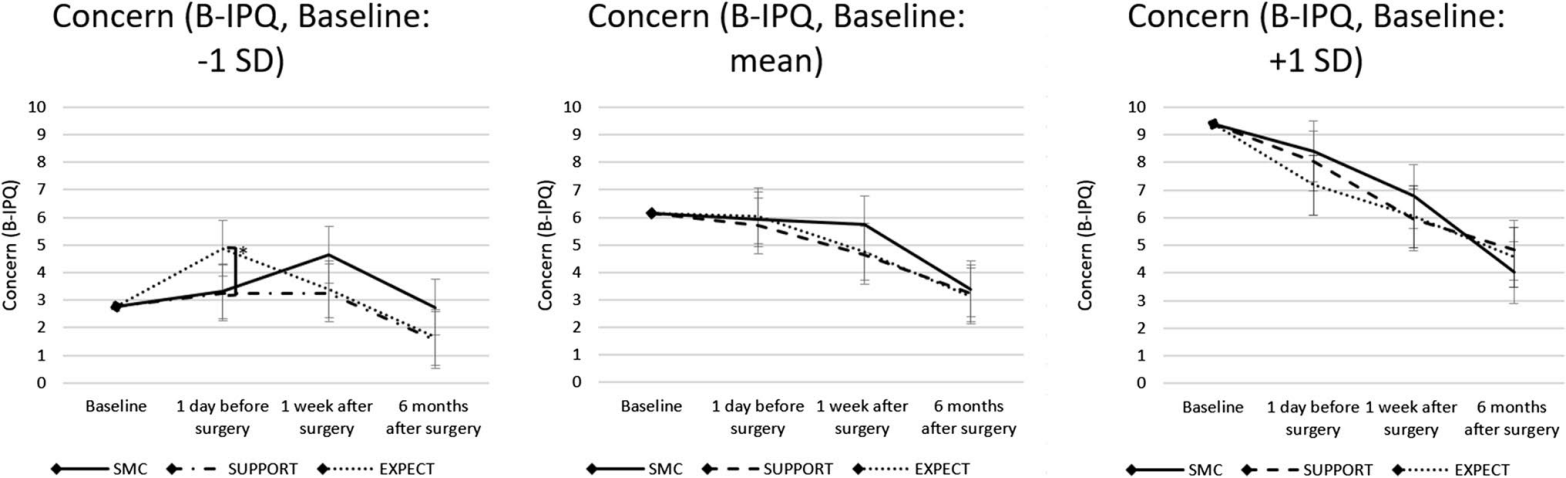
Post-hoc tests comparing intervention groups. Patients scores of concern (B-IPQ) for low (- 1 SD), average (mean) and high (+ 1 SD) baseline values receiving SMC, SUPPORT or EXPECT at baseline, 1 day before surgery, 1 week after surgery and 6 months after surgery. *p < .05

Baseline levels of perceived concern (B-IPQ) moderated the effects of the preoperative psychological interventions significantly. The significant group by time by baseline level of concern-interaction indicated this moderation (*p* = 0.046, Table 2). For low baseline scores of concern significant group differences were reported in post-hoc simple slope analyses (see Fig. 4): Patients with a low baseline concern score (− 1 SD) receiving EXPECT showed significant higher concern scores 1 day before surgery compared to SMC (*p* = 0.045), while there were no significant differences for SUPPORT versus SMC (*p* = 0.938) or SUPPORT vs. EXPECT (*p* = 0.052). No statistically significant differences were observed regarding the other measurement time points for low baseline concern (*p* ≥ 0.156). There were no statistically significant differences at any measurement timepoints for patients with an average (mean) or high (+ 1 SD) score of concern at baseline (*p* ≥ 0.101). Confidence intervals and further details of post-hoc tests can be found in the Supplementary Table 4.

### Additional analysis

To explore associations between our psychological outcome criteria (HADS and B-IPQ) and physical outcomes, we explored the correlations between our outcomes and the number of patients rehospitalized due to complications and the pump function of the heart (ejection fraction; EF) at 6 months follow-up. For instance, depressive symptom levels and concern 6 months after surgery were positively associated with the amount of patients reshospitalized due to complications (*r*_*depressive symptoms*_ = 0.214, *p* = 0.044; *r*_*concern*_ = − 0.293, *p* = 0.005). Rehospitalization scores after surgery were lowest in the EXPECT group (9% vs. 23% in the SUPPORT and 26% in the SMC group); however, this difference was not statistically significant (Rief et al., 2017). All results of the additional analysis can be found in the Supplementary Material Table 5.

## Discussion

This article aimed to explore whether baseline levels of patients’ depressive/anxiety symptoms and illness beliefs moderated the effects of preoperative psychological interventions (EXPECT/SUPPORT) on these constructs in heart surgery patients to develop a better understanding of whether and when psychological interventions may have an effect on these important outcomes in heart surgery patients.

Baseline levels of depressive symptoms, personal control, and concern seemed to moderate the intervention effects on depressive symptoms, personal control, and concern. Especially for patients with high baseline scores of depressive symptoms, both preoperative psychological interventions led to reduced levels of depressive symptoms 6 months after surgery compared to the control group SMC. Considering the Minimally Clinically Important Difference (MCID) of HADS scores for patients with cardiovascular disease that is ≥ 1.7 (Lemay et al., 2019), at least all discovered statistically significant effects of HADS were also clinically meaningful (≥ 1.763), except the discovered effect of SUPPORT and SMC for patients with average baseline levels of depressive symptoms at T3 (1.526). Also, some effects were found for illness beliefs, especially for personal control and concern: For a low personal control baseline-score the EXPECT-intervention led to increased personal control 1 day before surgery compared to SUPPORT and SMC. For patients with low baseline concern scores, the EXPECT-group indicated increased levels of concern 1 day before surgery compared to SMC. These results implicate that both preoperative psychological interventions may be especially relevant for patients with higher baseline depressive symptoms. Patients with lower baseline perceived personal control may benefit from the EXPECT-intervention.

As mentioned above, depression is a risk factor for many negative physical and psychological outcomes such as the higher risk of major adverse cardiac events, mortality, higher levels of medical complications, longer hospital stays, and lower quality of life after CABG surgery (AbuRuz, 2019; Auer et al., 2017; Blumenthal et al., 2003; Burg et al., 2003; Flaherty et al., 2017; McKenzie et al., 2010). Our study also indicated an association between depressive symptoms 6 months after surgery and postoperative complications leading to rehospitalization. Due to the importance of depression for psychological and physical outcomes, some studies tried to reduce depression in patients undergoing CABG surgery (Heilmann et al., 2016; McKenzie et al., 2010; Rollman, Belnap, LeMenager, Mazumdar, Houck et al., 2009; Rollman, Belnap, LeMenager, Mazumdar, Schulberg, & Reynolds III, 2009). The Bypassing the Blues (BtB) trial showed that CABG-patients who were depressed after their surgery profited from a collaborative care program (Rollman & Belnap, 2011). In the BtB-trial, patients participated after their surgery and had at least 1–28 contacts (median = 10) with the care manager in a period of eight months in the intervention groups. Compared to this trial, our results suggest that even a brief preoperative psychological intervention (five contacts) may be an effective way to decrease long-term levels of depressive symptoms and may improve heart surgery outcomes in patients with higher levels of depressive symptoms before surgery.

However, in our trial even most of the patients with ‘high’ baseline depressive symptoms did not reach the cut-off criterion for a clinical diagnosis of a depressive disorder (depression score ≥ 8) (Bjelland et al., 2002). Therefore, future research should examine how high a depressive burden has to be for letting patients benefit from a preoperative psychological intervention before undergoing surgery. Our results may not only be of scientific interest.

They may have important clinical implications: CABG-patients with significant depressive symptoms have almost twice the risk of having a cardiac event in the first 6 months after CABG-procedure (Scheier et al., 1999). In our trial, an association between depressive symptoms 6 months after surgery and complications leading to rehospitalization was found. Therefore, participation in a preoperative psychological intervention and ongoing support after the surgery (e.g., using booster calls) should be offered to these patients to improve psychological and physical outcomes. Given that further studies can replicate these findings, in line with the findings from Rollman and Belnap (2011), CABG patients should be screened for depressive symptoms before undergoing surgery. If depressed CABG-patients have a higher risk for adverse events such as cardiac events or mortality (Blumenthal et al., 2003; Scheier et al., 1999), patients with high levels of depressive symptoms who benefit from the individual surgery preparation would get the necessary assistance, while patients with lower levels (who may not benefit from the preoperative psychological interventions according to our results) would not need to take time for the intervention before and after surgery. Such a tailored treatment would provide every patient with the most profit and the fastest recovery possible. Also, the clinic and the healthcare system save capacities for the patients who need support and costs for less helpful interventions, or hospital stays (if the patients' recovery is faster and the time patients stay in hospital is shorter) (Auer et al., 2017; Oxlad et al., 2006).

Future reimbursement models will most probably focus more on outcome parameters including quality of life (value based medicine), than on diagnoses and procedures (DRG systematics). Accordingly, interventions that can positively impact the short and long-term outcomes may be worthwhile from medical and economic perspectives. The results for depression also showed that patients with high or average levels of baseline depressive symptoms receiving the SUPPORT intervention benefitted 1 week and 6 months after the CABG surgery. Patients with low levels of baseline depressive symptoms receiving the SUPPORT intervention benefitted 1 day before the CABG surgery. Thus, additional psychosocial support seems to be helpful.

No significant differences between EXPECT (optimizing expectations) and SUPPORT (focusing on emotional support) were found for depressive symptoms. Against the background of the CSM, this may be explained by the fact that depressive symptoms can be characterized as an emotional and interpersonal challenge and a cognitive alteration; patients suffering from depression indicate dysfunctional cognitions and maladaptive information processing (Beck, 1979). Both interventions included at least one placebo mechanism (optimizing expectations, empathic relationship between patient and provider) (Schedlowski et al., 2015) targeting these factors. This may have led to no significant differences between the intervention groups for depressive symptoms. Further research should focus on the question, which kind of intervention is the most helpful for patients with depressive symptoms to further elucidate crucial mechanisms.

Personal control as one of the illness beliefs seems to play an important part in the effects of preoperative psychological interventions. Previous studies indicated that personal control was most amenable to change compared to other illness belief dimensions (Broadbent et al., 2015; Laferton et al., 2016). In our trial, we found that receiving EXPECT or SUPPORT led to increased levels of personal control 1 day before surgery. Patients seemed to be more convinced to recover from or control their heart disease by their action after both preoperative psychological interventions than SMC patients. This could be due to the fact that patients pay attention to themselves and their heart disease in both interventions and therefore get the idea that their behavior influences their recovery. Furthermore, it is conceivable that patients in the SUPPORT group had the opportunity to self-reflect and may thus have come to the conclusion that their personal behavior may be part of their recovery. Since patients were not blind about their psychological intervention, receiving any kind of preoperative psychological intervention (compared to receiving only SMC) could have contributed to increased personal control levels in both psychological interventions.

The analyses indicated that patients with low perceived personal control levels at baseline benefitted from EXPECT in the short term compared to both other groups. Higher perceived personal control is associated with a higher quality of life and lower levels of depressive symptoms after CABG surgery (Kidd et al., 2016). Therefore, the increase of perceived personal control in our study might help avoid or reduce depressive symptoms in patients and hereby reduce physical outcomes as explained above. By now, only a short-term effect was found. Future studies should examine whether a sustained effect of increased personal control after surgery would have additional positive effects on long-term outcomes. Therefore, it should be examined, if more booster sessions can maintain the increase in perceived personal control for patients with lower baseline levels of personal control.

For patients with average and high baseline levels of personal control, the SUPPORT intervention led to increased levels of personal control 1 day before surgery. This finding may lead to the assumption that validation and emotional support may increase patients’ perceived controllability as someone strengthens the patients' confidence and trust in their thoughts and preparations.

Regarding patients’ level of concern, a short-term effect was observed for patients with low scores of baseline concern. In the EXPECT group, an increase of concern was observed after the intervention 1 day before surgery. By focusing on psychoeducational aspects in this group, it is not surprising that patients’ worries increased short-dated. This result may explain why no effect was found for anxiety. By focusing on realistic expectations, patients also discussed topics that may have been perceived as concerning.

Some limitations need to be considered when interpreting the results of the study. Patients were only included in the study when they could appear in the study hospital a few days before the planned surgery date. Therefore, only patients with enough interest, time, and the possibility to drive to the hospital (even if some lived far away) were included. These facts may limit the generalizability of the findings. When getting informed, patients received the information that three treatment groups are included in the study and that two of them will receive additional conversations. It is possible that the expectation of receiving “just the standard of care” or “something special, additional” may have affected the outcomes. Focusing on depressive symptoms, most of the patients did not reach the cut-off criterium (depression score ≥ 8) (Bjelland et al., 2002). Therefore, the patients in the trial were not depressed on a high level. Further, due to the explorative character of the analyses conducted, our findings should be interpreted with caution. No correction for multiple testing has been done. Multi-centered confirmatory trials including more patients are needed to confirm the findings, generalize from one study site to the general healthcare systems, investigate further clinical outcome variables, and gain more knowledge, who would benefit from which intervention. It would be important to replicate the findings with a larger sample focusing on physical and psychological symptoms such as hospital stay, mortality, rehospitalization, depression, anxiety, illness beliefs and their associations.

In conclusion, our findings indicate that some patients may benefit from preoperative interventions while others will not. This study indicated that brief preoperative psychological interventions might improve critical psychological outcomes such as depressive symptoms or personal control in some heart surgery patients, but not in all patients. It further indicated that this may especially apply to specific subgroups of patients (i.e., high baseline depressive symptoms, low baseline personal control). Patients’ psychological status at baseline may moderate the effectiveness of psychological interventions. The second important finding is that assessing baseline levels is essential to offer tailored psychological interventions to improve long-term heart surgery outcomes. Gathering patients’ psychological status before undergoing heart surgery and providing psychological interventions if they are indicated (e.g., for patients with high scores of depressive symptoms or low levels of perceived control) would be beneficial. More studies are needed to examine which patients may benefit from what kind of preoperative psychological intervention at which timepoint and why.

## Supplementary Information

Below is the link to the electronic supplementary material.

Supplementary file1 (DOCX 467 kb)

## Data Availability

Original data files and materials are stored at the division of clinical psychology. All authors had full access to the data. Data sets are available, as long as patients’ anonymity and ethical issues are respected.
