# Supplementary material for: Baseline depressive symptoms, personal control, and concern moderate the effects of preoperative psychological interventions: the randomized controlled PSY-HEART trial
Source: J Behav Med. 2022 May 6;45(3):350–65. doi: 10.1007/s10865-022-00319-0 (PMC9160109; doi:10.1007/s10865-022-00319-0)
Supplement: Supplementary file 1 — Supplementary file1 (DOCX 467 kb) [file 10865_2022_319_MOESM1_ESM.docx]

**Baseline depressive symptoms, personal control, and concern moderate the effects of preoperative psychological interventions – the randomized controlled PSY-HEART trial**

**Abbreviated running title: Moderators of psychological interventions in heart surgery**

**Supplementary materials.**


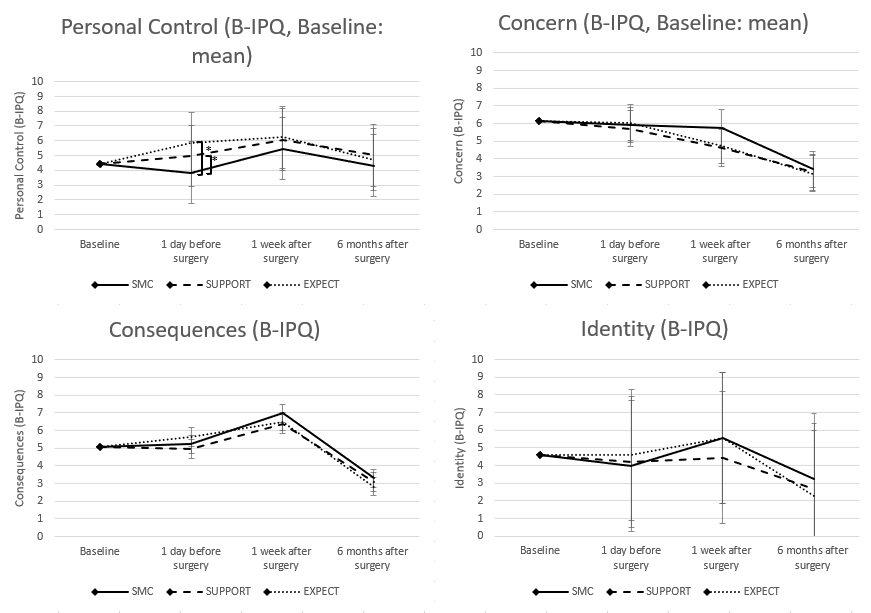


**Fig. 5.** Post-hoc tests of the two way interactions (group by time) comparing intervention groups. Patients values of personal control, concern, consequences and identity for average (mean) baseline rates receiving SMC, SUPPORT or EXPECT at baseline, 1 day before surgery, 1 week after surgery and 6 months after surgery. * = p < .05.

**Supplementary table 1. Model fit statistics.**

| Model fit statistics | | | |  | |
| --- | --- | --- | --- | --- | --- |
| Dependent Variable | -2 Log Likelihood | AIC | AICC | BIC | CAIC |
| **B-IPQ** |  |  |  |  |  |
| Consequences | 1320.230 | 1326.230 | 1326.316 | 1337.156 | 1340.156 |
| Timeline | 1274.874 | 1284.874 | 1285.106 | 1302.773 | 1307.773 |
| Personal Control | 1270.473 | 1280.473 | 1280.705 | 1298.372 | 1303.372 |
| Treatment Control | 1074.589 | 1084.589 | 1084.819 | 1102.506 | 1107.506 |
| Identity | 1307.346 | 1317.346 | 1317.562 | 1335.591 | 1340.591 |
| Concern | 1395.823 | 1405.823 | 1406.037 | 1424.103 | 1429.103 |
| Coherence | 1228.946 | 1234.946 | 1235.036 | 1245.775 | 1248.775 |
| Emotional response | 1397.883 | 1407.883 | 1408.096 | 1426.198 | 1431.198 |
| **HADS** |  |  |  |  |  |
| Depression | 1300.011 | 1306.011 | 1306.103 | 1316.739 | 1319.739 |
| Anxiety | 1337.693 | 1343.693 | 1343.783 | 1354.488 | 1357.488 |
| HADS sumscore | 1560.450 | 1570.450 | 1570.685 | 1588.273 | 1593.273 |

Notes: AIC = Akaike information criterion, BIC = Bayesian information criterion, AICC = Hurvich and Tsai’s Criterion, CAIC = Bozdogan’s Criterion. The information

criteria are displayed in smaller-is-better form.

| **Supplementary table 2. Post-hoc group comparisons for T1, T2 and T3 depressive symptoms and tests for change over time within each intervention group.**   \| *Estimates for level of depressive symptoms* (low (-1 SD)) \| \| \| \| \| \| \| \| --- \| --- \| --- \| --- \| --- \| --- \| --- \| \| Intervention group \| time \| Mean \| Std. Error \| df \| 95% Confidence Interval \| \| \| Lower Bound \| Upper Bound \| \| SMC \| 1 \| 3.119 \| .921 \| .000 \| -2.35 \| 8.596 \| \| 2 \| 3.834 \| .907 \| .000 \| -.640 \| 8.308 \| \|  \| 3 \| 1.541 \| .902 \| .000 \| -7.275 \| 1.356 \| \| SUPPORT \| 1 \| 1.183 \| .894 \| .000 \| -9.280 \| 11.645 \| \| 2 \| 2.836 \| .910 \| .000 \| -5.280 \| 1.951 \| \|  \| 3 \| 1.460 \| .928 \| .000 \| -4.288 \| 7.207 \| \| EXPECT \| 1 \| 1.990 \| .931 \| .000 \| -7.072 \| 11.051 \| \| 2 \| 3.343 \| .885 \| .000 \| -1.987 \| 8.672 \| \|  \| 3 \| 1.245 \| .912 \| .000 \| -4.716 \| 7.206 \| \| Notes: Dependent Variable: HADS_Depressive symptoms. Time 1 = 1 day pre-surgery; time 2 = 1 week post-surgery; time 3 = 6 months follow-up-assessment. Adjusted score T0 (baseline) = 1.18213. \| \| \| \| \| \| \|  \| *Pairwise Comparisons for level of depressive symptoms at T1, T2 and T3* (low(-1 SD)) \| \| \| \| \| \| \| \| \| \| --- \| --- \| --- \| --- \| --- \| --- \| --- \| --- \| --- \| \| time \| (I) Intervention group \| (J) Intervention group \| Mean Difference (I-J) \| Std. Error \| df \| Sig. \| 95% Confidence Interval for Difference \| \| \| Lower Bound \| Upper Bound \| \| 1 \| SMC \| SUPPORT \| 1.936* \| .874 \| 244.165 \| .028 \| .215 \| 3.658 \| \| EXPECT \| 1.130 \| .912 \| 251.449 \| .217 \| -.666 \| 2.926 \| \| SUPPORT \| SMC \| -1.936* \| .874 \| 244.165 \| .028 \| -3.658 \| -.215 \| \| EXPECT \| -.807 \| .885 \| 248.640 \| .363 \| -2.550 \| .936 \| \| EXPECT \| SMC \| -1.130 \| .912 \| 251.449 \| .217 \| -2.926 \| .666 \| \| SUPPORT \| .807 \| .885 \| 248.640 \| .363 \| -.936 \| 2.550 \| \| 2 \| SMC \| SUPPORT \| .998 \| .875 \| 243.673 \| .255 \| -.726 \| 2.723 \| \| EXPECT \| .491 \| .850 \| 241.522 \| .564 \| -1.183 \| 2.165 \| \| SUPPORT \| SMC \| -.998 \| .875 \| 243.673 \| .255 \| -2.723 \| .726 \| \| EXPECT \| -.507 \| .853 \| 242.523 \| .553 \| -2.187 \| 1.173 \| \| EXPECT \| SMC \| -.491 \| .850 \| 241.522 \| .564 \| -2.165 \| 1.183 \| \| SUPPORT \| .507 \| .853 \| 242.523 \| .553 \| -1.173 \| 2.187 \| \| 3 \| SMC \| SUPPORT \| .081 \| .890 \| 246.142 \| .927 \| -1.671 \| 1.834 \| \| EXPECT \| .296 \| .873 \| 245.531 \| .735 \| -1.423 \| 2.016 \| \| SUPPORT \| SMC \| -.081 \| .890 \| 246.142 \| .927 \| -1.834 \| 1.671 \| \| EXPECT \| .215. \| .900 \| 250.100 \| .811 \| -1.558 \| 1.988 \| \| EXPECT \| SMC \| -.296 \| .873 \| 245.531 \| .735 \| -2.016 \| 1.423 \| \|  \|  \| SUPPORT \| -.215 \| .900 \| 250.100 \| .811 \| -1.988 \| 1.558 \| \| Notes: Dependent Variable: HADS_Depressive symptoms. Based on estimated marginal means. Time 1 = T1 (1 day pre-surgery); time 2 = T2 (1 week post-surgery), time 3 = T3 (6 months follow-up-assessment). *. The mean difference is significant at the .05 level. \| \| \| \| \| \| \| \| \| |
| --- | --- | --- | --- | --- | --- | --- | --- | --- | --- | --- | --- | --- | --- | --- | --- | --- | --- | --- | --- | --- | --- | --- | --- | --- | --- | --- | --- | --- | --- | --- | --- | --- | --- | --- | --- | --- | --- | --- | --- | --- | --- | --- | --- | --- | --- | --- | --- | --- | --- | --- | --- | --- | --- | --- | --- | --- | --- | --- | --- | --- | --- | --- | --- | --- | --- | --- | --- | --- | --- | --- | --- | --- | --- | --- | --- | --- | --- | --- | --- | --- | --- | --- | --- | --- | --- | --- | --- | --- | --- | --- | --- | --- | --- | --- | --- | --- | --- | --- | --- | --- | --- | --- | --- | --- | --- | --- | --- | --- | --- | --- | --- | --- | --- | --- | --- | --- | --- | --- | --- | --- | --- | --- | --- | --- | --- | --- | --- | --- | --- | --- | --- | --- | --- | --- | --- | --- | --- | --- | --- | --- | --- | --- | --- | --- | --- | --- | --- | --- | --- | --- | --- | --- | --- | --- | --- | --- | --- | --- | --- | --- | --- | --- | --- | --- | --- | --- | --- | --- | --- | --- | --- | --- | --- | --- | --- | --- | --- | --- | --- | --- | --- | --- | --- | --- | --- | --- | --- | --- | --- | --- | --- | --- | --- | --- | --- | --- | --- | --- | --- | --- | --- | --- | --- | --- | --- | --- | --- | --- | --- | --- | --- | --- | --- | --- | --- | --- | --- | --- | --- | --- | --- | --- | --- | --- | --- | --- | --- | --- | --- | --- | --- | --- | --- | --- | --- | --- | --- | --- | --- | --- | --- | --- | --- | --- | --- | --- | --- | --- | --- | --- | --- | --- |

| *Pairwise Comparisons: change over time* *for level of depressive symptoms* (low (-1 SD)) | | | | | | | | |
| --- | --- | --- | --- | --- | --- | --- | --- | --- |
| Intervention group | (I) time | (J) time | Mean Difference (I-J) | Std. Error | df | Sig. | 95% Confidence Interval for Difference^c^ | |
|  |  |  |  |  |  |  | Lower Bound | Upper Bound |
| SMC | 1 | 2 | -.715 | .791 | 163.310 | .367 | -2.276 | .846 |
|  |  | 3 | 1.578* | .784 | 161.830 | .046 | .029 | 3.128 |
|  | 2 | 1 | .715 | .791 | 163.310 | .367 | -.846 | 2.276 |
|  |  | 3 | 2.293* | .768 | 158.595 | .003 | .776 | 3.810 |
|  | 3 | 1 | -1.578* | .784 | 161.830 | .046 | -3.128 | -.029 |
|  |  | 2 | -2.293* | .768 | 158.595 | .003 | -3.810 | -.776 |
| SUPPORT | 1 | 2 | -1.653* | .765 | 161.600 | .032 | -3.164 | -.141 |
|  |  | 3 | -.277 | .787 | 165.717 | .726 | -1.831 | 1.278 |
|  | 2 | 1 | 1.653* | .765 | 161.600 | .032 | .141 | 3.164 |
|  |  | 3 | 1.376 | .801 | 162.857 | .088 | -.205 | 2.957 |
|  | 3 | 1 | .277 | .787 | 165.717 | .726 | -1.278 | 1.831 |
|  |  | 2 | -1.376 | .801 | 162.857 | .088 | -2.957 | .205 |
| EXPECT | 1 | 2 | -1.353 | .786 | 169.321 | .087 | -2.905 | .199 |
|  |  | 3 | .745 | .818 | 176.910 | .364 | -.869 | 2.359 |
|  | 2 | 1 | 1.353 | .786 | 169.321 | .087 | -.199 | 2.905 |
|  |  | 3 | 2.098* | .762 | 162.640 | .007 | .594 | 3.603 |
|  | 3 | 1 | -.745 | .818 | 176.910 | .364 | -2.359 | .869 |
|  |  | 2 | -2.098* | .762 | 162.640 | .007 | -3.603 | -.594 |
| Notes: Dependent Variable: HADS_Depressive symptoms. Based on estimated marginal means. Time 1 = T1 (1 day pre-surgery); time 2 = T2 (1 week post-surgery), time 3 = T3 (6 months follow-up-assessment). *. The mean difference is significant at the .05 level. | | | | | | | | |

| *Estimates for level of depressive symptoms* (average (mean)) | | | | | | |
| --- | --- | --- | --- | --- | --- | --- |
| Intervention group | time | Mean | Std. Error | df | 95% Confidence Interval | |
|  |  |  |  |  | Lower Bound | Upper Bound |
| SMC | 1 | 4.998 | .785 | .000 | 2.111 | 7.885 |
|  | 2 | 5.402 | .788 | .000 | 2.138 | 8.666 |
|  | 3 | 3.649 | .782 | .000 | .201 | 7.097 |
| SUPPORT | 1 | 4.211 | .803 | .000 | .980 | 7.442 |
|  | 2 | 3.639 | .828 | .000 | -.367 | 7.645 |
|  | 3 | 2.123 | .834 | .000 | -1.648 | 5.895 |
| EXPECT | 1 | 4.771 | .806 | .000 | 1.069 | 8.474 |
|  | 2 | 4.699 | .790 | .000 | 1.760 | 7.639 |
|  | 3 | 2.479 | .805 | .000 | -1.470 | 6.429 |
| Notes: Dependent Variable: HADS_Depressive symptoms. Time 1 = 1 day pre-surgery; time 2 = 1 week post-surgery; time 3 = 6 months follow-up-assessment. Adjusted score T0 (baseline) = 4.5355. | | | | | | |

| *Pairwise Comparisons for level of depressive symptoms at T1, T2 and T3* (average (mean)) | | | | | | | | |
| --- | --- | --- | --- | --- | --- | --- | --- | --- |
| time | (I) Intervention group | (J) Intervention group | Mean Difference (I-J) | Std. Error | df | Sig. | 95% Confidence Interval for Difference | |
|  |  |  |  |  |  |  | Lower Bound | Upper Bound |
| 1 | SMC | SUPPORT | .787 | .614 | 243.587 | .201 | -.422 | 1.996 |
|  |  | EXPECT | .227 | .617 | 248.941 | .714 | -.990 | 1.443 |
|  | SUPPORT | SMC | -.787 | .614 | 243.587 | .201 | -1.996 | .422 |
|  |  | EXPECT | -.561 | .641 | 247.161 | .383 | -1.823 | .702 |
|  | EXPECT | SMC | -.227 | .617 | 248.941 | .714 | -1.443 | .990 |
|  |  | SUPPORT | .561 | .641 | 247.161 | .383 | -.702 | 1.823 |
| 2 | SMC | SUPPORT | 1.763* | .650 | 249.711 | .007 | .483 | 3.044 |
|  |  | EXPECT | .703 | .600 | 245.007 | .243 | -.479 | 1.885 |
|  | SUPPORT | SMC | -1.763* | .650 | 249.711 | .007 | -3.044 | -.483 |
|  |  | EXPECT | -1.061 | .652 | 247.649 | .105 | -2.345 | .224 |
|  | EXPECT | SMC | -.703 | .600 | 245.007 | .243 | -1.885 | .479 |
|  |  | SUPPORT | 1.061 | .652 | 247.649 | .105 | -.224 | 2.345 |
| 3 | SMC | SUPPORT | 1.525* | .650 | 249.674 | .020 | .245 | 2.806 |
|  |  | EXPECT | 1.170 | .613 | 247.628 | .057 | -.037 | 2.376 |
|  | SUPPORT | SMC | -1.525* | .650 | 249.674 | .020 | -2.806 | -.245 |
|  |  | EXPECT | -.356 | .678 | 252.145 | .600 | -1.690 | .978 |
|  | EXPECT | SMC | -1.170 | .613 | 247.628 | .057 | -2.376 | .037 |
|  |  | SUPPPORT | .356 | .678 | 252.145 | .600 | -.978 | 1.690 |
| Notes: Dependent Variable: HADS_Depressive symptoms. Based on estimated marginal means. Time 1 = T1 (1 day pre-surgery); time 2 = T2 (1 week post-surgery), time 3 = T3 (6 months follow-up-assessment). *. The mean difference is significant at the .05 level. | | | | | | | | |

| *Pairwise Comparisons: change over time* *for level of depressive symptoms* (average (mean)) | | | | | | | | |
| --- | --- | --- | --- | --- | --- | --- | --- | --- |
| Intervention group | (I) time | (J) time | Mean Difference (I-J) | Std. Error | df | Sig. | 95% Confidence Interval for Difference^c^ | |
|  |  |  |  |  |  |  | Lower Bound | Upper Bound |
| SMC | 1 | 2 | -.404 | .530 | 165.218 | .447 | -1.451 | .643 |
|  |  | 3 | 1.349* | .521 | 162.145 | .011 | .320 | 2.378 |
|  | 2 | 1 | .404 | .530 | 165.218 | .447 | -.643 | 1.451 |
|  |  | 3 | 1.753* | .526 | 163.883 | .001 | .714 | 2.792 |
|  | 3 | 1 | -1.349* | .521 | 162.145 | .011 | -2.378 | -.320 |
|  |  | 2 | -1.753* | .526 | 163.883 | .001 | -2.792 | -.714 |
| SUPPORT | 1 | 2 | .572 | .600 | 171.855 | .342 | -.613 | 1.757 |
|  |  | 3 | 2.087* | .608 | 173.759 | .001 | .888 | 3.287 |
|  | 2 | 1 | -.572 | .600 | 171.855 | .342 | -1.757 | .613 |
|  |  | 3 | 1.515* | .632 | 165.325 | .018 | .268 | 2.762 |
|  | 3 | 1 | -2.087* | .608 | 173.759 | .001 | -3.287 | -.888 |
|  |  | 2 | -1.515* | .632 | 165.325 | .018 | -2.762 | -.268 |
| EXPECT | 1 | 2 | .072 | .559 | 168.678 | .898 | -1.031 | 1.175 |
|  |  | 3 | 2.292* | .580 | 173.867 | <.001 | 1.146 | 3.437 |
|  | 2 | 1 | -.072 | .559 | 168.678 | .898 | -1.175 | 1.031 |
|  |  | 3 | 2.220* | .556 | 164.703 | <.001 | 1.123 | 3.317 |
|  | 3 | 1 | -2.292* | .580 | 173.867 | <.001 | -3.437 | -1.146 |
|  |  | 2 | -2.220* | .556 | 164.703 | <.001 | -3.317 | -1.123 |
| Notes: Dependent Variable: HADS_Depressive symptoms. Based on estimated marginal means. Time 1 = T1 (1 day pre-surgery); time 2 = T2 (1 week post-surgery), time 3 = T3 (6 months follow-up-assessment). *. The mean difference is significant at the .05 level. | | | | | | | | |

| *Estimates for level of depressive symptoms* (high (+ 1SD)) | | | | | | |
| --- | --- | --- | --- | --- | --- | --- |
| Intervention group | time | Mean | Std. Error | df | 95% Confidence Interval | |
|  |  |  |  |  | Lower Bound | Upper Bound |
| SMC | 1 | 6.382 | .864 | .000 | 2.700 | 1.064 |
|  | 2 | 6.557 | .872 | .000 | -14.558 | 27.672 |
|  | 3 | 5.202 | .864 | .000 | 1.368 | 9.025 |
| SUPPORT | 1 | 6.441 | .911 | .000 | -.756 | 13.639 |
|  | 2 | 4.230 | .948 | .000 | -3.901 | 12.361 |
|  | 3 | 2.613 | .967 | .000 | -3.809 | 9.034 |
| EXPECT | 1 | 6.820 | .814 | .000 | 2.800 | 1.841 |
|  | 2 | 5.699 | .817 | .000 | 2.265 | 9.132 |
|  | 3 | 3.389 | .827 | .000 | .094 | 6.684 |
| Notes: Dependent Variable: HADS_Depressive symptoms. Time 1 = 1 day pre-surgery; time 2 = 1 week post-surgery; time 3 = 6 months follow-up-assessment. Adjusted score T0 (baseline) = 7.00547. | | | | | | |

| *Pairwise Comparisons for level of depressive symptoms at T1, T2 and T3* (high (+1 SD)) | | | | | | | | |
| --- | --- | --- | --- | --- | --- | --- | --- | --- |
| time | (I) Intervention group | (J) Intervention group | Mean Difference (I-J) | Std. Error | df | Sig. | 95% Confidence Interval for Difference | |
|  |  |  |  |  |  |  | Lower Bound | Upper Bound |
| 1 | SMC | SUPPORT | -.059 | .832 | 242.249 | .943 | -1.698 | 1.580 |
|  |  | EXPECT | -.439 | .725 | 243.300 | .546 | -1.867 | .990 |
|  | SUPPORT | SMC | .059 | .832 | 242.249 | .943 | -1.580 | 1.698 |
|  |  | EXPECT | -.379 | .780 | 242.243 | .627 | -1.916 | 1.157 |
|  | EXPECT | SMC | .439 | .725 | 243.300 | .546 | -.990 | 1.867 |
|  |  | SUPPORT | .379 | .780 | 242.243 | .627 | -1.157 | 1.916 |
| 2 | SMC | SUPPORT | 2.327^*^ | .881 | 248.549 | .009 | .592 | 4.061 |
|  |  | EXPECT | .859 | .738 | 245.955 | .246 | -.594 | 2.312 |
|  | SUPPORT | SMC | -2.327^*^ | .881 | 248.549 | .009 | -4.061 | -.592 |
|  |  | EXPECT | -1.468 | .826 | 248.315 | .077 | -3.095 | .158 |
|  | EXPECT | SMC | -.859 | .738 | 245.955 | .246 | -2.312 | .594 |
|  |  | SUPPORT | 1.468 | .826 | 248.315 | .077 | -.158 | 3.095 |
| 3 | SMC | SUPPORT | 2.589^*^ | .892 | 250.205 | .004 | .832 | 4.346 |
|  |  | EXPECT | 1.813^*^ | .738 | 245.869 | .015 | .358 | 3.267 |
|  | SUPPORT | SMC | -2.589^*^ | .892 | 250.205 | .004 | -4.346 | -.832 |
|  |  | EXPECT | -.776 | .857 | 252.653 | .366 | -2.464 | .911 |
|  | EXPECT | SMC | -1.813^*^ | .738 | 245.869 | .015 | -3.267 | -.358 |
|  |  | SUPPORT | .776 | .857 | 252.653 | .366 | -.911 | 2.464 |
| Notes: Dependent Variable: HADS_Depressive symptoms. Based on estimated marginal means. Time 1 = T1 (1 day pre-surgery); time 2 = T2 (1 week post-surgery), time 3 = T3 (6 months follow-up-assessment). *. The mean difference is significant at the .05 level. | | | | | | | | |

| *Pairwise Comparisons: change over time* *for level of depressive symptoms* (high (+1 SD)) | | | | | | | | |
| --- | --- | --- | --- | --- | --- | --- | --- | --- |
| Intervention group | (I) time | (J) time | Mean Difference (I-J) | Std. Error | df | Sig. | 95% Confidence Interval for Difference^c^ | |
|  |  |  |  |  |  |  | Lower Bound | Upper Bound |
| SMC | 1 | 2 | -.176 | .703 | 162.842 | .803 | -1.564 | 1.213 |
|  |  | 3 | 1.180 | .692 | 160.108 | .090 | -.187 | 2.547 |
|  | 2 | 1 | .176 | .703 | 162.842 | .803 | -1.213 | 1.564 |
|  |  | 3 | 1.355 | .702 | 162.662 | .055 | -.032 | 2.742 |
|  | 3 | 1 | -1.180 | .692 | 160.108 | .090 | -2.547 | .187 |
|  |  | 2 | -1.355 | .702 | 162.662 | .055 | -2.742 | .032 |
| SUPPORT | 1 | 2 | 2.211^*^ | .823 | 170.291 | .008 | .586 | 3.835 |
|  |  | 3 | 3.829^*^ | .845 | 173.974 | <.001 | 2.162 | 5.495 |
|  | 2 | 1 | -2.211^*^ | .823 | 170.291 | .008 | -3.835 | -.586 |
|  |  | 3 | 1.618 | .873 | 164.960 | .066 | -.105 | 3.341 |
|  | 3 | 1 | -3.829^*^ | .845 | 173.974 | <.001 | -5.495 | -2.162 |
|  |  | 2 | -1.618 | .873 | 164.960 | .066 | -3.341 | .105 |
| EXPECT | 1 | 2 | 1.122 | .595 | 163.682 | .061 | -.053 | 2.296 |
|  |  | 3 | 3.431^*^ | .607 | 165.120 | <.001 | 2.232 | 4.631 |
|  | 2 | 1 | -1.122 | .595 | 163.682 | .061 | -2.296 | .053 |
|  |  | 3 | 2.310^*^ | .611 | 165.807 | <.001 | 1.103 | 3.516 |
|  | 3 | 1 | -3.431^*^ | .607 | 165.120 | <.001 | -4.631 | -2.232 |
|  |  | 2 | -2.310^*^ | .611 | 165.807 | <.001 | -3.516 | -1.103 |
| Notes: Dependent Variable: HADS_Depressive symptoms. Based on estimated marginal means. Time 1 = T1 (1 day pre-surgery); time 2 = T2 (1 week post-surgery), time 3 = T3 (6 months follow-up-assessment). *. The mean difference is significant at the .05 level. | | | | | | | | |

| **Supplementary table 3. Post-hoc group comparisons for T1, T2 and T3 personal control and tests for change over time within each intervention group.**   \| *Estimates for rate of personal control* (low (-1 SD)) \| \| \| \| \| \| \| \| --- \| --- \| --- \| --- \| --- \| --- \| --- \| \| Intervention group \| time \| Mean \| Std. Error \| df \| 95% Confidence Interval \| \| \| Lower Bound \| Upper Bound \| \| SMC \| 1 \| 2.785 \| 2.152 \| .000 \| -19.348 \| 24.917 \| \| 2 \| 4.785 \| 2.158 \| .000 \| -32.114 \| 41.684 \| \|  \| 3 \| 3.646 \| 2.176 \| .000 \| -11.695 \| 18.998 \| \| SUPPORT \| 1 \| 2.823 \| 2.127 \| .000 \| -7.947 \| 13.594 \| \| 2 \| 5.698 \| 2.141 \| .000 \| -4.227 \| 15.622 \| \|  \| 3 \| 4.530 \| 2.156 \| .000 \| -54.638 \| 63.698 \| \| EXPECT \| 1 \| 6.079 \| 2.118 \| .000 \| -4.483 \| 16.642 \| \| 2 \| 5.871 \| 2.110 \| .000 \| -4.184 \| 15.926 \| \|  \| 3 \| 5.152 \| 2.158 \| .000 \| -33.098 \| 43.401 \| \| Notes: Dependent Variable: B-IPQ_Personal control. Time 1 = 1 day pre-surgery; time 2 = 1 week post-surgery; time 3 = 6 months follow-up-assessment. Adjusted score T0 (baseline) = 1.403. \| \| \| \| \| \| \| \|  \| \| \| \| \| \| \|  \| *Pairwise Comparisons for rate of personal control at T1, T2 and T3* (low (-1 SD)) \| \| \| \| \| \| \| \| \| \| --- \| --- \| --- \| --- \| --- \| --- \| --- \| --- \| --- \| \| time \| (I) Intervention group \| (J) Intervention group \| Mean Difference (I-J) \| Std. Error \| df \| Sig. \| 95% Confidence Interval for Difference \| \| \| Lower Bound \| Upper Bound \| \| 1 \| SMC \| SUPPORT \| -.039 \| .848 \| 95.395 \| .964 \| -1.721 \| 1.644 \| \| EXPECT \| -3.295^*^ \| .825 \| 95.397 \| <.001 \| -4.932 \| -1.658 \| \| SUPPORT \| SMC \| .039 \| .848 \| 95.395 \| .964 \| -1.644 \| 1.721 \| \| EXPECT \| -3.256^*^ \| .758 \| 95.396 \| <.001 \| -4.760 \| -1.752 \| \| EXPECT \| SMC \| 3.295^*^ \| .825 \| 95.397 \| <.001 \| 1.658 \| 4.932 \| \| SUPPORT \| 3.256^*^ \| .758 \| 95.396 \| <.001 \| 1.752 \| 4.760 \| \| 2 \| SMC \| SUPPORT \| -.912 \| .896 \| 88.219 \| .311 \| -2.693 \| .868 \| \| EXPECT \| -1.086 \| .820 \| 88.138 \| .189 \| -2.716 \| .544 \| \| SUPPORT \| SMC \| .912 \| .896 \| 88.219 \| .311 \| -.868 \| 2.693 \| \| EXPECT \| -.174 \| .774 \| 88.196 \| .823 \| -1.713 \| 1.365 \| \| EXPECT \| SMC \| 1.086 \| .820 \| 88.138 \| .189 \| -.544 \| 2.716 \| \| SUPPORT \| .174 \| .774 \| 88.196 \| .823 \| -1.365 \| 1.713 \| \| 3 \| SMC \| SUPPORT \| -.884 \| .971 \| 81.699 \| .366 \| -2.816 \| 1.049 \| \| EXPECT \| -1.505 \| .976 \| 81.789 \| .127 \| -3.446 \| .436 \| \| SUPPORT \| SMC \| .884 \| .971 \| 81.699 \| .366 \| -1.049 \| 2.816 \| \| EXPECT \| -.621 \| .931 \| 81.827 \| .506 \| -2.473 \| 1.230 \| \| EXPECT \| SMC \| 1.505 \| .976 \| 81.789 \| .127 \| -.436 \| 3.446 \| \|  \|  \| SUPPORT \| .621 \| .931 \| 81.827 \| .506 \| -1.230 \| 2.473 \| \| Notes: Dependent Variable: B-IPQ_Personal control. Based on estimated marginal means. Time 1 = T1 (1 day pre-surgery); time 2 = T2 (1 week post-surgery), time 3 = T3 (6 months follow-up-assessment). *. The mean difference is significant at the .05 level. \| \| \| \| \| \| \| \| \|  \| *Pairwise Comparisons: change over time* *for rate of personal control* (low (-1 SD)) \| \| \| \| \| \| \| \| \| \| --- \| --- \| --- \| --- \| --- \| --- \| --- \| --- \| --- \| \| Intervention group \| (I) time \| (J) time \| Mean Difference (I-J) \| Std. Error \| df \| Sig. \| 95% Confidence Interval for Difference^c^ \| \| \| Lower Bound \| Upper Bound \| \| SMC \| 1 \| 2 \| -2.000^*^ \| .891 \| 122.911 \| .027 \| -3.765 \| -.236 \| \|  \| 3 \| -.862 \| .960 \| 171.009 \| .371 \| -2.758 \| 1.034 \| \|  \| 2 \| 1 \| 2.000^*^ \| .891 \| 122.911 \| .027 \| .236 \| 3.765 \| \|  \|  \| 3 \| 1.139 \| .940 \| 114.057 \| .228 \| -.724 \| 3.002 \| \|  \| 3 \| 1 \| .862 \| .960 \| 171.009 \| .371 \| -1.034 \| 2.758 \| \|  \|  \| 2 \| -1.139 \| .940 \| 114.057 \| .228 \| -3.002 \| .724 \| \| SUPPORT \| 1 \| 2 \| -2.874^*^ \| .792 \| 122.585 \| <.001 \| -4.442 \| -1.306 \| \|  \| 3 \| -1.707^*^ \| .855 \| 167.094 \| .048 \| -3.395 \| -.019 \| \|  \| 2 \| 1 \| 2.874^*^ \| .792 \| 122.585 \| <.001 \| 1.306 \| 4.442 \| \|  \|  \| 3 \| 1.167 \| .862 \| 121.555 \| .178 \| -.539 \| 2.873 \| \|  \| 3 \| 1 \| 1.707^*^ \| .855 \| 167.094 \| .048 \| .019 \| 3.395 \| \|  \|  \| 2 \| -1.167 \| .862 \| 121.555 \| .178 \| -2.873 \| .539 \| \| EXPECT \| 1 \| 2 \| .208 \| .684 \| 118.661 \| .761 \| -1.146 \| 1.562 \| \|  \| 3 \| .928 \| .838 \| 160.744 \| .270 \| -.727 \| 2.583 \| \|  \| 2 \| 1 \| -.208 \| .684 \| 118.661 \| .761 \| -1.562 \| 1.146 \| \|  \|  \| 3 \| .720 \| .798 \| 123.595 \| .369 \| -.859 \| 2.298 \| \|  \| 3 \| 1 \| -.928 \| .838 \| 160.744 \| .270 \| -2.583 \| .727 \| \|  \|  \| 2 \| -.720 \| .798 \| 123.595 \| .369 \| -2.298 \| .859 \| \| Notes: Dependent Variable: B-IPQ_Personal control. Based on estimated marginal means. Time 1 = T1 (1 day pre-surgery); time 2 = T2 (1 week post-surgery), time 3 = T3 (6 months follow-up-assessment). *. The mean difference is significant at the .05 level. \| \| \| \| \| \| \| \| \|  \| *Estimates for rate of personal control* (average (mean)) \| \| \| \| \| \| \| \| --- \| --- \| --- \| --- \| --- \| --- \| --- \| \| Intervention group \| time \| Mean \| Std. Error \| df \| 95% Confidence Interval \| \| \| Lower Bound \| Upper Bound \| \| SMC \| 1 \| 3.825 \| 2.090 \| .000 \| -6.212 \| 13.862 \| \| 2 \| 5.447 \| 2.089 \| .000 \| -4.791 \| 15.685 \| \|  \| 3 \| 4.323 \| 2.097 \| .000 \| -4.349 \| 12.994 \| \| SUPPORT \| 1 \| 4.961 \| 2.093 \| .000 \| -4.342 \| 14.264 \| \| 2 \| 6.052 \| 2.098 \| .000 \| -4.486 \| 16.589 \| \|  \| 3 \| 5.019 \| 2.103 \| .000 \| -4.404 \| 14.442 \| \| EXPECT \| 1 \| 5.843 \| 2.094 \| .000 \| -3.384 \| 15.069 \| \| 2 \| 6.221 \| 2.090 \| .000 \| -3.833 \| 16.275 \| \|  \| 3 \| 4.714 \| 2.112 \| .000 \| -5.028 \| 14.457 \| \| Notes: Dependent Variable: B-IPQ_Personal control. Time 1 = 1 day pre-surgery; time 2 = 1 week post-surgery; time 3 = 6 months follow-up-assessment. Adjusted score T0 (baseline) = 4.41. \| \| \| \| \| \| \|  \| *Pairwise Comparisons for rate of personal control at T1, T2 and T3* (average (mean)) \| \| \| \| \| \| \| \| \| \| --- \| --- \| --- \| --- \| --- \| --- \| --- \| --- \| --- \| \| time \| (I) Intervention group \| (J) Intervention group \| Mean Difference (I-J) \| Std. Error \| df \| Sig. \| 95% Confidence Interval for Difference \| \| \| Lower Bound \| Upper Bound \| \| 1 \| SMC \| SUPPORT \| -1.136^*^ \| .558 \| 95.396 \| .045 \| -2.244 \| -.028 \| \| EXPECT \| -2.018^*^ \| .559 \| 95.397 \| <.001 \| -3.128 \| -.907 \| \| SUPPORT \| SMC \| 1.136^*^ \| .558 \| 95.396 \| .045 \| .028 \| 2.244 \| \| EXPECT \| -.882 \| .572 \| 95.397 \| .127 \| -2.018 \| .254 \| \| EXPECT \| SMC \| 2.018^*^ \| .559 \| 95.397 \| <.001 \| .907 \| 3.128 \| \| SUPPORT \| .882 \| .572 \| 95.397 \| .127 \| -.254 \| 2.018 \| \| 2 \| SMC \| SUPPORT \| -.604 \| .572 \| 88.215 \| .294 \| -1.741 \| .532 \| \| EXPECT \| -.774 \| .541 \| 88.122 \| .156 \| -1.849 \| .301 \| \| SUPPORT \| SMC \| .604 \| .572 \| 88.215 \| .294 \| -.532 \| 1.741 \| \| EXPECT \| -.170 \| .575 \| 88.204 \| .769 \| -1.312 \| .973 \| \| EXPECT \| SMC \| .774 \| .541 \| 88.122 \| .156 \| -.301 \| 1.849 \| \| SUPPORT \| .170 \| .575 \| 88.204 \| .769 \| -.973 \| 1.312 \| \| 3 \| SMC \| SUPPORT \| -.696 \| .617 \| 81.708 \| .263 \| -1.924 \| .531 \| \| EXPECT \| -.392 \| .646 \| 81.799 \| .546 \| -1.677 \| .893 \| \| SUPPORT \| SMC \| .696 \| .617 \| 81.708 \| .263 \| -.531 \| 1.924 \| \| EXPECT \| .304 \| .667 \| 81.795 \| .650 \| -1.023 \| 1.631 \| \| EXPECT \| SMC \| .392 \| .646 \| 81.799 \| .546 \| -.893 \| 1.677 \| \|  \|  \| SUPPORT \| -.304 \| .667 \| 81.795 \| .650 \| -1.631 \| 1.023 \| \| Notes: Dependent Variable: B-IPQ_Personal control. Based on estimated marginal means. Time 1 = T1 (1 day pre-surgery); time 2 = T2 (1 week post-surgery), time 3 = T3 (6 months follow-up-assessment). *. The mean difference is significant at the .05 level. \| \| \| \| \| \| \| \| \|  \| *Pairwise Comparisons: change over time* *for rate of personal control* (average (mean)) \| \| \| \| \| \| \| \| \| \| --- \| --- \| --- \| --- \| --- \| --- \| --- \| --- \| --- \| \| Intervention group \| (I) time \| (J) time \| Mean Difference (I-J) \| Std. Error \| df \| Sig. \| 95% Confidence Interval for Difference^c^ \| \| \| Lower Bound \| Upper Bound \| \| SMC \| 1 \| 2 \| -1.622^*^ \| .523 \| 120.555 \| .002 \| -2.657 \| -.588 \| \|  \| 3 \| -.498 \| .569 \| 172.302 \| .383 \| -1.620 \| .625 \| \|  \| 2 \| 1 \| 1.622^*^ \| .523 \| 120.555 \| .002 \| .588 \| 2.657 \| \|  \|  \| 3 \| 1.125^*^ \| .547 \| 116.730 \| .042 \| .042 \| 2.207 \| \|  \| 3 \| 1 \| .498 \| .569 \| 172.302 \| .383 \| -.625 \| 1.620 \| \|  \|  \| 2 \| -1.125^*^ \| .547 \| 116.730 \| .042 \| -2.207 \| -.042 \| \| SUPPORT \| 1 \| 2 \| -1.091 \| .569 \| 125.894 \| .057 \| -2.217 \| .035 \| \|  \| 3 \| -.058 \| .604 \| 170.860 \| .924 \| -1.251 \| 1.135 \| \|  \| 2 \| 1 \| 1.091 \| .569 \| 125.894 \| .057 \| -.035 \| 2.217 \| \|  \|  \| 3 \| 1.033 \| .601 \| 120.886 \| .088 \| -.157 \| 2.223 \| \|  \| 3 \| 1 \| .058 \| .604 \| 170.860 \| .924 \| -1.135 \| 1.251 \| \|  \|  \| 2 \| -1.033 \| .601 \| 120.886 \| .088 \| -2.223 \| .157 \| \| EXPECT \| 1 \| 2 \| -.379 \| .540 \| 122.395 \| .485 \| -1.448 \| .691 \| \|  \| 3 \| 1.128 \| .635 \| 165.324 \| .078 \| -.126 \| 2.382 \| \|  \| 2 \| 1 \| .379 \| .540 \| 122.395 \| .485 \| -.691 \| 1.448 \| \|  \|  \| 3 \| 1.507^*^ \| .605 \| 122.702 \| .014 \| .309 \| 2.705 \| \|  \| 3 \| 1 \| -1.128 \| .635 \| 165.324 \| .078 \| -2.382 \| .126 \| \|  \|  \| 2 \| -1.507^*^ \| .605 \| 122.702 \| .014 \| -2.705 \| -.309 \| \| Notes: Dependent Variable: B-IPQ_Personal control. Based on estimated marginal means. Time 1 = T1 (1 day pre-surgery); time 2 = T2 (1 week post-surgery), time 3 = T3 (6 months follow-up-assessment). *. The mean difference is significant at the .05 level. \| \| \| \| \| \| \| \| \| |
| --- | --- | --- | --- | --- | --- | --- | --- | --- | --- | --- | --- | --- | --- | --- | --- | --- | --- | --- | --- | --- | --- | --- | --- | --- | --- | --- | --- | --- | --- | --- | --- | --- | --- | --- | --- | --- | --- | --- | --- | --- | --- | --- | --- | --- | --- | --- | --- | --- | --- | --- | --- | --- | --- | --- | --- | --- | --- | --- | --- | --- | --- | --- | --- | --- | --- | --- | --- | --- | --- | --- | --- | --- | --- | --- | --- | --- | --- | --- | --- | --- | --- | --- | --- | --- | --- | --- | --- | --- | --- | --- | --- | --- | --- | --- | --- | --- | --- | --- | --- | --- | --- | --- | --- | --- | --- | --- | --- | --- | --- | --- | --- | --- | --- | --- | --- | --- | --- | --- | --- | --- | --- | --- | --- | --- | --- | --- | --- | --- | --- | --- | --- | --- | --- | --- | --- | --- | --- | --- | --- | --- | --- | --- | --- | --- | --- | --- | --- | --- | --- | --- | --- | --- | --- | --- | --- | --- | --- | --- | --- | --- | --- | --- | --- | --- | --- | --- | --- | --- | --- | --- | --- | --- | --- | --- | --- | --- | --- | --- | --- | --- | --- | --- | --- | --- | --- | --- | --- | --- | --- | --- | --- | --- | --- | --- | --- | --- | --- | --- | --- | --- | --- | --- | --- | --- | --- | --- | --- | --- | --- | --- | --- | --- | --- | --- | --- | --- | --- | --- | --- | --- | --- | --- | --- | --- | --- | --- | --- | --- | --- | --- | --- | --- | --- | --- | --- | --- | --- | --- | --- | --- | --- | --- | --- | --- | --- | --- | --- | --- | --- | --- | --- | --- | --- | --- | --- | --- | --- | --- | --- | --- | --- | --- | --- | --- | --- | --- | --- | --- | --- | --- | --- | --- | --- | --- | --- | --- | --- | --- | --- | --- | --- | --- | --- | --- | --- | --- | --- | --- | --- | --- | --- | --- | --- | --- | --- | --- | --- | --- | --- | --- | --- | --- | --- | --- | --- | --- | --- | --- | --- | --- | --- | --- | --- | --- | --- | --- | --- | --- | --- | --- | --- | --- | --- | --- | --- | --- | --- | --- | --- | --- | --- | --- | --- | --- | --- | --- | --- | --- | --- | --- | --- | --- | --- | --- | --- | --- | --- | --- | --- | --- | --- | --- | --- | --- | --- | --- | --- | --- | --- | --- | --- | --- | --- | --- | --- | --- | --- | --- | --- | --- | --- | --- | --- | --- | --- | --- | --- | --- | --- | --- | --- | --- | --- | --- | --- | --- | --- | --- | --- | --- | --- | --- | --- | --- | --- | --- | --- | --- | --- | --- | --- | --- | --- | --- | --- | --- | --- | --- | --- | --- | --- | --- | --- | --- | --- | --- | --- | --- | --- | --- | --- | --- | --- | --- | --- | --- | --- | --- | --- | --- | --- | --- | --- | --- | --- | --- | --- | --- | --- | --- | --- | --- | --- | --- | --- | --- | --- | --- | --- | --- | --- | --- | --- | --- | --- | --- | --- | --- | --- | --- | --- | --- | --- | --- | --- | --- | --- | --- | --- | --- | --- | --- | --- | --- | --- | --- | --- | --- | --- | --- | --- | --- | --- | --- | --- | --- | --- | --- | --- | --- | --- | --- | --- | --- | --- | --- | --- | --- | --- | --- | --- | --- | --- | --- | --- | --- | --- | --- | --- | --- | --- | --- | --- | --- | --- | --- | --- | --- | --- | --- | --- | --- | --- | --- | --- | --- | --- | --- | --- | --- | --- | --- | --- | --- | --- | --- | --- | --- | --- | --- | --- | --- | --- | --- | --- | --- | --- | --- | --- | --- | --- | --- | --- | --- | --- | --- | --- | --- | --- | --- | --- | --- | --- | --- | --- | --- | --- | --- | --- | --- | --- | --- | --- | --- | --- | --- | --- | --- | --- | --- | --- | --- | --- | --- | --- | --- | --- | --- | --- | --- | --- | --- | --- | --- | --- | --- | --- | --- | --- | --- | --- | --- | --- | --- | --- | --- | --- | --- | --- | --- | --- | --- | --- | --- | --- | --- | --- | --- | --- | --- | --- | --- | --- | --- | --- | --- | --- | --- | --- | --- | --- | --- | --- | --- | --- | --- | --- | --- | --- | --- | --- | --- | --- | --- | --- | --- | --- | --- | --- | --- | --- | --- | --- | --- | --- | --- | --- | --- | --- | --- | --- | --- | --- | --- | --- | --- | --- | --- | --- | --- | --- | --- | --- | --- | --- | --- | --- | --- | --- | --- | --- | --- | --- | --- | --- | --- | --- | --- | --- | --- | --- | --- | --- | --- | --- | --- | --- | --- | --- | --- | --- | --- | --- | --- | --- | --- | --- | --- | --- | --- | --- | --- | --- | --- | --- | --- | --- | --- | --- | --- | --- | --- | --- | --- | --- | --- | --- | --- | --- | --- | --- | --- | --- | --- | --- | --- | --- | --- | --- | --- | --- | --- | --- | --- | --- | --- | --- | --- | --- | --- | --- | --- | --- | --- | --- | --- | --- | --- | --- | --- | --- | --- | --- | --- | --- | --- | --- | --- | --- | --- | --- | --- | --- | --- | --- | --- | --- | --- | --- | --- | --- | --- | --- | --- | --- | --- | --- | --- | --- | --- | --- | --- | --- | --- | --- | --- | --- | --- | --- | --- | --- | --- | --- | --- | --- | --- | --- | --- | --- | --- | --- | --- | --- | --- | --- | --- | --- | --- | --- | --- | --- | --- | --- | --- | --- | --- | --- | --- | --- | --- | --- | --- | --- | --- | --- | --- | --- | --- | --- | --- | --- | --- | --- | --- | --- | --- | --- | --- | --- | --- | --- | --- | --- | --- | --- | --- | --- | --- | --- | --- | --- | --- | --- | --- | --- | --- | --- | --- | --- | --- | --- | --- | --- | --- | --- | --- | --- | --- | --- | --- | --- | --- | --- | --- | --- | --- | --- |

| *Estimates for rate of personal control* (high (+ 1SD)) | | | | | | |
| --- | --- | --- | --- | --- | --- | --- |
| Intervention group | time | Mean | Std. Error | df | 95% Confidence Interval | |
|  |  |  |  |  | Lower Bound | Upper Bound |
| SMC | 1 | 4.769 | 2.119 | .000 | -5.651 | 15.188 |
|  | 2 | 6.048 | 2.117 | .000 | -4.673 | 16.769 |
|  | 3 | 4.936 | 2.136 | .000 | -6.124 | 15.996 |
| SUPPORT | 1 | 6.900 | 2.123 | .000 | -2.712 | 16.511 |
|  | 2 | 6.373 | 2.125 | .000 | -2.885 | 15.601 |
|  | 3 | 5.462 | 2.132 | .000 | -4.208 | 15.132 |
| EXPECT | 1 | 6.628 | 2.141 | .000 | -4.287 | 15.542 |
|  | 2 | 6.538 | 2.138 | .000 | -4.095 | 17.172 |
|  | 3 | 4.318 | 2.183 | .000 | -24.215 | 32.851 |
| Notes: Dependent Variable: B-IPQ_Personal control. Time 1 = 1 day pre-surgery; time 2 = 1 week post-surgery; time 3 = 6 months follow-up-assessment. Adjusted score T0 (baseline) = 7.137. | | | | | | |
|  | | | | | | |

| *Pairwise Comparisons for rate of personal control at T1, T2 and T3* (high (+1 SD)) | | | | | | | | |
| --- | --- | --- | --- | --- | --- | --- | --- | --- |
| time | (I) Intervention group | (J) Intervention group | Mean Difference (I-J) | Std. Error | df | Sig. | 95% Confidence Interval for Difference | |
|  |  |  |  |  |  |  | Lower Bound | Upper Bound |
| 1 | SMC | SUPPORT | -2.131^*^ | .746 | 95.396 | .005 | -3.613 | -.650 |
|  |  | EXPECT | -.859 | .797 | 95.397 | .284 | -2.442 | .724 |
|  | SUPPORT | SMC | 2.131^*^ | .746 | 95.396 | .005 | .650 | 3.613 |
|  |  | EXPECT | 1.272 | .807 | 95.397 | .118 | -.331 | 2.875 |
|  | EXPECT | SMC | .859 | .797 | 95.397 | .284 | -.724 | 2.442 |
|  |  | SUPPORT | -1.272 | .807 | 95.397 | .118 | -2.875 | .331 |
| 2 | SMC | SUPPORT | -.325 | .748 | 88.180 | .665 | -1.812 | 1.161 |
|  |  | EXPECT | -.491 | .785 | 88.139 | .533 | -2.050 | 1.068 |
|  | SUPPORT | SMC | .325 | .748 | 88.180 | .665 | -1.161 | 1.812 |
|  |  | EXPECT | -.166 | .804 | 88.195 | .837 | -1.763 | 1.431 |
|  | EXPECT | SMC | .491 | .785 | 88.139 | .533 | -1.068 | 2.050 |
|  |  | SUPPORT | .166 | .804 | 88.195 | .837 | -1.431 | 1.763 |
| 3 | SMC | SUPPORT | -.526 | .819 | 81.723 | .523 | -2.155 | 1.104 |
|  |  | EXPECT | .618 | .944 | 81.813 | .515 | -1.260 | 2.496 |
|  | SUPPORT | SMC | .526 | .819 | 81.723 | .523 | -1.104 | 2.155 |
|  |  | EXPECT | 1.144 | .936 | 81.798 | .225 | -.718 | 3.006 |
|  | EXPECT | SMC | -.618 | .944 | 81.813 | .515 | -2.496 | 1.260 |
|  |  | SUPPORT | -1.144 | .936 | 81.798 | .225 | -3.006 | .718 |
| Notes: Dependent Variable: B-IPQ_Personal control. Based on estimated marginal means. Time 1 = T1 (1 day pre-surgery); time 2 = T2 (1 week post-surgery), time 3 = T3 (6 months follow-up-assessment). *. The mean difference is significant at the .05 level. | | | | | | | | |

| *Pairwise Comparisons: change over time* *for rate of personal control* (low (-1 SD)) | | | | | | | | |
| --- | --- | --- | --- | --- | --- | --- | --- | --- |
| Intervention group | (I) time | (J) time | Mean Difference (I-J) | Std. Error | df | Sig. | 95% Confidence Interval for Difference^c^ | |
|  |  |  |  |  |  |  | Lower Bound | Upper Bound |
| SMC | 1 | 2 | -1.279 | .705 | 116.744 | .072 | -2.675 | .117 |
|  |  | 3 | -.167 | .781 | 170.399 | .831 | -1.709 | 1.374 |
|  | 2 | 1 | 1.279 | .705 | 116.744 | .072 | -.117 | 2.675 |
|  |  | 3 | 1.112 | .753 | 118.838 | .143 | -.379 | 2.603 |
|  | 3 | 1 | .167 | .781 | 170.399 | .831 | -1.374 | 1.709 |
|  |  | 2 | -1.112 | .753 | 118.838 | .143 | -2.603 | .379 |
| SUPPORT | 1 | 2 | .527 | .738 | 125.372 | .477 | -.933 | 1.987 |
|  |  | 3 | 1.438 | .782 | 173.277 | .068 | -.106 | 2.981 |
|  | 2 | 1 | -.527 | .738 | 125.372 | .477 | -1.987 | .933 |
|  |  | 3 | .911 | .762 | 119.694 | .234 | -.598 | 2.420 |
|  | 3 | 1 | -1.438 | .782 | 173.277 | .068 | -2.981 | .106 |
|  |  | 2 | -.911 | .762 | 119.694 | .234 | -2.420 | .598 |
| EXPECT | 1 | 2 | -.911 | .818 | 123.943 | .268 | -2.530 | .708 |
|  |  | 3 | 1.310 | .954 | 164.379 | .171 | -.573 | 3.192 |
|  | 2 | 1 | .911 | .818 | 123.943 | .268 | -.708 | 2.530 |
|  |  | 3 | 2.220^*^ | .920 | 124.571 | .017 | .399 | 4.042 |
|  | 3 | 1 | -1.310 | .954 | 164.379 | .171 | -3.192 | .573 |
|  |  | 2 | -2.220^*^ | .920 | 124.571 | .017 | -4.042 | -.399 |
| Notes: Dependent Variable: B-IPQ_Personal control. Based on estimated marginal means. Time 1 = T1 (1 day pre-surgery); time 2 = T2 (1 week post-surgery), time 3 = T3 (6 months follow-up-assessment). *. The mean difference is significant at the .05 level. | | | | | | | | |

| **Supplementary table 4. Post-hoc group comparisons for T1, T2 and T3 concern and tests for change over time within each intervention group.**   \| *Estimates for rate of concern* (low (-1 SD)) \| \| \| \| \| \| \| \| --- \| --- \| --- \| --- \| --- \| --- \| --- \| \| Intervention group \| time \| Mean \| Std. Error \| df \| 95% Confidence Interval \| \| \| Lower Bound \| Upper Bound \| \| SMC \| 1 \| 3.322 \| 1.084 \| 1399.878 \| 1.196 \| 5.447 \| \| 2 \| 4.65 \| 1.136 \| 896.763 \| 2.421 \| 6.879 \| \|  \| 3 \| 2.736 \| 1.083 \| 1371.926 \| .612 \| 4.860 \| \| SUPPORT \| 1 \| 3.256 \| 1.125 \| 964.750 \| 1.048 \| 5.464 \| \| 2 \| 3.257 \| 1.228 \| 542.595 \| .846 \| 5.669 \| \|  \| 3 \| 1.56 \| 1.186 \| 644.117 \| -.769 \| 3.889 \| \| EXPECT \| 1 \| 4.882 \| 1.069 \| 1651.302 \| 2.786 \| 6.979 \| \| 2 \| 3.398 \| 1.098 \| 1188.733 \| 1.244 \| 5.551 \| \|  \| 3 \| 1.645 \| 1.075 \| 1502.690 \| -.462 \| 3.753 \| \| Notes: Dependent Variable: B-IPQ_Concern. Time 1 = 1 day pre-surgery; time 2 = 1 week post-surgery; time 3 = 6 months follow-up-assessment. Adjusted score T0 (baseline) = 2.762. \| \| \| \| \| \| \|  \| *Pairwise Comparisons for rate of concern at T1, T2 and T3* (low (-1 SD)) \| \| \| \| \| \| \| \| \| \| --- \| --- \| --- \| --- \| --- \| --- \| --- \| --- \| --- \| \| time \| (I) Intervention group \| (J) Intervention group \| Mean Difference (I-J) \| Std. Error \| df \| Sig. \| 95% Confidence Interval for Difference \| \| \| Lower Bound \| Upper Bound \| \| 1 \| SMC \| SUPPORT \| .066 \| .845 \| 98.111 \| .938 \| -1.612 \| 1.743 \| \| EXPECT \| -1.561^*^ \| .769 \| 98.605 \| .045 \| -3.086 \| -.036 \| \| SUPPORT \| SMC \| -.066 \| .845 \| 98.111 \| .938 \| -1.743 \| 1.612 \| \| EXPECT \| -1.626 \| .826 \| 98.214 \| .052 \| -3.266 \| .013 \| \| EXPECT \| SMC \| 1.561^*^ \| .769 \| 98.605 \| .045 \| .036 \| 3.086 \| \| SUPPORT \| 1.626 \| .826 \| 98.214 \| .052 \| -.013 \| 3.266 \| \| 2 \| SMC \| SUPPORT \| 1.393 \| 1.035 \| 98.801 \| .181 \| -.661 \| 3.446 \| \| EXPECT \| 1.252 \| .877 \| 97.153 \| .156 \| -.488 \| 2.992 \| \| SUPPORT \| SMC \| -1.393 \| 1.035 \| 98.801 \| .181 \| -3.446 \| .661 \| \| EXPECT \| -.140 \| .993 \| 98.050 \| .888 \| -2.111 \| 1.830 \| \| EXPECT \| SMC \| -1.252 \| .877 \| 97.153 \| .156 \| -2.992 \| .488 \| \| SUPPORT \| .140 \| .993 \| 98.050 \| .888 \| -1.830 \| 2.111 \| \| 3 \| SMC \| SUPPORT \| 1.176 \| .924 \| 96.131 \| .206 \| -.658 \| 3.010 \| \| EXPECT \| 1.090 \| .775 \| 95.778 \| .163 \| -.449 \| 2.630 \| \| SUPPORT \| SMC \| -1.176 \| .924 \| 96.131 \| .206 \| -3.010 \| .658 \| \| EXPECT \| -.086 \| .915 \| 96.288 \| .926 \| -1.901 \| 1.730 \| \| EXPECT \| SMC \| -1.090 \| .775 \| 95.778 \| .163 \| -2.630 \| .449 \| \|  \|  \| SUPPORT \| .086 \| .915 \| 96.288 \| .926 \| -1.730 \| 1.901 \| \| Notes: Dependent Variable: B-IPQ_Concern. Based on estimated marginal means. Time 1 = T1 (1 day pre-surgery); time 2 = T2 (1 week post-surgery), time 3 = T3 (6 months follow-up-assessment). *. The mean difference is significant at the .05 level. \| \| \| \| \| \| \| \| \|  \| *Pairwise Comparisons: change over time* *for rate of concern* (low (-1 SD)) \| \| \| \| \| \| \| \| \| \| --- \| --- \| --- \| --- \| --- \| --- \| --- \| --- \| --- \| \| Intervention group \| (I) time \| (J) time \| Mean Difference (I-J) \| Std. Error \| df \| Sig. \| 95% Confidence Interval for Difference^c^ \| \| \| Lower Bound \| Upper Bound \| \| SMC \| 1 \| 2 \| -1.328 \| .734 \| 139.802 \| .073 \| -2.780 \| .124 \| \|  \| 3 \| .586 \| .754 \| 187.003 \| .438 \| -.902 \| 2.073 \| \|  \| 2 \| 1 \| 1.328 \| .734 \| 139.802 \| .073 \| -.124 \| 2.780 \| \|  \|  \| 3 \| 1.914^*^ \| .731 \| 134.758 \| .010 \| .468 \| 3.360 \| \|  \| 3 \| 1 \| -.586 \| .754 \| 187.003 \| .438 \| -2.073 \| .902 \| \|  \|  \| 2 \| -1.914^*^ \| .731 \| 134.758 \| .010 \| -3.360 \| -.468 \| \| SUPPORT \| 1 \| 2 \| -.001 \| .878 \| 137.821 \| .999 \| -1.737 \| 1.734 \| \|  \| 3 \| 1.696 \| .935 \| 182.878 \| .071 \| -.150 \| 3.542 \| \|  \| 2 \| 1 \| .001 \| .878 \| 137.821 \| .999 \| -1.734 \| 1.737 \| \|  \|  \| 3 \| 1.697 \| .940 \| 139.785 \| .073 \| -.160 \| 3.555 \| \|  \| 3 \| 1 \| -1.696 \| .935 \| 182.878 \| .071 \| -3.542 \| .150 \| \|  \|  \| 2 \| -1.697 \| .940 \| 139.785 \| .073 \| -3.555 \| .160 \| \| EXPECT \| 1 \| 2 \| 1.485^*^ \| .669 \| 141.043 \| .028 \| .163 \| 2.807 \| \|  \| 3 \| 3.237^*^ \| .725 \| 186.773 \| <.001 \| 1.807 \| 4.667 \| \|  \| 2 \| 1 \| -1.485^*^ \| .669 \| 141.043 \| .028 \| -2.807 \| -.163 \| \|  \|  \| 3 \| 1.752^*^ \| .675 \| 136.544 \| .010 \| .418 \| 3.087 \| \|  \| 3 \| 1 \| -3.237^*^ \| .725 \| 186.773 \| <.001 \| -4.667 \| -1.807 \| \|  \|  \| 2 \| -1.752^*^ \| .675 \| 136.544 \| .010 \| -3.087 \| -.418 \| \| Notes: Dependent Variable: B-IPQ_Concern. Based on estimated marginal means. Time 1 = T1 (1 day pre-surgery); time 2 = T2 (1 week post-surgery), time 3 = T3 (6 months follow-up-assessment). *. The mean difference is significant at the .05 level. \| \| \| \| \| \| \| \| \|  \| *Estimates for rate of concern* (average (mean)) \| \| \| \| \| \| \| \| --- \| --- \| --- \| --- \| --- \| --- \| --- \| \| Intervention group \| time \| Mean \| Std. Error \| df \| 95% Confidence Interval \| \| \| Lower Bound \| Upper Bound \| \| SMC \| 1 \| 5.926 \| 1.007 \| 4412.779 \| 3.951 \| 7.900 \| \| 2 \| 5.740 \| 1.035 \| 2591.965 \| 3.711 \| 7.769 \| \|  \| 3 \| 3.395 \| 1.013 \| 3819.875 \| 1.410 \| 5.380 \| \| SUPPORT \| 1 \| 5.705 \| 1.015 \| 3750.542 \| 3.716 \| 7.694 \| \| 2 \| 4.623 \| 1.053 \| 1999.222 \| 2.558 \| 6.689 \| \|  \| 3 \| 3.229 \| 1.027 \| 2903.001 \| 1.216 \| 5.242 \| \| EXPECT \| 1 \| 6.056 \| 1.011 \| 4050.819 \| 4.074 \| 8.038 \| \| 2 \| 4.745 \| 1.034 \| 2596.100 \| 2.719 \| 6.772 \| \|  \| 3 \| 3.145 \| 1.016 \| 3556.438 \| 1.153 \| 5.137 \| \| Notes: Dependent Variable: B-IPQ_Concern. Time 1 = 1 day pre-surgery; time 2 = 1 week post-surgery; time 3 = 6 months follow-up-assessment. Adjusted score T0 (baseline) = 6.15. \| \| \| \| \| \| \|  \| *Pairwise Comparisons for rate of concern at T1, T2 and T3* (average (mean)) \| \| \| \| \| \| \| \| \| \| --- \| --- \| --- \| --- \| --- \| --- \| --- \| --- \| --- \| \| time \| (I) Intervention group \| (J) Intervention group \| Mean Difference (I-J) \| Std. Error \| df \| Sig. \| 95% Confidence Interval for Difference \| \| \| Lower Bound \| Upper Bound \| \| 1 \| SMC \| SUPPORT \| .221 \| .564 \| 98.213 \| .697 \| -.898 \| 1.339 \| \| EXPECT \| -.130 \| .557 \| 98.344 \| .816 \| -1.236 \| .976 \| \| SUPPORT \| SMC \| -.221 \| .564 \| 98.213 \| .697 \| -1.339 \| .898 \| \| EXPECT \| -.351 \| .571 \| 98.331 \| .540 \| -1.484 \| .782 \| \| EXPECT \| SMC \| .130 \| .557 \| 98.344 \| .816 \| -.976 \| 1.236 \| \| SUPPORT \| .351 \| .571 \| 98.331 \| .540 \| -.782 \| 1.484 \| \| 2 \| SMC \| SUPPORT \| 1.116 \| .674 \| 98.236 \| .101 \| -.221 \| 2.454 \| \| EXPECT \| .995 \| .643 \| 96.960 \| .125 \| -.282 \| 2.271 \| \| SUPPORT \| SMC \| -1.116 \| .674 \| 98.236 \| .101 \| -2.454 \| .221 \| \| EXPECT \| -.122 \| .672 \| 97.456 \| .856 \| -1.456 \| 1.212 \| \| EXPECT \| SMC \| -.995 \| .643 \| 96.960 \| .125 \| -2.271 \| .282 \| \| SUPPORT \| .122 \| .672 \| 97.456 \| .856 \| -1.212 \| 1.456 \| \| 3 \| SMC \| SUPPORT \| .166 \| .595 \| 95.950 \| .781 \| -1.015 \| 1.348 \| \| EXPECT \| .251 \| .576 \| 95.908 \| .665 \| -.893 \| 1.394 \| \| SUPPORT \| SMC \| -.166 \| .595 \| 95.950 \| .781 \| -1.348 \| 1.015 \| \| EXPECT \| .084 \| .601 \| 95.855 \| .889 \| -1.108 \| 1.277 \| \| EXPECT \| SMC \| -.251 \| .576 \| 95.908 \| .665 \| -1.394 \| .893 \| \|  \|  \| SUPPORT \| -.084 \| .601 \| 95.855 \| .889 \| -1.277 \| 1.108 \| \| Notes: Dependent Variable: B-IPQ_Concern. Based on estimated marginal means. Time 1 = T1 (1 day pre-surgery); time 2 = T2 (1 week post-surgery), time 3 = T3 (6 months follow-up-assessment). *. The mean difference is significant at the .05 level. \| \| \| \| \| \| \| \| \|  \| *Pairwise Comparisons: change over time* *for rate of concern* (average (mean)) \| \| \| \| \| \| \| \| \| \| --- \| --- \| --- \| --- \| --- \| --- \| --- \| --- \| --- \| \| Intervention group \| (I) time \| (J) time \| Mean Difference (I-J) \| Std. Error \| df \| Sig. \| 95% Confidence Interval for Difference^c^ \| \| \| Lower Bound \| Upper Bound \| \| SMC \| 1 \| 2 \| .186 \| .509 \| 137.446 \| .716 \| -.821 \| 1.193 \| \|  \| 3 \| 2.530^*^ \| .537 \| 186.912 \| <.001 \| 1.471 \| 3.590 \| \|  \| 2 \| 1 \| -.186 \| .509 \| 137.446 \| .716 \| -1.193 \| .821 \| \|  \|  \| 3 \| 2.345^*^ \| .522 \| 139.340 \| <.001 \| 1.313 \| 3.376 \| \|  \| 3 \| 1 \| -2.530^*^ \| .537 \| 186.912 \| <.001 \| -3.590 \| -1.471 \| \|  \|  \| 2 \| -2.345^*^ \| .522 \| 139.340 \| <.001 \| -3.376 \| -1.313 \| \| SUPPORT \| 1 \| 2 \| 1.082 \| .550 \| 139.902 \| .051 \| -.006 \| 2.170 \| \|  \| 3 \| 2.476^*^ \| .574 \| 186.590 \| <.001 \| 1.344 \| 3.609 \| \|  \| 2 \| 1 \| -1.082 \| .550 \| 139.902 \| .051 \| -2.170 \| .006 \| \|  \|  \| 3 \| 1.394^*^ \| .565 \| 135.861 \| .015 \| .277 \| 2.511 \| \|  \| 3 \| 1 \| -2.476^*^ \| .574 \| 186.590 \| <.001 \| -3.609 \| -1.344 \| \|  \|  \| 2 \| -1.394^*^ \| .565 \| 135.861 \| .015 \| -2.511 \| -.277 \| \| EXPECT \| 1 \| 2 \| 1.311^*^ \| .512 \| 140.255 \| .012 \| .298 \| 2.323 \| \|  \| 3 \| 2.911^*^ \| .550 \| 186.992 \| <.001 \| 1.827 \| 3.995 \| \|  \| 2 \| 1 \| -1.311^*^ \| .512 \| 140.255 \| .012 \| -2.323 \| -.298 \| \|  \|  \| 3 \| 1.601^*^ \| .517 \| 134.690 \| .002 \| .577 \| 2.624 \| \|  \| 3 \| 1 \| -2.911^*^ \| .550 \| 186.992 \| <.001 \| -3.995 \| -1.827 \| \|  \|  \| 2 \| -1.601^*^ \| .517 \| 134.690 \| .002 \| -2.624 \| -.577 \| \| Notes: Dependent Variable: B-IPQ_Concern. Based on estimated marginal means. Time 1 = T1 (1 day pre-surgery); time 2 = T2 (1 week post-surgery), time 3 = T3 (6 months follow-up-assessment). *. The mean difference is significant at the .05 level. \| \| \| \| \| \| \| \| \| |
| --- | --- | --- | --- | --- | --- | --- | --- | --- | --- | --- | --- | --- | --- | --- | --- | --- | --- | --- | --- | --- | --- | --- | --- | --- | --- | --- | --- | --- | --- | --- | --- | --- | --- | --- | --- | --- | --- | --- | --- | --- | --- | --- | --- | --- | --- | --- | --- | --- | --- | --- | --- | --- | --- | --- | --- | --- | --- | --- | --- | --- | --- | --- | --- | --- | --- | --- | --- | --- | --- | --- | --- | --- | --- | --- | --- | --- | --- | --- | --- | --- | --- | --- | --- | --- | --- | --- | --- | --- | --- | --- | --- | --- | --- | --- | --- | --- | --- | --- | --- | --- | --- | --- | --- | --- | --- | --- | --- | --- | --- | --- | --- | --- | --- | --- | --- | --- | --- | --- | --- | --- | --- | --- | --- | --- | --- | --- | --- | --- | --- | --- | --- | --- | --- | --- | --- | --- | --- | --- | --- | --- | --- | --- | --- | --- | --- | --- | --- | --- | --- | --- | --- | --- | --- | --- | --- | --- | --- | --- | --- | --- | --- | --- | --- | --- | --- | --- | --- | --- | --- | --- | --- | --- | --- | --- | --- | --- | --- | --- | --- | --- | --- | --- | --- | --- | --- | --- | --- | --- | --- | --- | --- | --- | --- | --- | --- | --- | --- | --- | --- | --- | --- | --- | --- | --- | --- | --- | --- | --- | --- | --- | --- | --- | --- | --- | --- | --- | --- | --- | --- | --- | --- | --- | --- | --- | --- | --- | --- | --- | --- | --- | --- | --- | --- | --- | --- | --- | --- | --- | --- | --- | --- | --- | --- | --- | --- | --- | --- | --- | --- | --- | --- | --- | --- | --- | --- | --- | --- | --- | --- | --- | --- | --- | --- | --- | --- | --- | --- | --- | --- | --- | --- | --- | --- | --- | --- | --- | --- | --- | --- | --- | --- | --- | --- | --- | --- | --- | --- | --- | --- | --- | --- | --- | --- | --- | --- | --- | --- | --- | --- | --- | --- | --- | --- | --- | --- | --- | --- | --- | --- | --- | --- | --- | --- | --- | --- | --- | --- | --- | --- | --- | --- | --- | --- | --- | --- | --- | --- | --- | --- | --- | --- | --- | --- | --- | --- | --- | --- | --- | --- | --- | --- | --- | --- | --- | --- | --- | --- | --- | --- | --- | --- | --- | --- | --- | --- | --- | --- | --- | --- | --- | --- | --- | --- | --- | --- | --- | --- | --- | --- | --- | --- | --- | --- | --- | --- | --- | --- | --- | --- | --- | --- | --- | --- | --- | --- | --- | --- | --- | --- | --- | --- | --- | --- | --- | --- | --- | --- | --- | --- | --- | --- | --- | --- | --- | --- | --- | --- | --- | --- | --- | --- | --- | --- | --- | --- | --- | --- | --- | --- | --- | --- | --- | --- | --- | --- | --- | --- | --- | --- | --- | --- | --- | --- | --- | --- | --- | --- | --- | --- | --- | --- | --- | --- | --- | --- | --- | --- | --- | --- | --- | --- | --- | --- | --- | --- | --- | --- | --- | --- | --- | --- | --- | --- | --- | --- | --- | --- | --- | --- | --- | --- | --- | --- | --- | --- | --- | --- | --- | --- | --- | --- | --- | --- | --- | --- | --- | --- | --- | --- | --- | --- | --- | --- | --- | --- | --- | --- | --- | --- | --- | --- | --- | --- | --- | --- | --- | --- | --- | --- | --- | --- | --- | --- | --- | --- | --- | --- | --- | --- | --- | --- | --- | --- | --- | --- | --- | --- | --- | --- | --- | --- | --- | --- | --- | --- | --- | --- | --- | --- | --- | --- | --- | --- | --- | --- | --- | --- | --- | --- | --- | --- | --- | --- | --- | --- | --- | --- | --- | --- | --- | --- | --- | --- | --- | --- | --- | --- | --- | --- | --- | --- | --- | --- | --- | --- | --- | --- | --- | --- | --- | --- | --- | --- | --- | --- | --- | --- | --- | --- | --- | --- | --- | --- | --- | --- | --- | --- | --- | --- | --- | --- | --- | --- | --- | --- | --- | --- | --- | --- | --- | --- | --- | --- | --- | --- | --- | --- | --- | --- | --- | --- | --- | --- | --- | --- | --- | --- | --- | --- | --- | --- | --- | --- | --- | --- | --- | --- | --- | --- | --- | --- | --- | --- | --- | --- | --- | --- | --- | --- | --- | --- | --- | --- | --- | --- | --- | --- | --- | --- | --- | --- | --- | --- | --- | --- | --- | --- | --- | --- | --- | --- | --- | --- | --- | --- | --- | --- | --- | --- | --- | --- | --- | --- | --- | --- | --- | --- | --- | --- | --- | --- | --- | --- | --- | --- | --- | --- | --- | --- | --- | --- | --- | --- | --- | --- | --- | --- | --- | --- | --- | --- | --- | --- | --- | --- | --- | --- | --- | --- | --- | --- | --- | --- | --- | --- | --- | --- | --- | --- | --- | --- | --- | --- | --- | --- | --- | --- | --- | --- | --- | --- | --- | --- | --- | --- | --- | --- | --- | --- | --- | --- | --- | --- | --- | --- | --- | --- | --- | --- | --- | --- | --- | --- | --- | --- | --- | --- | --- | --- | --- | --- | --- | --- | --- | --- | --- | --- | --- | --- | --- | --- | --- | --- | --- | --- | --- | --- | --- | --- | --- | --- | --- | --- | --- | --- | --- | --- | --- | --- | --- | --- | --- | --- | --- | --- | --- | --- | --- | --- | --- | --- | --- | --- | --- | --- | --- | --- | --- | --- | --- | --- | --- | --- | --- | --- | --- | --- | --- | --- | --- | --- | --- | --- | --- | --- | --- | --- | --- | --- | --- | --- | --- | --- | --- | --- | --- | --- | --- | --- | --- | --- | --- | --- | --- | --- | --- | --- | --- | --- | --- | --- | --- | --- | --- | --- | --- | --- | --- | --- | --- | --- | --- | --- | --- | --- | --- | --- | --- | --- | --- |

| *Estimates for rate of concern* (high (+ 1SD)) | | | | | | |
| --- | --- | --- | --- | --- | --- | --- |
| Intervention group | time | Mean | Std. Error | df | 95% Confidence Interval | |
|  |  |  |  |  | Lower Bound | Upper Bound |
| SMC | 1 | 8.415 | 1.101 | 1182.856 | 6.256 | 10.574 |
|  | 2 | 6.782 | 1.155 | 771.884 | 4.514 | 9.050 |
|  | 3 | 4.025 | 1.123 | 966.708 | 1.822 | 6.228 |
| SUPPORT | 1 | 8.047 | 1.078 | 1487.376 | 5.933 | 10.161 |
|  | 2 | 5.930 | 1.119 | 994.395 | 3.734 | 8.125 |
|  | 3 | 4.825 | 1.085 | 1342.428 | 2.696 | 6.953 |
| EXPECT | 1 | 7.178 | 1.080 | 1457.251 | 5.060 | 9.296 |
|  | 2 | 6.034 | 1.122 | 971.414 | 3.832 | 8.235 |
|  | 3 | 4.578 | 1.086 | 1326.749 | 2.448 | 6.708 |
| Notes: Dependent Variable: B-IPQ_Concern. Time 1 = 1 day pre-surgery; time 2 = 1 week post-surgery; time 3 = 6 months follow-up-assessment. Adjusted score T0 (baseline) = 9.398 | | | | | | |

| *Pairwise Comparisons for rate of concern at T1, T2 and T3* (high (+1 SD)) | | | | | | | | |
| --- | --- | --- | --- | --- | --- | --- | --- | --- |
| time | (I) Intervention group | (J) Intervention group | Mean Difference (I-J) | Std. Error | df | Sig. | 95% Confidence Interval for Difference | |
|  |  |  |  |  |  |  | Lower Bound | Upper Bound |
| 1 | SMC | SUPPORT | .369 | .804 | 98.121 | .648 | -1.228 | 1.965 |
|  |  | EXPECT | 1.237 | .807 | 98.100 | .128 | -.364 | 2.839 |
|  | SUPPORT | SMC | -.369 | .804 | 98.121 | .648 | -1.965 | 1.228 |
|  |  | EXPECT | .869 | .775 | 98.323 | .265 | -.670 | 2.407 |
|  | EXPECT | SMC | -1.237 | .807 | 98.100 | .128 | -2.839 | .364 |
|  |  | SUPPORT | -.869 | .775 | 98.323 | .265 | -2.407 | .670 |
| 2 | SMC | SUPPORT | .852 | .928 | 96.291 | .361 | -.989 | 2.694 |
|  |  | EXPECT | .748 | .931 | 96.239 | .424 | -1.100 | 2.597 |
|  | SUPPORT | SMC | -.852 | .928 | 96.291 | .361 | -2.694 | .989 |
|  |  | EXPECT | -.104 | .886 | 96.011 | .907 | -1.862 | 1.654 |
|  | EXPECT | SMC | -.748 | .931 | 96.239 | .424 | -2.597 | 1.100 |
|  |  | SUPPORT | .104 | .886 | 96.011 | .907 | -1.654 | 1.862 |
| 3 | SMC | SUPPORT | -.799 | .844 | 95.962 | .346 | -2.474 | .875 |
|  |  | EXPECT | -.552 | .845 | 95.922 | .515 | -2.230 | 1.125 |
|  | SUPPORT | SMC | .799 | .844 | 95.962 | .346 | -.875 | 2.474 |
|  |  | EXPECT | .247 | .794 | 95.639 | .757 | -1.330 | 1.823 |
|  | EXPECT | SMC | .552 | .845 | 95.922 | .515 | -1.125 | 2.230 |
|  |  | SUPPORT | -.247 | .794 | 95.639 | .757 | -1.823 | 1.330 |
| Notes: Dependent Variable: B-IPQ_Concern. Based on estimated marginal means. Time 1 = T1 (1 day pre-surgery); time 2 = T2 (1 week post-surgery), time 3 = T3 (6 months follow-up-assessment). *. The mean difference is significant at the .05 level. | | | | | | | | |

| *Pairwise Comparisons: change over time* *for rate of concern* (high (+1 SD)) | | | | | | | | |
| --- | --- | --- | --- | --- | --- | --- | --- | --- |
| Intervention group | (I) time | (J) time | Mean Difference (I-J) | Std. Error | df | Sig. | 95% Confidence Interval for Difference^c^ | |
|  |  |  |  |  |  |  | Lower Bound | Upper Bound |
| SMC | 1 | 2 | 1.633^*^ | .763 | 135.466 | .034 | .124 | 3.142 |
|  |  | 3 | 4.390^*^ | .828 | 185.965 | <.001 | 2.756 | 6.023 |
|  | 2 | 1 | -1.633^*^ | .763 | 135.466 | .034 | -3.142 | -.124 |
|  |  | 3 | 2.756^*^ | .796 | 139.632 | .001 | 1.182 | 4.331 |
|  | 3 | 1 | -4.390^*^ | .828 | 185.965 | <.001 | -6.023 | -2.756 |
|  |  | 2 | -2.756^*^ | .796 | 139.632 | .001 | -4.331 | -1.182 |
| SUPPORT | 1 | 2 | 2.117^*^ | .702 | 139.383 | .003 | .729 | 3.506 |
|  |  | 3 | 3.222^*^ | .750 | 186.966 | <.001 | 1.743 | 4.701 |
|  | 2 | 1 | -2.117^*^ | .702 | 139.383 | .003 | -3.506 | -.729 |
|  |  | 3 | 1.105 | .708 | 134.108 | .121 | -.295 | 2.505 |
|  | 3 | 1 | -3.222^*^ | .750 | 186.966 | <.001 | -4.701 | -1.743 |
|  |  | 2 | -1.105 | .708 | 134.108 | .121 | -2.505 | .295 |
| EXPECT | 1 | 2 | 1.144 | .707 | 139.117 | .108 | -.253 | 2.542 |
|  |  | 3 | 2.600^*^ | .753 | 187.062 | .001 | 1.114 | 4.086 |
|  | 2 | 1 | -1.144 | .707 | 139.117 | .108 | -2.542 | .253 |
|  |  | 3 | 1.456^*^ | .711 | 133.484 | .043 | .049 | 2.862 |
|  | 3 | 1 | -2.600^*^ | .753 | 187.062 | .001 | -4.086 | -1.114 |
|  |  | 2 | -1.456^*^ | .711 | 133.484 | .043 | -2.862 | -.049 |
| Notes: Dependent Variable: B-IPQ_Concern. Based on estimated marginal means. Time 1 = T1 (1 day pre-surgery); time 2 = T2 (1 week post-surgery), time 3 = T3 (6 months follow-up-assessment). *. The mean difference is significant at the .05 level. | | | | | | | | |

**Supplementary table 5. Additional analyses.**

| *Correlations between Depressive symptoms and physical (rehospitalization, EF) outcomes* | | | | | | |
| --- | --- | --- | --- | --- | --- | --- |
|  | | HADS Depressive symptoms T1 | HADS Depressive symptoms T2 | HADS Depressive symptoms T3 | complications that lead  to rehospitalization T3 | EF T3 |
| HADS Depressive symptoms T1 | Pearson Correlation | -- |  |  |  |  |
|  | N | 101 |  |  |  |  |
| HADS Depressive symptoms T2 | Pearson Correlation | .429^**^ | -- |  |  |  |
|  | Sig. (2-tailed) | .000 |  |  |  |  |
|  | N | 88 | 99 |  |  |  |
| HADS Depressive symptoms T3 | Pearson Correlation | .580^**^ | .394^**^ | -- |  |  |
|  | Sig. (2-tailed) | .000 | .000 |  |  |  |
|  | N | 86 | 87 | 95 |  |  |
| complications that lead to rehospitalization T3 | Pearson Correlation | .290^**^ | .175 | .214^*^ | -- |  |
|  | Sig. (2-tailed) | .007 | .102 | .044 |  |  |
|  | N | 85 | 88 | 89 | 96 |  |
| EF T3 | Pearson Correlation | .030 | -.039 | -.019 | -.093 | -- |
|  | Sig. (2-tailed) | .805 | .744 | .870 | .431 |  |
|  | N | 72 | 73 | 73 | 74 | 81 |
| Notes: EF = Ejection fraction (pump function of the heart); T1 = 1 day pre-surgery, T2 = 1 week post-surgery, T3 = 6 months follow-up-assessment. *. The mean difference is significant at the .05 level. **. Correlation is significant at the .01 level. | | | | | | |

| *Correlations between Anxiety and physical (rehospitalization, EF) outcomes* | | | | | | |
| --- | --- | --- | --- | --- | --- | --- |
|  | | HADS Anxiety T1 | HADS Anxiety T2 | HADS Anxiety T3 | complications that lead  to rehospitalization T3 | EF T3 |
| HADS Anxiety T1 | Pearson Correlation | -- |  |  |  |  |
|  | N | 100 |  |  |  |  |
| HADS Anxiety T2 | Pearson Correlation | .574^**^ | -- |  |  |  |
|  | Sig. (2-tailed) | .000 |  |  |  |  |
|  | N | 86 | 98 |  |  |  |
| HADS Anxiety T3 | Pearson Correlation | .613^**^ | .454^**^ | -- |  |  |
|  | Sig. (2-tailed) | .000 | .000 |  |  |  |
|  | N | 89 | 89 | 100 |  |  |
| complications that lead to rehospitalization T3 | Pearson Correlation | .239^*^ | .230^*^ | .032 | -- |  |
|  | Sig. (2-tailed) | .028 | .032 | .758 |  |  |
|  | N | 84 | 87 | 94 | 96 |  |
| EF T3 | Pearson Correlation | -.094 | -.042 | -.025 | -.093 | -- |
|  | Sig. (2-tailed) | .434 | .723 | .826 | .431 |  |
|  | N | 71 | 72 | 77 | 74 | 81 |
| Notes: EF = Ejection fraction (pump function of the heart); T1 = 1 day pre-surgery, T2 = 1 week post-surgery, T3 = 6 months follow-up-assessment. *. The mean difference is significant at the .05 level. **. Correlation is significant at the .01 level. | | | | | | |

| *Correlations between Sumscore and physical (rehospitalization, EF) outcomes* | | | | | | |
| --- | --- | --- | --- | --- | --- | --- |
|  | | HADS Sumscore T1 | HADS Sumscore T2 | HADS Sumscore T3 | complications that lead  to rehospitalization T3 | EF T3 |
| HADS Sumscore T1 | Pearson Correlation | -- |  |  |  |  |
|  | N | 100 |  |  |  |  |
| HADS Sumscore T2 | Pearson Correlation | .583^**^ | -- |  |  |  |
|  | Sig. (2-tailed) | .000 |  |  |  |  |
|  | N | 85 | 97 |  |  |  |
| HADS Sumscore T3 | Pearson Correlation | .668^**^ | .531^**^ | -- |  |  |
|  | Sig. (2-tailed) | .000 | .000 |  |  |  |
|  | N | 85 | 86 | 95 |  |  |
| complications that lead to rehospitalization T3 | Pearson Correlation | .272^*^ | .257^*^ | .155 | -- |  |
|  | Sig. (2-tailed) | .012 | .017 | .146 |  |  |
|  | N | 84 | 86 | 89 | 96 |  |
| EF T3 | Pearson Correlation | -.037 | -.057 | -.046 | -.093 | -- |
|  | Sig. (2-tailed) | .759 | .634 | .700 | .431 |  |
|  | N | 71 | 71 | 73 | 74 | 81 |
| Notes: EF = Ejection fraction (pump function of the heart); T1 = 1 day pre-surgery, T2 = 1 week post-surgery, T3 = 6 months follow-up-assessment. *. The mean difference is significant at the .05 level. **. Correlation is significant at the .01 level. | | | | | | |

| *Correlations between Consequences and physical (rehospitalization, EF) outcomes* | | | | | | |
| --- | --- | --- | --- | --- | --- | --- |
|  | | B-IPQ Consequences T1 | B-IPQ Consequences T2 | B-IPQ Consequences T3 | complications that lead  to rehospitalization T3 | EF T3 |
| B-IPQ Consequences T1 | Pearson Correlation | -- |  |  |  |  |
|  | N | 104 |  |  |  |  |
| B-IPQ Consequences T2 | Pearson Correlation | .272^**^ | -- |  |  |  |
|  | Sig. (2-tailed) | .009 |  |  |  |  |
|  | N | 91 | 99 |  |  |  |
| B-IPQ Consequences T3 | Pearson Correlation | .261^*^ | .202 | -- |  |  |
|  | Sig. (2-tailed) | .012 | .056 |  |  |  |
|  | N | 92 | 90 | 100 |  |  |
| complications that lead to rehospitalization T3 | Pearson Correlation | .208 | .092 | .289^**^ | -- |  |
|  | Sig. (2-tailed) | .052 | .394 | .005 |  |  |
|  | N | 88 | 88 | 94 | 96 |  |
| EF T3 | Pearson Correlation | .125 | -.028 | -.046 | -.093 | -- |
|  | Sig. (2-tailed) | .289 | .810 | .691 | .431 |  |
|  | N | 74 | 74 | 78 | 74 | 81 |
| Notes: EF = Ejection fraction (pump function of the heart); T1 = 1 day pre-surgery, T2 = 1 week post-surgery, T3 = 6 months follow-up-assessment. *. The mean difference is significant at the .05 level. **. Correlation is significant at the .01 level. | | | | | | |

| *Correlations between Timeline and physical (rehospitalization, EF) outcomes* | | | | | | |
| --- | --- | --- | --- | --- | --- | --- |
|  | | B-IPQ Timeline T1 | B-IPQ Timeline T2 | B-IPQ Timeline T3 | complications that lead  to rehospitalization T3 | EF T3 |
| B-IPQ Timeline T1 | Pearson Correlation | -- |  |  |  |  |
|  | N | 99 |  |  |  |  |
| B-IPQ Timeline T2 | Pearson Correlation | .384^**^ | -- |  |  |  |
|  | Sig. (2-tailed) | .000 |  |  |  |  |
|  | N | 89 | 102 |  |  |  |
| B-IPQ Timeline T3 | Pearson Correlation | .403^**^ | .302^**^ | -- |  |  |
|  | Sig. (2-tailed) | .000 | .004 |  |  |  |
|  | N | 85 | 91 | 98 |  |  |
| complications that lead to rehospitalization T3 | Pearson Correlation | .104 | -.003 | .286^**^ | -- |  |
|  | Sig. (2-tailed) | .350 | .978 | .006 |  |  |
|  | N | 83 | 92 | 92 | 96 |  |
| EF T3 | Pearson Correlation | .184 | -.056 | -.022 | -.093 | -- |
|  | Sig. (2-tailed) | .130 | .632 | .850 | .431 |  |
|  | N | 69 | 76 | 77 | 74 | 81 |
| Notes: EF = Ejection fraction (pump function of the heart); T1 = 1 day pre-surgery, T2 = 1 week post-surgery, T3 = 6 months follow-up-assessment. *. The mean difference is significant at the .05 level. **. Correlation is significant at the .01 level. | | | | | | |

| *Correlations between Personal control and physical (rehospitalization, EF) outcomes* | | | | | | |
| --- | --- | --- | --- | --- | --- | --- |
|  | | B-IPQ Personal control T1 | B-IPQ Personal control T2 | B-IPQ Personal control T3 | complications that lead  to rehospitalization T3 | EF T3 |
| B-IPQ Personal control T1 | Pearson Correlation | -- |  |  |  |  |
|  | N | 104 |  |  |  |  |
| B-IPQ Personal control T2 | Pearson Correlation | .173 | -- |  |  |  |
|  | Sig. (2-tailed) | .108 |  |  |  |  |
|  | N | 88 | 96 |  |  |  |
| B-IPQ Personal control T3 | Pearson Correlation | .134 | .027 | -- |  |  |
|  | Sig. (2-tailed) | .223 | .813 |  |  |  |
|  | N | 85 | 80 | 91 |  |  |
| complications that lead to rehospitalization T3 | Pearson Correlation | .053 | -.045 | .036 | -- |  |
|  | Sig. (2-tailed) | .626 | .681 | .742 |  |  |
|  | N | 88 | 86 | 85 | 96 |  |
| EF T3 | Pearson Correlation | -.104 | .142 | .063 | -.093 | -- |
|  | Sig. (2-tailed) | .373 | .236 | .606 | .431 |  |
|  | N | 75 | 71 | 69 | 74 | 81 |
| Notes: EF = Ejection fraction (pump function of the heart); T1 = 1 day pre-surgery, T2 = 1 week post-surgery, T3 = 6 months follow-up-assessment. *. The mean difference is significant at the .05 level. **. Correlation is significant at the .01 level. | | | | | | |

| *Correlations between Treatment control and physical (rehospitalization, EF) outcomes* | | | | | | |
| --- | --- | --- | --- | --- | --- | --- |
|  | | B-IPQ Treatment control T1 | B-IPQ Treatment control T2 | B-IPQ Treatment control T3 | complications that lead  to rehospitalization T3 | EF T3 |
| B-IPQ Treatment control T1 | Pearson Correlation | -- |  |  |  |  |
|  | N | 97 |  |  |  |  |
| B-IPQ Treatment control T2 | Pearson Correlation | .373^**^ | -- |  |  |  |
|  | Sig. (2-tailed) | .000 |  |  |  |  |
|  | N | 91 | 103 |  |  |  |
| B-IPQ Treatment control T3 | Pearson Correlation | .157 | .396^**^ | -- |  |  |
|  | Sig. (2-tailed) | .154 | .000 |  |  |  |
|  | N | 84 | 91 | 96 |  |  |
| complications that lead to rehospitalization T3 | Pearson Correlation | -.033 | .018 | -.293^**^ | -- |  |
|  | Sig. (2-tailed) | .767 | .863 | .005 |  |  |
|  | N | 85 | 92 | 90 | 96 |  |
| EF T3 | Pearson Correlation | -.107 | .089 | .169 | -.093 | -- |
|  | Sig. (2-tailed) | .377 | .442 | .149 | .431 |  |
|  | N | 70 | 77 | 74 | 74 | 81 |
| Notes: EF = Ejection fraction (pump function of the heart); T1 = 1 day pre-surgery, T2 = 1 week post-surgery, T3 = 6 months follow-up-assessment. *. The mean difference is significant at the .05 level. **. Correlation is significant at the .01 level. | | | | | | |

| *Correlations between Identity and physical (rehospitalization, EF) outcomes* | | | | | | |
| --- | --- | --- | --- | --- | --- | --- |
|  | | B-IPQ Identity T1 | B-IPQ Identity T2 | B-IPQ Identity T3 | complications that lead  to rehospitalization T3 | EF T3 |
| B-IPQ Identity T1 | Pearson Correlation | -- |  |  |  |  |
|  | N | 104 |  |  |  |  |
| B-IPQ Identity T2 | Pearson Correlation | .116 | -- |  |  |  |
|  | Sig. (2-tailed) | .266 |  |  |  |  |
|  | N | 94 | 101 |  |  |  |
| B-IPQ Identity T3 | Pearson Correlation | .213^*^ | .260^*^ | -- |  |  |
|  | Sig. (2-tailed) | .042 | .013 |  |  |  |
|  | N | 91 | 91 | 99 |  |  |
| complications that lead to rehospitalization T3 | Pearson Correlation | .162 | .036 | .352^**^ | -- |  |
|  | Sig. (2-tailed) | .130 | .734 | .001 |  |  |
|  | N | 89 | 90 | 93 | 96 |  |
| EF T3 | Pearson Correlation | .194 | -.042 | -.091 | -.093 | -- |
|  | Sig. (2-tailed) | .098 | .718 | .435 | .431 |  |
|  | N | 74 | 75 | 76 | 74 | 81 |
| Notes: EF = Ejection fraction (pump function of the heart); T1 = 1 day pre-surgery, T2 = 1 week post-surgery, T3 = 6 months follow-up-assessment. *. The mean difference is significant at the .05 level. **. Correlation is significant at the .01 level. | | | | | | |

| *Correlations between Concern and physical (rehospitalization, EF) outcomes* | | | | | | |
| --- | --- | --- | --- | --- | --- | --- |
|  | | B-IPQ Concern T1 | B-IPQ Concern T2 | B-IPQ Concern T3 | complications that lead  to rehospitalization T3 | EF T3 |
| B-IPQ Concern T1 | Pearson Correlation | -- |  |  |  |  |
|  | N | 104 |  |  |  |  |
| B-IPQ Concern T2 | Pearson Correlation | .453^**^ | -- |  |  |  |
|  | Sig. (2-tailed) | .000 |  |  |  |  |
|  | N | 95 | 102 |  |  |  |
| B-IPQ Concern T3 | Pearson Correlation | .299^**^ | .485^**^ | -- |  |  |
|  | Sig. (2-tailed) | .004 | .000 |  |  |  |
|  | N | 92 | 93 | 100 |  |  |
| complications that lead to rehospitalization T3 | Pearson Correlation | .152 | .129 | .269^**^ | -- |  |
|  | Sig. (2-tailed) | .155 | .224 | .008 |  |  |
|  | N | 89 | 91 | 95 | 96 |  |
| EF T3 | Pearson Correlation | .125 | .054 | .014 | -.093 | -- |
|  | Sig. (2-tailed) | .289 | .643 | .904 | .431 |  |
|  | N | 74 | 77 | 77 | 74 | 81 |
| Notes: EF = Ejection fraction (pump function of the heart); T1 = 1 day pre-surgery, T2 = 1 week post-surgery, T3 = 6 months follow-up-assessment. *. The mean difference is significant at the .05 level. **. Correlation is significant at the .01 level. | | | | | | |

| *Correlations between Understanding and physical (rehospitalization, EF) outcomes* | | | | | | |
| --- | --- | --- | --- | --- | --- | --- |
|  | | B-IPQ Understanding T1 | B-IPQ Understanding T2 | B-IPQ Understanding T3 | complications that lead  to rehospitalization T3 | EF T3 |
| B-IPQ Understanding T1 | Pearson Correlation | -- |  |  |  |  |
|  | N | 100 |  |  |  |  |
| B-IPQ Understanding T2 | Pearson Correlation | .551^**^ | -- |  |  |  |
|  | Sig. (2-tailed) | .000 |  |  |  |  |
|  | N | 91 | 101 |  |  |  |
| B-IPQ Understanding T3 | Pearson Correlation | .403^**^ | .402^**^ | -- |  |  |
|  | Sig. (2-tailed) | .000 | .000 |  |  |  |
|  | N | 85 | 89 | 96 |  |  |
| complications that lead to rehospitalization T3 | Pearson Correlation | -.059 | -.037 | -.099 | -- |  |
|  | Sig. (2-tailed) | .594 | .728 | .352 |  |  |
|  | N | 85 | 90 | 90 | 96 |  |
| EF T3 | Pearson Correlation | .097 | -.047 | -.004 | -.093 | -- |
|  | Sig. (2-tailed) | .423 | .690 | .976 | .431 |  |
|  | N | 71 | 76 | 74 | 74 | 81 |
| Notes: EF = Ejection fraction (pump function of the heart); T1 = 1 day pre-surgery, T2 = 1 week post-surgery, T3 = 6 months follow-up-assessment. *. The mean difference is significant at the .05 level. **. Correlation is significant at the .01 level. | | | | | | |

| *Correlations between Emotional response and physical (rehospitalization, EF) outcomes* | | | | | | |
| --- | --- | --- | --- | --- | --- | --- |
|  | | B-IPQ Emotional response T1 | B-IPQ Emotional response T2 | B-IPQ Emotional response T3 | complications that lead  to rehospitalization T3 | EF T3 |
| B-IPQ Emotional response T1 | Pearson Correlation | -- |  |  |  |  |
|  | N | 105 |  |  |  |  |
| B-IPQ Emotional response T2 | Pearson Correlation | .431^**^ | -- |  |  |  |
|  | Sig. (2-tailed) | .000 |  |  |  |  |
|  | N | 96 | 103 |  |  |  |
| B-IPQ Emotional response T3 | Pearson Correlation | .337^**^ | .323^**^ | -- |  |  |
|  | Sig. (2-tailed) | .001 | .002 |  |  |  |
|  | N | 93 | 94 | 100 |  |  |
| complications that lead to rehospitalization T3 | Pearson Correlation | .253^*^ | .240^*^ | .242^*^ | -- |  |
|  | Sig. (2-tailed) | .017 | .021 | .019 |  |  |
|  | N | 89 | 92 | 94 | 96 |  |
| EF T3 | Pearson Correlation | -.079 | -.137 | .056 | -.093 | -- |
|  | Sig. (2-tailed) | .503 | .234 | .628 | .431 |  |
|  | N | 75 | 77 | 77 | 74 | 81 |
| Notes: EF = Ejection fraction (pump function of the heart); T1 = 1 day pre-surgery, T2 = 1 week post-surgery, T3 = 6 months follow-up-assessment. *. The mean difference is significant at the .05 level. **. Correlation is significant at the .01 level. | | | | | | |

**Supplementary materials. Results of the analyses.**

**Mixed Model Analysis – Results for depressive symptoms**

| *Model Dimension* | | | | | | |
| --- | --- | --- | --- | --- | --- | --- |
|  | | Number of Levels | Covariance Structure | Number of Parameters | Subject Variables | Number of Subjects |
| Fixed Effects | Intercept | 1 |  | 1 |  |  |
|  | Group | 3 |  | 2 |  |  |
|  | time | 3 |  | 2 |  |  |
|  | Group * time | 9 |  | 4 |  |  |
|  | HADS_Depressive symptoms_T0 | 1 |  | 1 |  |  |
|  | Group * HADS_Depressive symptoms_T0 | 3 |  | 2 |  |  |
|  | time * HADS_Depressive symptoms_T0 | 3 |  | 2 |  |  |
|  | Group * time * HADS_Depressive symptoms_T0 | 9 |  | 4 |  |  |
| Random Effects | Intercept | 1 | Variance Components | 1 |  |  |
| Repeated Effects | time | 3 | Compound Symmetry | 2 | id | 106 |
| Total | | 36 |  | 21 |  |  |
| Notes: Dependent Variable: HADS_Depressive symptoms. | | | | | | |

| *Type III Tests of Fixed Effects* | | | | |
| --- | --- | --- | --- | --- |
| Source | Numerator df | Denominator df | F | Sig. |
| Intercept | 1 | .000 | 5.337 | 1.000 |
| Group | 2 | 84.205 | .764 | .469 |
| time | 2 | 163.869 | 8.030 | <.001 |
| Group * time | 4 | 163.827 | 1.075 | .371 |
| HADS_Depressive symptoms_T0 | 1 | 82.632 | 91.032 | <.001 |
| Group * HADS_Depressive symptoms_T0 | 2 | 82.694 | .322 | .726 |
| time * HADS_Depressive symptoms_T0 | 2 | 162.148 | 10.134 | <.001 |
| Group * time * HADS_Depressive symptoms_T0 | 4 | 162.184 | 2.569 | .040 |
| Notes: Dependent Variable: HADS_Depressive symptoms. | | | | |

| *Estimates of Fixed Effects* | | | | | | | |
| --- | --- | --- | --- | --- | --- | --- | --- |
| Parameter | Estimate | Std. Error | df | t | Sig. | 95% Confidence Interval | |
|  |  |  |  |  |  | Lower Bound | Upper Bound |
| Intercept | .809323 | .980594 | .000 | .825 | 1.000 | -6.281526 | 7.900172 |
| [Group=1] | -.011725 | 1.033280 | 244.836 | -.011 | .991 | -2.046976 | 2.023527 |
| [Group=2] | .416174 | 1.064450 | 249.886 | .391 | .696 | -1.680264 | 2.512611 |
| [Group=3] | 0^a^ | 0 |  |  |  |  |  |
| [time=1] | .199696 | .943179 | 176.209 | .212 | .833 | -1.661683 | 2.061076 |
| [time=2] | 2.055143 | .880307 | 162.386 | 2.335 | .021 | .316817 | 3.793469 |
| [time=3] | 0^a^ | 0 |  |  |  |  |  |
| [time=1] * [Group=1] | 1.459706 | 1.340857 | 168.626 | 1.089 | .278 | -1.187323 | 4.106735 |
| [time=2] * [Group=1] | .428329 | 1.281313 | 159.943 | .334 | .739 | -2.102145 | 2.958804 |
| [time=3] * [Group=1] | 0^a^ | 0 |  |  |  |  |  |
| [time=1] * [Group=2] | -1.309716 | 1.339684 | 170.436 | -.978 | .330 | -3.954227 | 1.334795 |
| [time=2] * [Group=2] | -.727972 | 1.304845 | 162.417 | -.558 | .578 | -3.304620 | 1.848676 |
| [time=3] * [Group=2] | 0^a^ | 0 |  |  |  |  |  |
| [time=1] * [Group=3] | 0^a^ | 0 |  |  |  |  |  |
| [time=2] * [Group=3] | 0^a^ | 0 |  |  |  |  |  |
| [time=3] * [Group=3] | 0^a^ | 0 |  |  |  |  |  |
| HADS_Depressive symptoms_T0 | .368230 | .109223 | 247.141 | 3.371 | .001 | .153104 | .583357 |
| [Group=1] * HADS_Depressive symptoms_T0 | .260447 | .177755 | 242.785 | 1.465 | .144 | -.089691 | .610585 |
| [Group=2] * HADS_Depressive symptoms_T0 | -.170238 | .192647 | 250.601 | -.884 | .378 | -.549652 | .209176 |
| [Group=3] * HADS_Depressive symptoms_T0 | 0^a^ | 0 |  |  |  |  |  |
| [time=1] * HADS_Depressive symptoms_T0 | .461295 | .138773 | 167.495 | 3.324 | .001 | .187325 | .735265 |
| [time=2] * HADS_Depressive symptoms_T0 | .036325 | .135637 | 162.915 | .268 | .789 | -.231509 | .304159 |
| [time=3] * HADS_Depressive symptoms_T0 | 0^a^ | 0 |  |  |  |  |  |
| [time=1] * [Group=1] * HADS_Depressive symptoms_T0 | -.529754 | .226456 | 162.610 | -2.339 | .021 | -.976928 | -.082581 |
| [time=2] * [Group=1] * HADS_Depressive symptoms_T0 | -.197360 | .222398 | 159.449 | -.887 | .376 | -.636586 | .241866 |
| [time=3] * [Group=1] * HADS_Depressive symptoms_T0 | 0^a^ | 0 |  |  |  |  |  |
| [time=1] * [Group=2] * HADS_Depressive symptoms_T0 | .243661 | .236608 | 167.220 | 1.030 | .305 | -.223463 | .710784 |
| [time=2] * [Group=2] * HADS_Depressive symptoms_T0 | .005144 | .236619 | 162.744 | .022 | .983 | -.462095 | .472383 |
| [time=3] * [Group=2] * HADS_Depressive symptoms_T0 | 0^a^ | 0 |  |  |  |  |  |
| [time=1] * [Group=3] * HADS_Depressive symptoms_T0 | 0^a^ | 0 |  |  |  |  |  |
| [time=2] * [Group=3] * HADS_Depressive symptoms_T0 | 0^a^ | 0 |  |  |  |  |  |
| [time=3] * [Group=3] * HADS_Depressive symptoms_T0 | 0^a^ | 0 |  |  |  |  |  |
| Notes: Group 1 = SMC, Group 2 = SUPPORT, Group 3 = EXPECT. Time 1 = 1 day pre-surgery; time 2 = 1 week post-surgery; time 3 = 6 months follow-up-assessment. Dependent Variable: HADS_Depressive symptoms. a. This parameter is set to zero because it is redundant. | | | | | | | |

| *Estimates of Covariance Parameters* | | | |  |  |  |  |
| --- | --- | --- | --- | --- | --- | --- | --- |
| Parameter | | Estimate | Std. Error | Wald Z | Sig. | 95% Confidence Interval | |
|  |  |  |  |  |  | Lower Bound | Upper Bound |
| Repeated Measures | CS diagonal offset | 4.752628 | .538918 | 8.819 | <.001 | 3.805510 | 5.935465 |
|  | CS covariance | 1.359361 | .529897 | 2.565 | .010 | .320781 | 2.397940 |
| Intercept | Variance | .442235 | 33554432.000 | .000 | 1.000 | .000000 |  |
| Notes: Dependent Variable: HADS_Depressive symptoms. | | | |  |  |  |  |

| *Estimates for Means (group)* | | | | | |
| --- | --- | --- | --- | --- | --- |
| group | Mean | Std. Error | df | 95% Confidence Interval | |
|  |  |  |  | Lower Bound | Upper Bound |
| SMC | 4.683 | .724 | .000 | 2.195 | 7.171 |
| SUPPORT | 3.324 | .742 | .000 | .807 | 5.841 |
| EXPECT | 3.983 | .731 | .000 | 1.702 | 6.265 |
| Notes: Dependent Variable: HADS_Depressive symptoms. Adjusted score T0 (baseline) = 4.5355. | | | | | |

| *Estimates for Means (time)* | | | | | |
| --- | --- | --- | --- | --- | --- |
| time | Mean | Std. Error | df | 95% Confidence Interval | |
|  |  |  |  | Lower Bound | Upper Bound |
| 1 | 4.683 | .724 | .000 | 2.195 | 7.171 |
| 2 | 3.324 | .742 | .000 | .807 | 5.841 |
| 3 | 3.983 | .731 | .000 | 1.702 | 6.265 |
| Notes: Dependent Variable: HADS_Depressive symptoms. Time 1 = 1 day pre-surgery; time 2 = 1 week post-surgery; time 3 = 6 months follow-up-assessment. Adjusted score T0 (baseline) = 4.5355. | | | | | |

| *Estimates for level of depressive symptoms (average (mean))* | | | | | | |
| --- | --- | --- | --- | --- | --- | --- |
| group | time | Mean | Std. Error | df | 95% Confidence Interval | |
|  |  |  |  |  | Lower Bound | Upper Bound |
| SMC | 1 | 4.998 | .785 | .000 | 2.111 | 7.885 |
|  | 2 | 5.402 | .788 | .000 | 2.138 | 8.666 |
|  | 3 | 3.649 | .782 | .000 | .201 | 7.097 |
| SUPPORT | 1 | 4.211 | .803 | .000 | .980 | 7.442 |
|  | 2 | 3.639 | .828 | .000 | -.367 | 7.645 |
|  | 3 | 2.123 | .834 | .000 | -1.648 | 5.895 |
| EXPECT | 1 | 4.771 | .806 | .000 | 1.069 | 8.474 |
|  | 2 | 4.699 | .790 | .000 | 1.760 | 7.639 |
|  | 3 | 2.479 | .805 | .000 | -1.470 | 6.429 |
| Notes: Dependent Variable: HADS_Depressive symptoms. Time 1 = 1 day pre-surgery; time 2 = 1 week post-surgery; time 3 = 6 months follow-up-assessment. Adjusted score T0 (baseline) = 4.5355. | | | | | | |

**Mixed Model Analysis – Results for Anxiety**

| *Model Dimension* | | | | | | |
| --- | --- | --- | --- | --- | --- | --- |
|  | | Number of Levels | Covariance Structure | Number of Parameters | Subject Variables | Number of Subjects |
| Fixed Effects | Intercept | 1 |  | 1 |  |  |
|  | Group | 3 |  | 2 |  |  |
|  | time | 3 |  | 2 |  |  |
|  | Group * time | 9 |  | 4 |  |  |
|  | HADS_Anxiety_T0 | 1 |  | 1 |  |  |
|  | Group * HADS_Anxiety_T0 | 3 |  | 2 |  |  |
|  | time * HADS_Anxiety_T0 | 3 |  | 2 |  |  |
|  | Group * time * HADS_Anxiety_T0 | 9 |  | 4 |  |  |
| Random Effects | Intercept | 1 | Variance Components | 1 |  |  |
| Repeated Effects | time | 3 | Compound Symmetry | 2 | id | 107 |
| Total | | 36 |  | 21 |  |  |
| Notes: Dependent Variable: HADS_Anxiety. | | | | | | |

| *Type III Tests of Fixed Effects* | | | | |
| --- | --- | --- | --- | --- |
| Source | Numerator df | Denominator df | F | Sig. |
| Intercept | 1 | 100.016 | 36.266 | <.001 |
| Group | 2 | 99.792 | .437 | .647 |
| time | 2 | 180.536 | .381 | .684 |
| Group * time | 4 | 180.192 | .714 | .583 |
| HADS_Anxiety_T0 | 1 | 102.020 | 62.390 | <.001 |
| Group * HADS_Anxiety_T0 | 2 | 101.694 | 1.230 | .297 |
| time * HADS_Anxiety_T0 | 2 | 182.055 | 7.296 | .001 |
| Group * time * HADS_Anxiety_T0 | 4 | 181.434 | 1.547 | .191 |
| Notes: Dependent Variable: HADS_Anxiety. | | | | |

| *Estimates of Fixed Effects* | | | | | | | |
| --- | --- | --- | --- | --- | --- | --- | --- |
| Parameter | Estimate | Std. Error | df | t | Sig. | 95% Confidence Interval | |
|  |  |  |  |  |  | Lower Bound | Upper Bound |
| Intercept | .855267 | .656510 | 245.350 | 1.303 | .194 | -.437848 | 2.148382 |
| [Group=1] | .783619 | .970264 | 241.515 | .808 | .420 | -1.127640 | 2.694878 |
| [Group=2] | 1.428464 | 1.085202 | 248.679 | 1.316 | .189 | -.708895 | 3.565823 |
| [Group=3] | 0^a^ | 0 |  |  |  |  |  |
| [time=1] | 1.294259 | .821888 | 187.785 | 1.575 | .117 | -.327061 | 2.915580 |
| [time=2] | .626175 | .775536 | 174.147 | .807 | .421 | -.904484 | 2.156834 |
| [time=3] | 0^a^ | 0 |  |  |  |  |  |
| [time=1] * [Group=1] | -.676903 | 1.177795 | 179.162 | -.575 | .566 | -3.001037 | 1.647232 |
| [time=2] * [Group=1] | .229132 | 1.176761 | 176.205 | .195 | .846 | -2.093227 | 2.551491 |
| [time=3] * [Group=1] | 0^a^ | 0 |  |  |  |  |  |
| [time=1] * [Group=2] | -2.128129 | 1.330075 | 186.926 | -1.600 | .111 | -4.752017 | .495759 |
| [time=2] * [Group=2] | -.832056 | 1.297561 | 178.209 | -.641 | .522 | -3.392618 | 1.728505 |
| [time=3] * [Group=2] | 0^a^ | 0 |  |  |  |  |  |
| [time=1] * [Group=3] | 0^a^ | 0 |  |  |  |  |  |
| [time=2] * [Group=3] | 0^a^ | 0 |  |  |  |  |  |
| [time=3] * [Group=3] | 0^a^ | 0 |  |  |  |  |  |
| HADS_Anxiety_T0 | .521845 | .090517 | 243.179 | 5.765 | <.001 | .343549 | .700142 |
| [Group=1] * HADS_Anxiety_T0 | -.143787 | .170822 | 238.675 | -.842 | .401 | -.480298 | .192724 |
| [Group=2] * HADS_Anxiety_T0 | -.459085 | .175276 | 243.241 | -2.619 | .009 | -.804336 | -.113833 |
| [Group=3] * HADS_Anxiety_T0 | 0^a^ | 0 |  |  |  |  |  |
| [time=1] * HADS_Anxiety_T0 | .100249 | .109485 | 179.337 | .916 | .361 | -.115795 | .316293 |
| [time=2] * HADS_Anxiety_T0 | -.072297 | .109564 | 177.539 | -.660 | .510 | -.288513 | .143919 |
| [time=3] * HADS_Anxiety_T0 | 0^a^ | 0 |  |  |  |  |  |
| [time=1] * [Group=1] * HADS_Anxiety_T0 | .132555 | .204882 | 174.150 | .647 | .518 | -.271815 | .536926 |
| [time=2] * [Group=1] * HADS_Anxiety_T0 | .065317 | .216900 | 181.169 | .301 | .764 | -.362658 | .493291 |
| [time=3] * [Group=1] * HADS_Anxiety_T0 | 0^a^ | 0 |  |  |  |  |  |
| [time=1] * [Group=2] * HADS_Anxiety_T0 | .556764 | .224082 | 186.952 | 2.485 | .014 | .114710 | .998818 |
| [time=2] * [Group=2] * HADS_Anxiety_T0 | .226366 | .211867 | 177.125 | 1.068 | .287 | -.191743 | .644475 |
| [time=3] * [Group=2] * HADS_Anxiety_T0 | 0^a^ | 0 |  |  |  |  |  |
| [time=1] * [Group=3] * HADS_Anxiety_T0 | 0^a^ | 0 |  |  |  |  |  |
| [time=2] * [Group=3] * HADS_Anxiety_T0 | 0^a^ | 0 |  |  |  |  |  |
| [time=3] * [Group=3] * HADS_Anxiety_T0 | 0^a^ | 0 |  |  |  |  |  |
| Notes: Group 1 = SMC, Group 2 = SUPPORT, Group 3 = EXPECT. Time 1 = 1 day pre-surgery; time 2 = 1 week post-surgery; time 3 = 6 months follow-up-assessment. Dependent Variable: HADS_Anxiety. a. This parameter is set to zero because it is redundant. | | | | | | | |

| *Estimates of Covariance Parameters* | | | |  |  |  |  |
| --- | --- | --- | --- | --- | --- | --- | --- |
| Parameter | | Estimate | Std. Error | Wald Z | Sig. | 95% Confidence Interval | |
|  |  |  |  |  |  | Lower Bound | Upper Bound |
| Repeated Measures | CS diagonal offset | 4.576303 | .498507 | 9.180 | <.001 | 3.696506 | 5.665497 |
|  | CS covariance | 1.943736 | .556397 | 3.493 | <.001 | .853219 | 3.034253 |
| Intercept | Variance | .000000^a^ | .000 |  |  |  |  |
| Notes: Dependent Variable: HADS_Anxiety. a. This parameter is set to zero because it is redundant. | | | | | | | |

| *Estimates for Means (group)* | | | | | |
| --- | --- | --- | --- | --- | --- |
| group | Mean | Std. Error | df | 95% Confidence Interval | |
|  |  |  |  | Lower Bound | Upper Bound |
| SMC | 4.190 | .317 | 97.000 | 3.561 | 4.819 |
| SUPPORT | 3.451 | .337 | 98.627 | 2.783 | 4.120 |
| EXPECT | 3.910 | .325 | 100.200 | 3.265 | 4.555 |
| Notes: Dependent Variable: HADS_Anxiety. Adjusted score T0 (baseline) = 4.5451. | | | | | |

| *Estimates for Means (time)* | | | | | |
| --- | --- | --- | --- | --- | --- |
| time | Mean | Std. Error | df | 95% Confidence Interval | |
|  |  |  |  | Lower Bound | Upper Bound |
| 1 | 4.910 | .263 | 244.266 | 4.392 | 5.428 |
| 2 | 3.590 | .266 | 245.431 | 3.066 | 4.113 |
| 3 | 3.051 | .262 | 242.318 | 2.536 | 3.566 |
| Notes: Dependent Variable: HADS_Anxiety. Time 1 = 1 day pre-surgery; time 2 = 1 week post-surgery; time 3 = 6 months follow-up-assessment. Adjusted score T0 (baseline) = 4.5451. | | | | | |

| *Estimates for level of HADS Anxiety (average (mean))* | | | | | | |
| --- | --- | --- | --- | --- | --- | --- |
| group | time | Mean | Std. Error | df | 95% Confidence Interval | |
|  |  |  |  |  | Lower Bound | Upper Bound |
| SMC | 1 | 5.033 | .442 | 242.678 | 4.162 | 5.904 |
|  | 2 | 4.181 | .452 | 246.963 | 3.291 | 5.071 |
|  | 3 | 3.357 | .432 | 237.795 | 2.506 | 4.209 |
| SUPPORT | 1 | 4.721 | .459 | 240.256 | 3.816 | 5.626 |
|  | 2 | 3.063 | .478 | 246.698 | 2.121 | 4.006 |
|  | 3 | 2.569 | .473 | 244.460 | 1.637 | 3.501 |
| EXPECT | 1 | 4.977 | .465 | 249.320 | 4.062 | 5.892 |
|  | 2 | 3.525 | .450 | 242.358 | 2.639 | 4.411 |
|  | 3 | 3.227 | .453 | 243.949 | 2.334 | 4.120 |
| Notes: Dependent Variable: HADS_Anxiety. Time 1 = 1 day pre-surgery; time 2 = 1 week post-surgery; time 3 = 6 months follow-up-assessment. Adjusted score T0 (baseline) = 4.5451. | | | | | | |

**Mixed Model Analysis – Results for HADS sumscore**

| *Model Dimension* | | | | | | |
| --- | --- | --- | --- | --- | --- | --- |
|  | | Number of Levels | Covariance Structure | Number of Parameters | Subject Variables | Number of Subjects |
| Fixed Effects | Intercept | 1 |  | 1 |  |  |
|  | Group | 3 |  | 2 |  |  |
|  | time | 3 |  | 2 |  |  |
|  | Group * time | 9 |  | 4 |  |  |
|  | HADS_sumscore_T0 | 1 |  | 1 |  |  |
|  | Group * HADS_sumscore_T0 | 3 |  | 2 |  |  |
|  | time * HADS_sumscore_T0 | 3 |  | 2 |  |  |
|  | Group * time * HADS_sumscore_T0 | 9 |  | 4 |  |  |
| Random Effects | Intercept | 1 | Variance Components | 1 |  |  |
| Repeated Effects | time | 3 | Heterogeneous First-Order Autoregressive | 4 | id | 106 |
| Total | | 36 |  | 23 |  |  |
| Notes: Dependent Variable: HADS_sumscore. | | | | | | |

| *Type III Tests of Fixed Effects* | | | | |
| --- | --- | --- | --- | --- |
| Source | Numerator df | Denominator df | F | Sig. |
| Intercept | 1 | 98.608 | 40.063 | <.001 |
| Group | 2 | 98.658 | .512 | .601 |
| time | 2 | 132.936 | 4.559 | .012 |
| Group * time | 4 | 133.152 | .411 | .800 |
| HADS_sumscore_T0 | 1 | 101.298 | 103.028 | <.001 |
| Group * HADS_sumscore_T0 | 2 | 101.009 | .926 | .399 |
| time * HADS_sumscore_T0 | 2 | 136.142 | 10.853 | <.001 |
| Group * time * HADS_sumscore_T0 | 4 | 136.404 | 1.178 | .323 |
| Notes: Dependent Variable: HADS_sumscore. | | | | |

| *Estimates of Fixed Effects* | | | | | | | |
| --- | --- | --- | --- | --- | --- | --- | --- |
| Parameter | Estimate | Std. Error | df | t | Sig. | 95% Confidence Interval | |
|  |  |  |  |  |  | Lower Bound | Upper Bound |
| Intercept | 1.615193 | 1.133758 | 88.224 | 1.425 | .158 | -.637833 | 3.868219 |
| [Group=1] | .777636 | 1.727207 | 88.019 | .450 | .654 | -2.654815 | 4.210087 |
| [Group=2] | 1.049399 | 1.843880 | 88.324 | .569 | .571 | -2.614737 | 4.713535 |
| [Group=3] | 0^a^ | 0 |  |  |  |  |  |
| [time=1] | .922652 | 1.565628 | 169.104 | .589 | .556 | -2.168042 | 4.013346 |
| [time=2] | 2.562684 | 1.387588 | 119.421 | 1.847 | .067 | -.184780 | 5.310148 |
| [time=3] | 0^a^ | 0 |  |  |  |  |  |
| [time=1] * [Group=1] | .829352 | 2.351885 | 168.582 | .353 | .725 | -3.813588 | 5.472293 |
| [time=2] * [Group=1] | .337047 | 2.158870 | 118.789 | .156 | .876 | -3.937810 | 4.611905 |
| [time=3] * [Group=1] | 0^a^ | 0 |  |  |  |  |  |
| [time=1] * [Group=2] | -2.196104 | 2.474286 | 169.024 | -.888 | .376 | -7.080588 | 2.688381 |
| [time=2] * [Group=2] | -.398176 | 2.239330 | 119.250 | -.178 | .859 | -4.832177 | 4.035825 |
| [time=3] * [Group=2] | 0^a^ | 0 |  |  |  |  |  |
| [time=1] * [Group=3] | 0^a^ | 0 |  |  |  |  |  |
| [time=2] * [Group=3] | 0^a^ | 0 |  |  |  |  |  |
| [time=3] * [Group=3] | 0^a^ | 0 |  |  |  |  |  |
| HADS_sumscore_T0 | .401384 | .085065 | 88.152 | 4.719 | .000 | .232339 | .570428 |
| [Group=1] * HADS_sumscore_T0 | .115036 | .158136 | 87.917 | .727 | .469 | -.199231 | .429302 |
| [Group=2] * HADS_sumscore_T0 | -.204588 | .166145 | 88.316 | -1.231 | .221 | -.534750 | .125575 |
| [Group=3] * HADS_sumscore_T0 | 0^a^ | 0 |  |  |  |  |  |
| [time=1] * HADS_sumscore_T0 | .365010 | .113656 | 168.713 | 3.212 | .002 | .140639 | .589382 |
| [time=2] * HADS_sumscore_T0 | .028469 | .104730 | 118.279 | .272 | .786 | -.178920 | .235858 |
| [time=3] * HADS_sumscore_T0 | 0^a^ | 0 |  |  |  |  |  |
| [time=1] * [Group=1] * HADS_sumscore_T0 | -.224620 | .212517 | 168.274 | -1.057 | .292 | -.644163 | .194924 |
| [time=2] * [Group=1] * HADS_sumscore_T0 | -.054114 | .201662 | 118.666 | -.268 | .789 | -.453437 | .345209 |
| [time=3] * [Group=1] * HADS_sumscore_T0 | 0^a^ | 0 |  |  |  |  |  |
| [time=1] * [Group=2] * HADS_sumscore_T0 | .253551 | .227557 | 170.356 | 1.114 | .267 | -.195643 | .702745 |
| [time=2] * [Group=2] * HADS_sumscore_T0 | -.055668 | .202525 | 119.324 | -.275 | .784 | -.456676 | .345341 |
| [time=3] * [Group=2] * HADS_sumscore_T0 | 0^a^ | 0 |  |  |  |  |  |
| [time=1] * [Group=3] * HADS_sumscore_T0 | 0^a^ | 0 |  |  |  |  |  |
| [time=2] * [Group=3] * HADS_sumscore_T0 | 0^a^ | 0 |  |  |  |  |  |
| [time=3] * [Group=3] * HADS_sumscore_T0 | 0^a^ | 0 |  |  |  |  |  |
| Notes: Group 1 = SMC, Group 2 = SUPPORT, Group 3 = EXPECT. Time 1 = 1 day pre-surgery; time 2 = 1 week post-surgery; time 3 = 6 months follow-up-assessment. Dependent Variable: HADS_sumscore. a. This parameter is set to zero because it is redundant. | | | | | | | |

| *Estimates of Covariance Parameters* | | | | | | | |
| --- | --- | --- | --- | --- | --- | --- | --- |
| Parameter | | Estimate | Std. Error | Wald Z | Sig. | 95% Confidence Interval | |
|  |  |  |  |  |  | Lower Bound | Upper Bound |
| Repeated Measures | Var: [time = 1] | 15.773084 | 2.350735 | 6.710 | <.001 | 11.777636 | 21.123947 |
|  | Var: [time = 2] | 18.533902 | 2.869617 | 6.459 | <.001 | 13.682793 | 25.104928 |
|  | Var: [time = 3] | 16.458369 | 2.485771 | 6.621 | <.001 | 12.241272 | 22.128249 |
|  | ARH1, rho | .276863 | .077799 | 3.559 | <.001 | .118578 | .421425 |
| Intercept | Variance | .000000^a^ | .000000 |  |  |  |  |
| Notes: Dependent Variable: HADS_sumscore. Time 1 = 1 day pre-surgery; time 2 = 1 week post-surgery; time 3 = 6 months follow-up-assessment. a. This parameter is set to zero because it is redundant. | | | | | | | |

| *Estimates for Means (group)* | | | | | |
| --- | --- | --- | --- | --- | --- |
| group | Mean | Std. Error | df | 95% Confidence Interval | |
|  |  |  |  | Lower Bound | Upper Bound |
| SMC | 8.900 | .476 | 96.570 | 7.955 | 9.844 |
| SUPPORT | 6.481 | .540 | 103.007 | 5.410 | 7.553 |
| EXPECT | 7.536 | .505 | 102.284 | 6.535 | 8.536 |
| Notes: Dependent Variable: HADS_sumscore. Adjusted score T0 (baseline) = 8.9355. | | | | | |

| *Estimates for Means (time)* | | | | | |
| --- | --- | --- | --- | --- | --- |
| time | Mean | Std. Error | df | 95% Confidence Interval | |
|  |  |  |  | Lower Bound | Upper Bound |
| 1 | 9.359 | .414 | 90.851 | 8.537 | 10.181 |
| 2 | 8.014 | .452 | 85.960 | 7.115 | 8.913 |
| 3 | 5.544 | .431 | 88.157 | 4.688 | 6.401 |
| Notes: Dependent Variable: HADS_sumscore. Time 1 = 1 day pre-surgery; time 2 = 1 week post-surgery; time 3 = 6 months follow-up-assessment. Adjusted score T0 (baseline) = 8.9355. | | | | | |

| *Estimates for level of HADS sumcore (average (mean))* | | | | | | |
| --- | --- | --- | --- | --- | --- | --- |
| group | time | Mean | Std. Error | df | 95% Confidence Interval | |
|  |  |  |  |  | Lower Bound | Upper Bound |
| SMC | 1 | 10.014 | .674 | 90.609 | 8.675 | 11.353 |
|  | 2 | 9.678 | .742 | 86.012 | 8.202 | 11.153 |
|  | 3 | 7.007 | .679 | 87.937 | 5.658 | 8.357 |
| SUPPORT | 1 | 8.677 | .741 | 90.710 | 7.205 | 10.149 |
|  | 2 | 6.345 | .843 | 86.503 | 4.668 | 8.021 |
|  | 3 | 4.423 | .811 | 88.221 | 2.812 | 6.034 |
| EXPECT | 1 | 9.386 | .733 | 91.162 | 7.930 | 10.842 |
|  | 2 | 8.019 | .761 | 85.208 | 6.506 | 9.531 |
|  | 3 | 5.202 | .744 | 88.243 | 3.723 | 6.681 |
| Notes: Dependent Variable: HADS_sumscore. Time 1 = 1 day pre-surgery; time 2 = 1 week post-surgery; time 3 = 6 months follow-up-assessment. Adjusted score T0 (baseline) = 8.9355. | | | | | | |

**Mixed Model Analysis – Results for Consequences**

| *Model Dimension* | | | | | | |
| --- | --- | --- | --- | --- | --- | --- |
|  | | Number of Levels | Covariance Structure | Number of Parameters | Subject Variables | Number of Subjects |
| Fixed Effects | Intercept | 1 |  | 1 |  |  |
|  | Group | 3 |  | 2 |  |  |
|  | time | 3 |  | 2 |  |  |
|  | Group * time | 9 |  | 4 |  |  |
|  | B-IPQ_Consequences_T0 | 1 |  | 1 |  |  |
|  | Group * B-IPQ_Consequences_T0 | 3 |  | 2 |  |  |
|  | time * B-IPQ_Consequences_T0 | 3 |  | 2 |  |  |
|  | Group * time * B-IPQ_Consequences_T0 | 9 |  | 4 |  |  |
| Random Effects | Intercept | 1 | Variance Components | 1 |  |  |
| Repeated Effects | time | 3 | Compound Symmetry | 2 | id | 110 |
| Total | | 36 |  | 21 |  |  |
| Notes: Dependent Variable: B-IPQ_Consequences. | | | | | | |

| *Type III Tests of Fixed Effects* | | | | |
| --- | --- | --- | --- | --- |
| Source | Numerator df | Denominator df | F | Sig. |
| Intercept | 1 | .000 | 55.729 | <.001 |
| Group | 2 | 107.344 | 1.259 | .288 |
| time | 2 | 197.111 | 26.836 | <.001 |
| Group * time | 4 | 196.677 | 2.791 | .028 |
| B-IPQ_Consequences_T0 | 1 | 104.670 | 49.551 | <.001 |
| Group * B-IPQ_Consequences_T0 | 2 | 104.601 | .744 | .478 |
| time * B-IPQ_Consequences_T0 | 2 | 194.287 | 9.969 | <.001 |
| Group * time * B-IPQ_Consequences_T0 | 4 | 194.173 | 2.215 | .069 |
| Notes: Dependent Variable: B-IPQ_Consequences. | | | | |

| *Estimates of Fixed Effects* | | | | | | | |
| --- | --- | --- | --- | --- | --- | --- | --- |
| Parameter | Estimate | Std. Error | df | t | Sig. | 95% Confidence Interval | |
|  |  |  |  |  |  | Lower Bound | Upper Bound |
| Intercept | 1.147365 | .854715 | .000 | 1.342 | 1.000 | -19.649065 | 21.943795 |
| [Group=1] | 1.451203 | 1.047647 | 276.937 | 1.385 | .167 | -.611161 | 3.513566 |
| [Group=2] | .795806 | 1.043473 | 277.797 | .763 | .446 | -1.258312 | 2.849924 |
| [Group=3] | 0^a^ | 0 |  |  |  |  |  |
| [time=1] | 2.985743 | 1.047499 | 197.220 | 2.850 | .005 | .920006 | 5.051479 |
| [time=2] | 4.136594 | 1.082469 | 204.394 | 3.821 | <.001 | 2.002357 | 6.270831 |
| [time=3] | 0^a^ | 0 |  |  |  |  |  |
| [time=1] * [Group=1] | -3.711669 | 1.414141 | 194.636 | -2.625 | .009 | -6.500675 | -.922662 |
| [time=2] * [Group=1] | .066196 | 1.436504 | 198.184 | .046 | .963 | -2.766599 | 2.898991 |
| [time=3] * [Group=1] | 0^a^ | 0 |  |  |  |  |  |
| [time=1] * [Group=2] | -3.680580 | 1.386161 | 196.730 | -2.655 | .009 | -6.414222 | -.946938 |
| [time=2] * [Group=2] | -.875014 | 1.424775 | 200.306 | -.614 | .540 | -3.684497 | 1.934468 |
| [time=3] * [Group=2] | 0^a^ | 0 |  |  |  |  |  |
| [time=1] * [Group=3] | 0^a^ | 0 |  |  |  |  |  |
| [time=2] * [Group=3] | 0^a^ | 0 |  |  |  |  |  |
| [time=3] * [Group=3] | 0^a^ | 0 |  |  |  |  |  |
| B-IPQ_Consequences_T0 | .327642 | .125144 | 276.853 | 2.618 | .009 | .081287 | .573997 |
| [Group=1] * B-IPQ_Consequences_T0 | -.194817 | .175879 | 276.365 | -1.108 | .269 | -.541049 | .151415 |
| [Group=2] * B-IPQ_Consequences_T0 | -.099688 | .170500 | 277.400 | -.585 | .559 | -.435326 | .235950 |
| [Group=3] * B-IPQ_Consequences_T0 | 0^a^ | 0 |  |  |  |  |  |
| [time=1] * B-IPQ_Consequences_T0 | -.031019 | .169621 | 194.985 | -.183 | .855 | -.365546 | .303508 |
| [time=2] * B-IPQ_Consequences_T0 | -.090667 | .171848 | 197.917 | -.528 | .598 | -.429556 | .248221 |
| [time=3] * B-IPQ_Consequences_T0 | 0^a^ | 0 |  |  |  |  |  |
| [time=1] * [Group=1] * B-IPQ_Consequences_T0 | .556036 | .237314 | 191.785 | 2.343 | .020 | .087956 | 1.024116 |
| [time=2] * [Group=1] * B-IPQ_Consequences_T0 | -.008773 | .240399 | 194.485 | -.036 | .971 | -.482897 | .465352 |
| [time=3] * [Group=1] * B-IPQ_Consequences_T0 | 0^a^ | 0 |  |  |  |  |  |
| [time=1] * [Group=2] * B-IPQ_Consequences_T0 | .527954 | .227830 | 194.862 | 2.317 | .022 | .078624 | .977284 |
| [time=2] * [Group=2] * B-IPQ_Consequences_T0 | .088821 | .229715 | 196.159 | .387 | .699 | -.364208 | .541849 |
| [time=3] * [Group=2] * B-IPQ_Consequences_T0 | 0^a^ | 0 |  |  |  |  |  |
| [time=1] * [Group=3] * B-IPQ_Consequences_T0 | 0^a^ | 0 |  |  |  |  |  |
| [time=2] * [Group=3] * B-IPQ_Consequences_T0 | 0^a^ | 0 |  |  |  |  |  |
| [time=3] * [Group=3] * B-IPQ_Consequences_T0 | 0^a^ | 0 |  |  |  |  |  |
| Notes: Group 1 = SMC, Group 2 = SUPPORT, Group 3 = EXPECT. Time 1 = 1 day pre-surgery; time 2 = 1 week post-surgery; time 3 = 6 months follow-up-assessment. Dependent Variable: B-IPQ_Consequences. a. This parameter is set to zero because it is redundant. | | | | | | | |

| *Estimates of Covariance Parameters* | | | |  |  |  |  |
| --- | --- | --- | --- | --- | --- | --- | --- |
| Parameter | | Estimate | Std. Error | Wald Z | Sig. | 95% Confidence Interval | |
|  |  |  |  |  |  | Lower Bound | Upper Bound |
| Repeated Measures | CS diagonal offset | 4.193625 | .436466 | 9.608 | <.001 | 3.419777 | 5.142583 |
|  | CS covariance | .566352 | .321814 | 1.760 | .078 | -.064392 | 1.197096 |
| Intercept | Variance | .129902 | 6341191.604 | .000 | 1.000 | .000000 |  |
| Notes: Dependent Variable: B-IPQ_Consequences | | | |  |  |  |  |

| *Estimates for Means (group)* | | | | | |
| --- | --- | --- | --- | --- | --- |
| group | Mean | Std. Error | df | 95% Confidence Interval | |
|  |  |  |  | Lower Bound | Upper Bound |
| SMC | 5.156 | .429 | .000 | 1.956 | 8.355 |
| SUPPORT | 4.799 | .437 | .000 | 2.126 | 7.472 |
| EXPECT | 4.983 | .436 | .000 | 1.733 | 8.232 |
| Notes: Dependent Variable: B-IPQ_Consequences. Adjusted score T0 (baseline) = 5.09. | | | | | |

| *Estimates for Means (time)* | | | | | |
| --- | --- | --- | --- | --- | --- |
| time | Mean | Std. Error | df | 95% Confidence Interval | |
|  |  |  |  | Lower Bound | Upper Bound |
| 1 | 5.267 | .421 | .000 | 2.608 | 7.927 |
| 2 | 6.606 | .423 | .000 | 3.485 | 9.727 |
| 3 | 3.064 | .422 | .000 | -.395 | 6.524 |
| Notes: Dependent Variable: B-IPQ_Consequences. Time 1 = 1 day pre-surgery; time 2 = 1 week post-surgery; time 3 = 6 months follow-up-assessment. Adjusted score T0 (baseline) = 5.09. | | | | | |

| *Estimates for level of B-IPQ Consequences (average (mean))* | | | | | | |
| --- | --- | --- | --- | --- | --- | --- |
| group | time | Mean | Std. Error | df | 95% Confidence Interval | |
|  |  |  |  |  | Lower Bound | Upper Bound |
| SMC | 1 | 5.221 | .512 | .000 | -4.033 | 14.476 |
|  | 2 | 6.971 | .517 | .000 | .299 | 13.643 |
|  | 3 | 3.275 | .512 | .000 | -5.252 | 11.802 |
| SUPPORT | 1 | 4.938 | .524 | .000 | -4.099 | 13.975 |
|  | 2 | 6.356 | .532 | .000 | 1.432 | 11.279 |
|  | 3 | 3.103 | .532 | .000 | -1.772 | 7.979 |
| EXPECT | 1 | 5.643 | .525 | .000 | -2.559 | 13.845 |
|  | 2 | 6.490 | .529 | .000 | -2.313 | 15.294 |
|  | 3 | 2.815 | .529 | .000 | -6.012 | 11.642 |
| Notes: Dependent Variable: B-IPQ_Consequences. Time 1 = 1 day pre-surgery; time 2 = 1 week post-surgery; time 3 = 6 months follow-up-assessment. Adjusted score T0 (baseline) = 5.09. | | | | | | |

**Mixed Model Analysis – Results for Timeline**

| *Model Dimension* | | | | | | |
| --- | --- | --- | --- | --- | --- | --- |
|  | | Number of Levels | Covariance Structure | Number of Parameters | Subject Variables | Number of Subjects |
| Fixed Effects | Intercept | 1 |  | 1 |  |  |
|  | Group | 3 |  | 2 |  |  |
|  | time | 3 |  | 2 |  |  |
|  | Group * time | 9 |  | 4 |  |  |
|  | B-IPQ_Timeline_T0 | 1 |  | 1 |  |  |
|  | Group * B-IPQ_Timeline_T0 | 3 |  | 2 |  |  |
|  | time * B-IPQ_Timeline_T0 | 3 |  | 2 |  |  |
|  | Group * time * B-IPQ_Timeline_T0 | 9 |  | 4 |  |  |
| Random Effects | Intercept | 1 | Variance Components | 1 |  |  |
| Repeated Effects | time | 3 | Heterogeneous First-Order Autoregressive | 4 | id | 105 |
| Total | | 36 |  | 23 |  |  |
| Notes: Dependent Variable: B-IPQ_Timeline. | | | | | | |

| *Type III Tests of Fixed Effects* | | | | |
| --- | --- | --- | --- | --- |
| Source | Numerator df | Denominator df | F | Sig. |
| Intercept | 1 | 114.661 | 146.372 | <.001 |
| Group | 2 | 114.511 | .613 | .544 |
| time | 2 | 121.793 | 13.087 | <.001 |
| Group * time | 4 | 121.741 | .708 | .588 |
| B-IPQ_Timeline_T0 | 1 | 118.586 | 37.999 | <.001 |
| Group * B-IPQ_Timeline_T0 | 2 | 116.593 | .118 | .889 |
| time * B-IPQ_Timeline_T0 | 2 | 120.799 | 1.516 | .224 |
| Group * time * B-IPQ_Timeline_T0 | 4 | 121.999 | .541 | .706 |
| Notes: Dependent Variable: B-IPQ_Timeline. | | | | |

| *Estimates of Fixed Effects* | | | | | | | |
| --- | --- | --- | --- | --- | --- | --- | --- |
| Parameter | Estimate | Std. Error | df | t | Sig. | 95% Confidence Interval | |
|  |  |  |  |  |  | Lower Bound | Upper Bound |
| Intercept | 4.342419 | .859344 | 87.574 | 5.053 | <.001 | 2.634537 | 6.050302 |
| [Group=1] | -1.114645 | 1.152063 | 87.571 | -.968 | .336 | -3.404285 | 1.174994 |
| [Group=2] | -1.775604 | 1.248052 | 87.574 | -1.423 | .158 | -4.256014 | .704806 |
| [Group=3] | 0^a^ | 0 |  |  |  |  |  |
| [time=1] | -2.474285 | .986480 | 138.422 | -2.508 | .013 | -4.424802 | -.523767 |
| [time=2] | -1.174955 | 1.003938 | 112.130 | -1.170 | .244 | -3.164104 | .814194 |
| [time=3] | 0^a^ | 0 |  |  |  |  |  |
| [time=1] * [Group=1] | .523984 | 1.310980 | 135.418 | .400 | .690 | -2.068658 | 3.116627 |
| [time=2] * [Group=1] | 1.460068 | 1.351538 | 112.553 | 1.080 | .282 | -1.217687 | 4.137824 |
| [time=3] * [Group=1] | 0^a^ | 0 |  |  |  |  |  |
| [time=1] * [Group=2] | 1.222290 | 1.416981 | 134.618 | .863 | .390 | -1.580134 | 4.024715 |
| [time=2] * [Group=2] | 2.256563 | 1.458715 | 112.443 | 1.547 | .125 | -.633569 | 5.146695 |
| [time=3] * [Group=2] | 0^a^ | 0 |  |  |  |  |  |
| [time=1] * [Group=3] | 0^a^ | 0 |  |  |  |  |  |
| [time=2] * [Group=3] | 0^a^ | 0 |  |  |  |  |  |
| [time=3] * [Group=3] | 0^a^ | 0 |  |  |  |  |  |
| B-IPQ_Timeline_T0 | .986762 | .501920 | 87.584 | 1.966 | .052 | -.010765 | 1.984289 |
| [Group=1] * B-IPQ_Timeline_T0 | -.455732 | .534218 | 87.584 | -.853 | .396 | -1.517449 | .605984 |
| [Group=2] * B-IPQ_Timeline_T0 | -.318045 | .533832 | 87.584 | -.596 | .553 | -1.378994 | .742903 |
| [Group=3] * B-IPQ_Timeline_T0 | 0^a^ | 0 |  |  |  |  |  |
| [time=1] * B-IPQ_Timeline_T0 | -.517365 | .556294 | 125.857 | -.930 | .354 | -1.618267 | .583536 |
| [time=2] * B-IPQ_Timeline_T0 | -.773563 | .574303 | 111.444 | -1.347 | .181 | -1.911532 | .364406 |
| [time=3] * B-IPQ_Timeline_T0 | 0^a^ | 0 |  |  |  |  |  |
| [time=1] * [Group=1] * B-IPQ_Timeline_T0 | .582369 | .593579 | 126.797 | .981 | .328 | -.592234 | 1.756972 |
| [time=2] * [Group=1] * B-IPQ_Timeline_T0 | .736115 | .613315 | 111.409 | 1.200 | .233 | -.479160 | 1.951390 |
| [time=3] * [Group=1] * B-IPQ_Timeline_T0 | 0^a^ | 0 |  |  |  |  |  |
| [time=1] * [Group=2] * B-IPQ_Timeline_T0 | .271603 | .595063 | 127.981 | .456 | .649 | -.905833 | 1.449038 |
| [time=2] * [Group=2] * B-IPQ_Timeline_T0 | .468516 | .612768 | 111.345 | .765 | .446 | -.745682 | 1.682715 |
| [time=3] * [Group=2] * B-IPQ_Timeline_T0 | 0^a^ | 0 |  |  |  |  |  |
| [time=1] * [Group=3] * B-IPQ_Timeline_T0 | 0^a^ | 0 |  |  |  |  |  |
| [time=2] * [Group=3] * B-IPQ_Timeline_T0 | 0^a^ | 0 |  |  |  |  |  |
| [time=3] * [Group=3] * B-IPQ_Timeline_T0 | 0^a^ | 0 |  |  |  |  |  |
| Notes: Group 1 = SMC, Group 2 = SUPPORT, Group 3 = EXPECT. Time 1 = 1 day pre-surgery; time 2 = 1 week post-surgery; time 3 = 6 months follow-up-assessment. Dependent Variable: B-IPQ_Timeline. a. This parameter is set to zero because it is redundant. | | | | | | | |

| *Estimates of Covariance Parameters* | | | | | | | |
| --- | --- | --- | --- | --- | --- | --- | --- |
| Parameter | | Estimate | Std. Error | Wald Z | Sig. | 95% Confidence Interval | |
|  |  |  |  |  |  | Lower Bound | Upper Bound |
| Repeated Measures | Var: [time = 1] | 3.024208 | .456962 | 6.618 | <.001 | 2.249023 | 4.066581 |
|  | Var: [time = 2] | 5.149547 | .767252 | 6.712 | <.001 | 3.845431 | 6.895934 |
|  | Var: [time = 3] | 1.275206 | 1.553037 | 6.616 | <.001 | 7.640757 | 13.817983 |
|  | ARH1, rho | .094396 | .078786 | 1.198 | .231 | -.061052 | .245373 |
| Intercept | Variance | .000000^a^ | .000000 |  |  |  |  |
| Notes: Dependent Variable: BIPQ_Timeline. Time 1 = 1 day pre-surgery; time 2 = 1 week post-surgery; time 3 = 6 months follow-up-assessment. a. This parameter is set to zero because it is redundant. | | | | | | | |

| *Estimates for Means (group)* | | | | | |
| --- | --- | --- | --- | --- | --- |
| group | Mean | Std. Error | df | 95% Confidence Interval | |
|  |  |  |  | Lower Bound | Upper Bound |
| SMC | 4.104 | .257 | 110.532 | 3.595 | 4.614 |
| SUPPORT | 3.796 | .288 | 114.786 | 3.225 | 4.366 |
| EXPECT | 4.601 | .415 | 117.885 | 3.778 | 5.423 |
| Notes: Dependent Variable: B-IPQ_Timeline. Adjusted score T0 (baseline) = 2.65. | | | | | |

| *Estimates for Means (time)* | | | | | |
| --- | --- | --- | --- | --- | --- |
| time | Mean | Std. Error | df | 95% Confidence Interval | |
|  |  |  |  | Lower Bound | Upper Bound |
| 1 | 2.802 | .204 | 87.671 | 2.396 | 3.207 |
| 2 | 4.389 | .275 | 90.300 | 3.843 | 4.934 |
| 3 | 5.311 | .417 | 87.583 | 4.481 | 6.140 |
| Notes: Dependent Variable: B-IPQ_Timeline. Time 1 = 1 day pre-surgery; time 2 = 1 week post-surgery; time 3 = 6 months follow-up-assessment. Adjusted score T0 (baseline) = 2.65. | | | | | |

| *Estimates for level of B-IPQ Timeline (average (mean))* | | | | | | |
| --- | --- | --- | --- | --- | --- | --- |
| group | time | Mean | Std. Error | df | 95% Confidence Interval | |
|  |  |  |  |  | Lower Bound | Upper Bound |
| SMC | 1 | 2.857 | .290 | 87.629 | 2.280 | 3.434 |
|  | 2 | 4.821 | .391 | 90.318 | 4.044 | 5.598 |
|  | 3 | 4.635 | .545 | 87.566 | 3.552 | 5.718 |
| SUPPORT | 1 | 2.436 | .323 | 87.684 | 1.794 | 3.078 |
|  | 2 | 4.612 | .430 | 90.329 | 3.758 | 5.466 |
|  | 3 | 4.339 | .621 | 87.577 | 3.105 | 5.574 |
| EXPECT | 1 | 3.112 | .432 | 87.682 | 2.254 | 3.970 |
|  | 2 | 3.732 | .585 | 90.275 | 2.571 | 4.894 |
|  | 3 | 6.958 | .941 | 87.584 | 5.087 | 8.828 |
| Notes: Dependent Variable: B-IPQ_Timeline. Time 1 = 1 day pre-surgery; time 2 = 1 week post-surgery; time 3 = 6 months follow-up-assessment. Adjusted score T0 (baseline) = 2.65. | | | | | | |

**Mixed Model Analysis – Results for Personal Control**

| *Model Dimension* | | | | | | | | | | | |
| --- | --- | --- | --- | --- | --- | --- | --- | --- | --- | --- | --- |
|  | | | Number of Levels | | Covariance Structure | | Number of Parameters | | Subject Variables | | Number of Subjects |
| Fixed Effects | Intercept | | 1 | |  | | 1 | |  | |  |
|  | Group | | 3 | |  | | 2 | |  | |  |
|  | time | | 3 | |  | | 2 | |  | |  |
|  | Group * time | | 9 | |  | | 4 | |  | |  |
|  | B-IPQ_Personal Control_T0 | | 1 | |  | | 1 | |  | |  |
|  | Group * B-IPQ_Personal Control_T0 | | 3 | |  | | 2 | |  | |  |
|  | time * B-IPQ_Personal Control_T0 | | 3 | |  | | 2 | |  | |  |
|  | Group * time * B-IPQ_Personal Control_T0 | | 9 | |  | | 4 | |  | |  |
| Random Effects | Intercept | | 1 | | Variance Components | | 1 | |  | |  |
| Repeated Effects | time | | 3 | | Heterogeneous First-Order Autoregressive | | 4 | | id | | 109 |
| Total | | | 36 | |  | | 23 | |  | |  |
| Notes: Dependent Variable: B-IPQ_Personal Control. | | | | | | | | | | | |
| *Type III Tests of Fixed Effects* | | | | | | | | | |  |  |
| Source | | Numerator df | | Denominator df | | F | | Sig. | |  |  |
| Intercept | | 1 | | .000 | | 4.379 | | 1.000 | |  |  |
| Group | | 2 | | 115.235 | | 7.476 | | .001 | |  |  |
| time | | 2 | | 127.768 | | 4.803 | | .010 | |  |  |
| Group * time | | 4 | | 115.496 | | 12.828 | | .001 | |  |  |
| B-IPQ_Personal Control_T0 | | 1 | | 127.549 | | 2.805 | | .028 | |  |  |
| Group * B-IPQ_Personal Control_T0 | | 2 | | 115.417 | | 4.904 | | .009 | |  |  |
| time * B-IPQ_Personal Control_T0 | | 2 | | 127.278 | | 2.211 | | .114 | |  |  |
| Group * time * B-IPQ_Personal Control_T0 | | 4 | | 127.373 | | 2.511 | | .045 | |  |  |
| Notes: Dependent Variable: B-IPQ_Personal Control. | | | | | | | | | |  |  |

| *Estimates of Fixed Effects* | | | | | | | |
| --- | --- | --- | --- | --- | --- | --- | --- |
| Parameter | Estimate | Std. Error | df | t | Sig. | 95% Confidence Interval | |
|  |  |  |  |  |  | Lower Bound | Upper Bound |
| Intercept | 5.355491 | 2.222606 | .000 | 2.410 | 1.000 | -11.733918 | 22.444900 |
| [Group=1] | -2.024660 | 1.257279 | 81.791 | -1.610 | .111 | -4.525883 | .476564 |
| [Group=2] | -1.053186 | 1.176563 | 81.831 | -.895 | .373 | -3.393817 | 1.287445 |
| [Group=3] | 0^a^ | 0 |  |  |  |  |  |
| [time=1] | .834484 | 1.071710 | 159.842 | .779 | .437 | -1.282053 | 2.951022 |
| [time=2] | .352604 | 1.024174 | 124.289 | .344 | .731 | -1.674476 | 2.379684 |
| [time=3] | 0^a^ | 0 |  |  |  |  |  |
| [time=1] * [Group=1] | -.145361 | .176156 | 81.885 | -.825 | .412 | -.495799 | .205076 |
| [time=2] * [Group=1] | -1.866199 | 1.636721 | 166.464 | -1.140 | .256 | -5.097605 | 1.365207 |
| [time=3] * [Group=1] | .792675 | 1.588236 | 120.008 | .499 | .619 | -2.351920 | 3.937270 |
| [time=1] * [Group=2] | 0^a^ | 0 |  |  |  |  |  |
| [time=2] * [Group=2] | -3.311108 | 1.509817 | 163.611 | -2.193 | .030 | -6.292346 | -.329870 |
| [time=3] * [Group=2] | .877377 | 1.480899 | 124.321 | .592 | .555 | -2.053662 | 3.808416 |
| [time=1] * [Group=3] | 0^a^ | 0 |  |  |  |  |  |
| [time=2] * [Group=3] | 0^a^ | 0 |  |  |  |  |  |
| [time=3] * [Group=3] | 0^a^ | 0 |  |  |  |  |  |
| B-IPQ_Personal Control_T0 | 0^a^ | 0 |  |  |  |  |  |
| [Group=1] * B-IPQ_Personal Control_T0 | .370263 | .247557 | 81.803 | 1.496 | .139 | -.122225 | .862751 |
| [Group=2] * B-IPQ_Personal Control_T0 | .307824 | .228004 | 81.828 | 1.350 | .181 | -.145763 | .761411 |
| [Group=3] * B-IPQ_Personal Control_T0 | 0^a^ | 0 |  |  |  |  |  |
| [time=1] * B-IPQ_Personal Control_T0 | .066567 | .222809 | 160.375 | .299 | .766 | -.373451 | .506586 |
| [time=2] * B-IPQ_Personal Control_T0 | .261712 | .215395 | 125.878 | 1.215 | .227 | -.164553 | .687976 |
| [time=3] * B-IPQ_Personal Control_T0 | 0^a^ | 0 |  |  |  |  |  |
| [time=1] * [Group=1] * B-IPQ_Personal Control_T0 | .054533 | .320518 | 165.426 | .170 | .865 | -.578301 | .687367 |
| [time=2] * [Group=1] * B-IPQ_Personal Control_T0 | -.266417 | .312240 | 121.562 | -.853 | .395 | -.884550 | .351716 |
| [time=3] * [Group=1] * B-IPQ_Personal Control_T0 | 0^a^ | 0 |  |  |  |  |  |
| [time=1] * [Group=2] * B-IPQ_Personal Control_T0 | .481905 | .294323 | 164.813 | 1.637 | .103 | -.099224 | 1.063034 |
| [time=2] * [Group=2] * B-IPQ_Personal Control_T0 | -.306406 | .287338 | 124.649 | -1.066 | .288 | -.875099 | .262287 |
| [time=3] * [Group=2] * B-IPQ_Personal Control_T0 | 0^a^ | 0 |  |  |  |  |  |
| [time=1] * [Group=3] * B-IPQ_Personal Control_T0 | 0^a^ | 0 |  |  |  |  |  |
| [time=2] * [Group=3] * B-IPQ_Personal Control_T0 | 0^a^ | 0 |  |  |  |  |  |
| [time=3] * [Group=3] * B-IPQ_Personal Control_T0 | 0^a^ | 0 |  |  |  |  |  |
| Notes: Group 1 = SMC, Group 2 = SUPPORT, Group 3 = EXPECT. Time 1 = 1 day pre-surgery; time 2 = 1 week post-surgery; time 3 = 6 months follow-up-assessment. Dependent Variable: B-IPQ_Personal Control. a. This parameter is set to zero because it is redundant. | | | | | | | |

| *Estimates of Covariance Parameters* | | | | | | | |
| --- | --- | --- | --- | --- | --- | --- | --- |
| Parameter | | Estimate | Std. Error | Wald Z | Sig. | 95% Confidence Interval | |
|  |  |  |  |  |  | Lower Bound | Upper Bound |
| Repeated Measures | Var: [time = 1] | 5.214818 | .755086 | 6.906 | <.001 | 3.926345 | 6.926117 |
|  | Var: [time = 2] | 4.737167 | .714326 | 6.632 | <.001 | 3.525044 | 6.366091 |
|  | Var: [time = 3] | 5.874624 | .919409 | 6.390 | <.001 | 4.32776 | 7.983577 |
|  | ARH1, rho | .075511 | .081807 | .923 | .356 | -.085395 | .232578 |
| Intercept | Variance | 4.218957 | 202918131.3 | .000 | 1.000 | .000000 |  |
| Notes: Dependent Variable: B-IPQ_Personal Control. Time 1 = 1 day pre-surgery; time 2 = 1 week post-surgery; time 3 = 6 months follow-up-assessment. | | | | | | | |

| *Estimates for Means (group)* | | | | | |
| --- | --- | --- | --- | --- | --- |
| group | Mean | Std. Error | df | 95% Confidence Interval | |
|  |  |  |  | Lower Bound | Upper Bound |
| SMC | 4.532 | 2.068 | .000 | -5.702 | 14.765 |
| SUPPORT | 5.344 | 2.070 | .000 | -4.374 | 15.062 |
| EXPECT | 5.593 | 2.070 | .000 | -4.124 | 15.309 |
| Notes: Dependent Variable: B-IPQ_Personal Control. Adjusted score T0 (baseline) = 4.41. | | | | | |

| *Estimates for Means (time)* | | | | | |
| --- | --- | --- | --- | --- | --- |
| time | Mean | Std. Error | df | 95% Confidence Interval | |
|  |  |  |  | Lower Bound | Upper Bound |
| 1 | 4.876 | 2.067 | .000 | -5.597 | 15.349 |
| 2 | 5.907 | 2.067 | .000 | -4.570 | 16.383 |
| 3 | 4.685 | 2.071 | .000 | -4.899 | 14.269 |
| Notes: Dependent Variable: B-IPQ_Personal Control. Time 1 = 1 day pre-surgery; time 2 = 1 week post-surgery; time 3 = 6 months follow-up-assessment. Adjusted score T0 (baseline) = 4.41. | | | | | |

| *Estimates for level of B-IPQ Personal Control (average (mean))* | | | | | | |
| --- | --- | --- | --- | --- | --- | --- |
| group | time | Mean | Std. Error | df | 95% Confidence Interval | |
|  |  |  |  |  | Lower Bound | Upper Bound |
| SMC | 1 | 3.825 | 2.090 | .000 | -6.212 | 13.862 |
|  | 2 | 5.447 | 2.089 | .000 | -4.791 | 15.685 |
|  | 3 | 4.323 | 2.097 | .000 | -4.349 | 12.994 |
| SUPPORT | 1 | 4.961 | 2.093 | .000 | -4.342 | 14.264 |
|  | 2 | 6.052 | 2.098 | .000 | -4.486 | 16.589 |
|  | 3 | 5.019 | 2.103 | .000 | -4.404 | 14.442 |
| EXPECT | 1 | 5.843 | 2.094 | .000 | -3.384 | 15.069 |
|  | 2 | 6.221 | 2.090 | .000 | -3.833 | 16.275 |
|  | 3 | 4.714 | 2.112 | .000 | -5.028 | 14.457 |
| Notes: Dependent Variable: B-IPQ_Personal Control. Time 1 = 1 day pre-surgery; time 2 = 1 week post-surgery; time 3 = 6 months follow-up-assessment. Adjusted score T0 (baseline) = 4.41. | | | | | | |

**Mixed Model Analysis – Results for Treatment Control**

| *Model Dimension* | | | | | | |
| --- | --- | --- | --- | --- | --- | --- |
|  | | Number of Levels | Covariance Structure | Number of Parameters | Subject Variables | Number of Subjects |
| Fixed Effects | Intercept | 1 |  | 1 |  |  |
|  | Group | 3 |  | 2 |  |  |
|  | time | 3 |  | 2 |  |  |
|  | Group * time | 9 |  | 4 |  |  |
|  | B-IPQ_Treatment Control_T0 | 1 |  | 1 |  |  |
|  | Group * B-IPQ_Treatment Control_T0 | 3 |  | 2 |  |  |
|  | time * B-IPQ_Treatment Control_T0 | 3 |  | 2 |  |  |
|  | Group * time * B-IPQ_Treatment Control_T0 | 9 |  | 4 |  |  |
| Random Effects | Intercept | 1 | Variance Components | 1 |  |  |
| Repeated Effects | time | 3 | Compound Symmetry | 4 | id | 105 |
| Total | | 36 |  | 23 |  |  |
| Notes: Dependent Variable: B-IPQ_Treatment Control. | | | | | | |

| *Type III Tests of Fixed Effects* | | | | |
| --- | --- | --- | --- | --- |
| Source | Numerator df | Denominator df | F | Sig. |
| Intercept | 1 | 110.954 | 19.590 | <.001 |
| Group | 2 | 105.818 | .582 | .561 |
| time | 2 | 119.025 | 2.996 | .054 |
| Group * time | 4 | 109.307 | 13.318 | <.001 |
| B-IPQ_Treatment Control_T0 | 1 | 117.891 | 1.654 | .165 |
| Group * B-IPQ_Treatment Control_T0 | 2 | 104.995 | .399 | .672 |
| time * B-IPQ_Treatment Control_T0 | 2 | 118.294 | 3.306 | .040 |
| Group * time * B-IPQ_Treatment Control_T0 | 4 | 117.259 | 1.308 | .271 |
| Notes: Dependent Variable: B-IPQ_Treatment Control. | | | | |

| *Estimates of Fixed Effects* | | | | | | | |
| --- | --- | --- | --- | --- | --- | --- | --- |
| Parameter | Estimate | Std. Error | df | t | Sig. | 95% Confidence Interval | |
|  |  |  |  |  |  | Lower Bound | Upper Bound |
| Intercept | -1.903260 | 4.771472 | 88.851 | -.399 | .691 | -11.384291 | 7.577771 |
| [Group=1] | 4.814512 | 5.298082 | 88.579 | .909 | .366 | -5.713354 | 15.342378 |
| [Group=2] | 1.394874 | 5.270699 | 88.575 | 1.972 | .052 | -.078586 | 2.868333 |
| [Group=3] | 0^a^ | 0 |  |  |  |  |  |
| [time=1] | 6.554965 | 5.636962 | 151.886 | 1.163 | .247 | -4.582013 | 17.691943 |
| [time=2] | 8.430657 | 4.808877 | 102.137 | 1.753 | .083 | -1.107574 | 17.968888 |
| [time=3] | 0^a^ | 0 |  |  |  |  |  |
| [time=1] * [Group=1] | 1.032719 | .503628 | 88.787 | 2.051 | .043 | .031988 | 2.033450 |
| [time=2] * [Group=1] | -5.115846 | 6.283050 | 153.505 | -.814 | .417 | -17.528254 | 7.296562 |
| [time=3] * [Group=1] | -4.116142 | 5.348030 | 102.831 | -.770 | .443 | -14.722906 | 6.490621 |
| [time=1] * [Group=2] | 0^a^ | 0 |  |  |  |  |  |
| [time=2] * [Group=2] | -12.296682 | 6.189199 | 151.100 | -1.987 | .049 | -24.525230 | -.068135 |
| [time=3] * [Group=2] | -1.145565 | 5.317673 | 102.663 | -1.908 | .059 | -2.692328 | .401197 |
| [time=1] * [Group=3] | 0^a^ | 0 |  |  |  |  |  |
| [time=2] * [Group=3] | 0^a^ | 0 |  |  |  |  |  |
| [time=3] * [Group=3] | 0^a^ | 0 |  |  |  |  |  |
| B-IPQ_Treatment Control_T0 | 0^a^ | 0 |  |  |  |  |  |
| [Group=1] * B-IPQ_Treatment Control_T0 | -.491621 | .567567 | 88.514 | -.866 | .389 | -1.619450 | .636209 |
| [Group=2] * B-IPQ_Treatment Control_T0 | -1.001595 | .563967 | 88.476 | -1.776 | .079 | -2.122276 | .119086 |
| [Group=3] * B-IPQ_Treatment Control_T0 | 0^a^ | 0 |  |  |  |  |  |
| [time=1] * B-IPQ_Treatment Control_T0 | -.563922 | .594321 | 151.769 | -.949 | .344 | -1.738132 | .610288 |
| [time=2] * B-IPQ_Treatment Control_T0 | -.824957 | .507644 | 102.260 | -1.625 | .107 | -1.831835 | .181922 |
| [time=3] * B-IPQ_Treatment Control_T0 | 0^a^ | 0 |  |  |  |  |  |
| [time=1] * [Group=1] * B-IPQ_Treatment Control_T0 | .521437 | .671242 | 152.959 | .777 | .438 | -.804666 | 1.847540 |
| [time=2] * [Group=1] * B-IPQ_Treatment Control_T0 | .372637 | .573143 | 102.993 | .650 | .517 | -.764057 | 1.509332 |
| [time=3] * [Group=1] * B-IPQ_Treatment Control_T0 | 0^a^ | 0 |  |  |  |  |  |
| [time=1] * [Group=2] * B-IPQ_Treatment Control_T0 | 1.182006 | .660867 | 150.739 | 1.789 | .076 | -.123752 | 2.487764 |
| [time=2] * [Group=2] * B-IPQ_Treatment Control_T0 | 1.007209 | .569352 | 102.899 | 1.769 | .080 | -.121981 | 2.136398 |
| [time=3] * [Group=2] * B-IPQ_Treatment Control_T0 | 0^a^ | 0 |  |  |  |  |  |
| [time=1] * [Group=3] * B-IPQ_Treatment Control_T0 | 0^a^ | 0 |  |  |  |  |  |
| [time=2] * [Group=3] * B-IPQ_Treatment Control_T0 | 0^a^ | 0 |  |  |  |  |  |
| [time=3] * [Group=3] * B-IPQ_Treatment Control_T0 | 0^a^ | 0 |  |  |  |  |  |
| Notes: Group 1 = SMC, Group 2 = SUPPORT, Group 3 = EXPECT. Time 1 = 1 day pre-surgery; time 2 = 1 week post-surgery; time 3 = 6 months follow-up-assessment. Dependent Variable: B-IPQ_Treatment Control. a. This parameter is set to zero because it is redundant. | | | | | | | |

| *Estimates of Covariance Parameters* | | | | | | | |
| --- | --- | --- | --- | --- | --- | --- | --- |
| Parameter | | Estimate | Std. Error | Wald Z | Sig. | 95% Confidence Interval | |
|  |  |  |  |  |  | Lower Bound | Upper Bound |
| Repeated Measures | Var: [time = 1] | 2.019320 | .305933 | 6.601 | <.001 | 1.500533 | 2.717470 |
|  | Var: [time = 2] | 2.441620 | .359713 | 6.788 | <.001 | 1.829256 | 3.258981 |
|  | Var: [time = 3] | 4.659247 | .709992 | 6.562 | <.001 | 3.456263 | 6.280942 |
|  | ARH1, rho | .346440 | .076236 | 4.544 | <.001 | .189283 | .486291 |
| Intercept | Variance | .000000^a^ | .000000 |  |  |  |  |
| Notes: Dependent Variable: B-IPQ_Treatment Control. Time 1 = 1 day pre-surgery; time 2 = 1 week post-surgery; time 3 = 6 months follow-up-assessment. a. This parameter is set to zero because it is redundant. | | | | | | | |

| *Estimates for Means (group)* | | | | | |
| --- | --- | --- | --- | --- | --- |
| group | Mean | Std. Error | df | 95% Confidence Interval | |
|  |  |  |  | Lower Bound | Upper Bound |
| SMC | 8.207 | .212 | 96.437 | 7.787 | 8.627 |
| SUPPORT | 8.681 | .227 | 97.752 | 8.230 | 9.132 |
| EXPECT | 8.208 | .247 | 106.971 | 7.718 | 8.697 |
| Notes: Dependent Variable: B-IPQ_Treatment Control. Adjusted score T0 (baseline) = 8.98. | | | | | |

| *Estimates for Means (time)* | | | | | |
| --- | --- | --- | --- | --- | --- |
| time | Mean | Std. Error | df | 95% Confidence Interval | |
|  |  |  |  | Lower Bound | Upper Bound |
| 1 | 8.756 | .158 | 89.395 | 8.443 | 9.069 |
| 2 | 8.369 | .163 | 93.565 | 8.047 | 8.692 |
| 3 | 7.970 | .241 | 87.901 | 7.490 | 8.450 |
| Notes: Dependent Variable: B-IPQ_Treatment Control. Time 1 = 1 day pre-surgery; time 2 = 1 week post-surgery; time 3 = 6 months follow-up-assessment. Adjusted score T0 (baseline) = 8.98. | | | | | |

| *Estimates for level of B-IPQ Treatment Control (average (mean))* | | | | | | |
| --- | --- | --- | --- | --- | --- | --- |
| group | time | Mean | Std. Error | df | 95% Confidence Interval | |
|  |  |  |  |  | Lower Bound | Upper Bound |
| SMC | 1 | 8.827 | .243 | 87.815 | 8.344 | 9.310 |
|  | 2 | 8.023 | .268 | 94.452 | 7.491 | 8.555 |
|  | 3 | 7.770 | .381 | 87.506 | 7.011 | 8.528 |
| SUPPORT | 1 | 8.579 | .254 | 87.652 | 8.075 | 9.083 |
|  | 2 | 8.693 | .284 | 94.205 | 8.128 | 9.257 |
|  | 3 | 8.771 | .419 | 87.804 | 7.939 | 9.603 |
| EXPECT | 1 | 8.861 | .316 | 90.987 | 8.233 | 9.489 |
|  | 2 | 8.393 | .292 | 92.146 | 7.814 | 8.972 |
|  | 3 | 7.369 | .451 | 88.227 | 6.473 | 8.266 |
| Notes: Dependent Variable: B-IPQ_Treatment Control. Time 1 = 1 day pre-surgery; time 2 = 1 week post-surgery; time 3 = 6 months follow-up-assessment. Adjusted score T0 (baseline) = 8.98. | | | | | | |

**Mixed Model Analysis – Results for Identity**

| *Model Dimension* | | | | | | |
| --- | --- | --- | --- | --- | --- | --- |
|  | | Number of Levels | Covariance Structure | Number of Parameters | Subject Variables | Number of Subjects |
| Fixed Effects | Intercept | 1 |  | 1 |  |  |
|  | Group | 3 |  | 2 |  |  |
|  | time | 3 |  | 2 |  |  |
|  | Group * time | 9 |  | 4 |  |  |
|  | B-IPQ_Identity_T0 | 1 |  | 1 |  |  |
|  | Group * B-IPQ_Identity_T0 | 3 |  | 2 |  |  |
|  | time * B-IPQ_Identity_T0 | 3 |  | 2 |  |  |
|  | Group * time * B-IPQ_Identity_T0 | 9 |  | 4 |  |  |
| Random Effects | Intercept | 1 | Variance Components | 1 |  |  |
| Repeated Effects | time | 3 | Heterogeneous First-Order Autoregressive | 4 | id | 111 |
| Total | | 36 |  | 23 |  |  |
| Notes: Dependent Variable: B-IPQ_Identity. | | | | | | |

| *Type III Tests of Fixed Effects* | | | | |
| --- | --- | --- | --- | --- |
| Source | Numerator df | Denominator df | F | Sig. |
| Intercept | 1 | .000 | .436 | 1.000 |
| Group | 2 | 118.830 | .687 | .505 |
| time | 2 | 152.641 | 16.699 | <.001 |
| Group * time | 4 | 115.435 | 52.061 | <.001 |
| B-IPQ_Identity_T0 | 1 | 152.844 | 2.519 | .044 |
| Group * B-IPQ_Identity_T0 | 2 | 115.402 | .431 | .651 |
| time * B-IPQ_Identity_T0 | 2 | 152.119 | 10.526 | <.001 |
| Group * time * B-IPQ_Identity_T0 | 4 | 152.141 | 2.216 | .070 |
| Notes: Dependent Variable: B-IPQ_Identity. | | | | |

| *Estimates of Fixed Effects* | | | | | | | |
| --- | --- | --- | --- | --- | --- | --- | --- |
| Parameter | Estimate | Std. Error | df | t | Sig. | 95% Confidence Interval | |
|  |  |  |  |  |  | Lower Bound | Upper Bound |
| Intercept | .165196 | 3.785667 | .000 | .044 | 1.000 | -10.429985 | 10.760378 |
| [Group=1] | 2.137546 | 1.116027 | 92.793 | 1.915 | .059 | -.078727 | 4.353818 |
| [Group=2] | 1.869069 | 1.154485 | 92.786 | 1.619 | .109 | -.423579 | 4.161718 |
| [Group=3] | 0 | 0 |  |  |  |  |  |
| [time=1] | 2.419322 | 1.070446 | 176.025 | 2.260 | .025 | .306762 | 4.531881 |
| [time=2] | 3.782511 | 1.127387 | 124.765 | 3.355 | .001 | 1.551231 | 6.013791 |
| [time=3] | 0 | 0 |  |  |  |  |  |
| [time=1] * [Group=1] | .458347 | .151078 | 92.802 | 3.034 | .003 | .158327 | .758366 |
| [time=2] * [Group=1] | -3.697521 | 1.413754 | 176.385 | -2.615 | .010 | -6.487570 | -.907472 |
| [time=3] * [Group=1] | -.835197 | 1.518526 | 126.988 | -.550 | .583 | -3.840089 | 2.169695 |
| [time=1] * [Group=2] | 0 | 0 |  |  |  |  |  |
| [time=2] * [Group=2] | -3.754037 | 1.440386 | 172.858 | -2.606 | .010 | -6.597046 | -.911029 |
| [time=3] * [Group=2] | -1.851312 | 1.549826 | 126.122 | -1.195 | .235 | -4.918343 | 1.215719 |
| [time=1] * [Group=3] | 0 | 0 |  |  |  |  |  |
| [time=2] * [Group=3] | 0 | 0 |  |  |  |  |  |
| [time=3] * [Group=3] | 0 | 0 |  |  |  |  |  |
| B-IPQ_Identity_T0 | 0 | 0 |  |  |  |  |  |
| [Group=1] * B-IPQ_Identity_T0 | -.259677 | .210634 | 92.807 | -1.233 | .221 | -.677967 | .158613 |
| [Group=2] * B-IPQ_Identity_T0 | -.319679 | .206991 | 92.798 | -1.544 | .126 | -.730733 | .091375 |
| [Group=3] * B-IPQ_Identity_T0 | 0 | 0 |  |  |  |  |  |
| [time=1] * B-IPQ_Identity_T0 | -.025329 | .192117 | 177.253 | -.132 | .895 | -.404460 | .353801 |
| [time=2] * B-IPQ_Identity_T0 | -.114084 | .203381 | 123.056 | -.561 | .576 | -.516663 | .288494 |
| [time=3] * B-IPQ_Identity_T0 | 0 | 0 |  |  |  |  |  |
| [time=1] * [Group=1] * B-IPQ_Identity_T0 | .466678 | .267723 | 177.171 | 1.743 | .083 | -.061658 | .995014 |
| [time=2] * [Group=1] * B-IPQ_Identity_T0 | -.017239 | .288090 | 124.958 | -.060 | .952 | -.587407 | .552929 |
| [time=3] * [Group=1] * B-IPQ_Identity_T0 | 0 | 0 |  |  |  |  |  |
| [time=1] * [Group=2] * B-IPQ_Identity_T0 | .648768 | .259419 | 173.994 | 2.501 | .013 | .136756 | 1.160781 |
| [time=2] * [Group=2] * B-IPQ_Identity_T0 | .078854 | .279001 | 124.214 | .283 | .778 | -.473358 | .631065 |
| [time=3] * [Group=2] * B-IPQ_Identity_T0 | 0 | 0 |  |  |  |  |  |
| [time=1] * [Group=3] * B-IPQ_Identity_T0 | 0 | 0 |  |  |  |  |  |
| [time=2] * [Group=3] * B-IPQ_Identity_T0 | 0 | 0 |  |  |  |  |  |
| [time=3] * [Group=3] * B-IPQ_Identity_T0 | 0 | 0 |  |  |  |  |  |
| Notes: Group 1 = SMC, Group 2 = SUPPORT, Group 3 = EXPECT. Time 1 = 1 day pre-surgery; time 2 = 1 week post-surgery; time 3 = 6 months follow-up-assessment. Dependent Variable: B-IPQ_Identity. a. This parameter is set to zero because it is redundant. | | | | | | | |

| *Estimates of Covariance Parameters* | | | | | | | |
| --- | --- | --- | --- | --- | --- | --- | --- |
| Parameter | | Estimate | Std. Error | Wald Z | Sig. | 95% Confidence Interval | |
|  |  |  |  |  |  | Lower Bound | Upper Bound |
| Repeated Measures | Var: [time = 1] | 3.115743 | .449994 | 6.924 | <.001 | 2.347611 | 4.135205 |
|  | Var: [time = 2] | 5.540844 | .803651 | 6.895 | <.001 | 4.169813 | 7.362669 |
|  | Var: [time = 3] | 5.047944 | .740938 | 6.813 | <.001 | 3.785951 | 6.730605 |
|  | ARH1, rho | .093233 | .075829 | 1.230 | .219 | -.056361 | .238733 |
| Intercept | Variance | 13.614985 | 1073741824 | .000 | .000 | .000 |  |
| Notes: Dependent Variable: B-IPQ_Identity. Time 1 = 1 day pre-surgery; time 2 = 1 week post-surgery; time 3 = 6 months follow-up-assessment. | | | | | | | |

| *Estimates for Means (group)* | | | | | |
| --- | --- | --- | --- | --- | --- |
| group | Mean | Std. Error | df | 95% Confidence Interval | |
|  |  |  |  | Lower Bound | Upper Bound |
| SMC | 4.248 | 3.697 | .000 | -4.968 | 13.464 |
| SUPPORT | 3.773 | 3.697 | .000 | -5.349 | 12.894 |
| EXPECT | 4.127 | 3.697 | .000 | -5.072 | 13.326 |
| Notes: Dependent Variable: B-IPQ_Identity. Adjusted score T0 (baseline) = 4.6. | | | | | |

| *Estimates for Means (time)* | | | | | |
| --- | --- | --- | --- | --- | --- |
| time | Mean | Std. Error | df | 95% Confidence Interval | |
|  |  |  |  | Lower Bound | Upper Bound |
| 1 | 4.250 | 3.694 | .000 | -5.254 | 13.754 |
| 2 | 5.177 | 3.697 | .000 | -3.946 | 14.301 |
| 3 | 2.721 | 3.697 | .000 | -6.450 | 11.891 |
| Notes: Dependent Variable: B-IPQ_Identity. Time 1 = 1 day pre-surgery; time 2 = 1 week post-surgery; time 3 = 6 months follow-up-assessment. Adjusted score T0 (baseline) = 4.6. | | | | | |

| *Estimates for level of B-IPQ Identity (average (mean))* | | | | | | |
| --- | --- | --- | --- | --- | --- | --- |
| group | time | Mean | Std. Error | df | 95% Confidence Interval | |
|  |  |  |  |  | Lower Bound | Upper Bound |
| SMC | 1 | 3.968 | 3.702 | .000 | -6.761 | 14.697 |
|  | 2 | 5.560 | 3.712 | .000 | -3.971 | 15.090 |
|  | 3 | 3.216 | 3.710 | .000 | -6.524 | 12.957 |
| SUPPORT | 1 | 4.205 | 3.703 | .000 | -6.415 | 14.824 |
|  | 2 | 4.441 | 3.715 | .000 | -4.728 | 13.610 |
|  | 3 | 2.672 | 3.713 | .000 | -6.736 | 12.080 |
| EXPECT | 1 | 4.576 | 3.702 | .000 | -6.068 | 15.220 |
|  | 2 | 5.531 | 3.711 | .000 | -4.087 | 15.149 |
|  | 3 | 2.273 | 3.711 | .000 | -7.327 | 11.874 |
| Notes: Dependent Variable: B-IPQ_Identity. Time 1 = 1 day pre-surgery; time 2 = 1 week post-surgery; time 3 = 6 months follow-up-assessment. Adjusted score T0 (baseline) = 4.6. | | | | | | |

**Mixed Model Analysis – Results for Concern**

| *Model Dimension* | | | | | | |
| --- | --- | --- | --- | --- | --- | --- |
|  | | Number of Levels | Covariance Structure | Number of Parameters | Subject Variables | Number of Subjects |
| Fixed Effects | Intercept | 1 |  | 1 |  |  |
|  | Group | 3 |  | 2 |  |  |
|  | time | 3 |  | 2 |  |  |
|  | Group * time | 9 |  | 4 |  |  |
|  | B-IPQ_Concern_T0 | 1 |  | 1 |  |  |
|  | Group * B-IPQ_Concern_T0 | 3 |  | 2 |  |  |
|  | time * B-IPQ_Concern_T0 | 3 |  | 2 |  |  |
|  | Group * time * B-IPQ_Concern_T0 | 9 |  | 4 |  |  |
| Random Effects | Intercept | 1 | Variance Components | 1 |  |  |
| Repeated Effects | time | 3 | Heterogeneous First-Order Autoregressive | 4 | id | 111 |
| Total | | 36 |  | 23 |  |  |
| Notes: Dependent Variable: B-IPQ_Concern. | | | | | | |

| *Type III Tests of Fixed Effects* | | | | |
| --- | --- | --- | --- | --- |
| Source | Numerator df | Denominator df | F | Sig. |
| Intercept | 1 | 5767.017 | 3.750 | .053 |
| Group | 2 | 107.511 | .854 | .428 |
| time | 2 | 152.531 | 3.598 | .030 |
| Group * time | 4 | 152.753 | 2.763 | .030 |
| B-IPQ_Concern_T0 | 1 | 105.413 | 73.950 | <.001 |
| Group * B-IPQ_Concern_T0 | 2 | 105.249 | .662 | .518 |
| time * B-IPQ_Concern_T0 | 2 | 150.596 | 4.114 | .018 |
| Group * time * B-IPQ_Concern_T0 | 4 | 150.618 | 2.492 | .046 |
| Notes: Dependent Variable: B-IPQ_Concern. | | | | |

| *Estimates of Fixed Effects* | | | | | | | |
| --- | --- | --- | --- | --- | --- | --- | --- |
| Parameter | Estimate | Std. Error | df | t | Sig. | 95% Confidence Interval | |
|  |  |  |  |  |  | Lower Bound | Upper Bound |
| Intercept | .424962 | 1.209557 | 57.260 | .351 | .725 | -1.950768 | 2.800693 |
| [Group=1] | 1.774265 | 1.151213 | 95.762 | 1.541 | .127 | -.510948 | 4.059477 |
| [Group=2] | -.223863 | 1.339832 | 96.318 | -.167 | .868 | -2.883297 | 2.435571 |
| [Group=3] | 0^a^ | 0 |  |  |  |  |  |
| [time=1] | 3.502058 | 1.042399 | 186.730 | 3.360 | .001 | 1.445666 | 5.558450 |
| [time=2] | 1.875554 | .968452 | 136.525 | 1.937 | .055 | -.039552 | 3.790660 |
| [time=3] | 0^a^ | 0 |  |  |  |  |  |
| [time=1] * [Group=1] | -4.499388 | 1.551927 | 186.804 | -2.899 | .004 | -7.560942 | -1.437833 |
| [time=2] * [Group=1] | -.312243 | 1.472785 | 135.593 | -.212 | .832 | -3.224844 | 2.600358 |
| [time=3] * [Group=1] | 0^a^ | 0 |  |  |  |  |  |
| [time=1] * [Group=2] | -2.440951 | 1.732544 | 184.903 | -1.409 | .161 | -5.859046 | .977144 |
| [time=2] * [Group=2] | .068320 | 1.687922 | 138.887 | .040 | .968 | -3.269026 | 3.405665 |
| [time=3] * [Group=2] | 0^a^ | 0 |  |  |  |  |  |
| [time=1] * [Group=3] | 0^a^ | 0 |  |  |  |  |  |
| [time=2] * [Group=3] | 0^a^ | 0 |  |  |  |  |  |
| [time=3] * [Group=3] | 0^a^ | 0 |  |  |  |  |  |
| B-IPQ_Concern_T0 | .441902 | .110797 | 95.794 | 3.988 | <.001 | .221966 | .661838 |
| [Group=1] * B-IPQ_Concern_T0 | -.247578 | .172358 | 95.807 | -1.436 | .154 | -.589714 | .094558 |
| [Group=2] * B-IPQ_Concern_T0 | .050071 | .183420 | 96.176 | .273 | .785 | -.314007 | .414149 |
| [Group=3] * B-IPQ_Concern_T0 | 0^a^ | 0 |  |  |  |  |  |
| [time=1] * B-IPQ_Concern_T0 | -.095982 | .149246 | 186.803 | -.643 | .521 | -.390407 | .198444 |
| [time=2] * B-IPQ_Concern_T0 | -.044680 | .139269 | 135.247 | -.321 | .749 | -.320108 | .230748 |
| [time=3] * B-IPQ_Concern_T0 | 0^a^ | 0 |  |  |  |  |  |
| [time=1] * [Group=1] * B-IPQ_Concern_T0 | .669182 | .230575 | 186.629 | 2.902 | .004 | .214313 | 1.124052 |
| [time=2] * [Group=1] * B-IPQ_Concern_T0 | .171627 | .218733 | 135.825 | .785 | .434 | -.260936 | .604189 |
| [time=3] * [Group=1] * B-IPQ_Concern_T0 | 0^a^ | 0 |  |  |  |  |  |
| [time=1] * [Group=2] * B-IPQ_Concern_T0 | .325903 | .239417 | 185.603 | 1.361 | .175 | -.146426 | .798232 |
| [time=2] * [Group=2] * B-IPQ_Concern_T0 | -.044598 | .229992 | 137.655 | -.194 | .847 | -.499372 | .410176 |
| [time=3] * [Group=2] * B-IPQ_Concern_T0 | 0^a^ | 0 |  |  |  |  |  |
| [time=1] * [Group=3] * B-IPQ_Concern_T0 | 0^a^ | 0 |  |  |  |  |  |
| [time=2] * [Group=3] * B-IPQ_Concern_T0 | 0^a^ | 0 |  |  |  |  |  |
| [time=3] * [Group=3] * B-IPQ_Concern_T0 | 0^a^ | 0 |  |  |  |  |  |
| Notes: Group 1 = SMC, Group 2 = SUPPORT, Group 3 = EXPECT. Time 1 = 1 day pre-surgery; time 2 = 1 week post-surgery; time 3 = 6 months follow-up-assessment. Dependent Variable: B-IPQ_Concern. a. This parameter is set to zero because it is redundant. | | | | | | | |

| *Estimates of Covariance Parameters* | | | | | | | |
| --- | --- | --- | --- | --- | --- | --- | --- |
| Parameter | | Estimate | Std. Error | Wald Z | Sig. | 95% Confidence Interval | |
|  |  |  |  |  |  | Lower Bound | Upper Bound |
| Repeated Measures | Var: [time = 1] | 5.436759 | .777750 | 6.991 | <.001 | 4.107451 | 7.196277 |
|  | Var: [time = 2] | 7.349328 | 1.063169 | 6.913 | <.001 | 5.534916 | 9.758526 |
|  | Var: [time = 3] | 5.612161 | .813503 | 6.899 | <.001 | 4.224209 | 7.456155 |
|  | ARH1, rho | .298470 | .071628 | 4.167 | <.001 | .152522 | .431677 |
| Intercept | Variance | .862766^a^ | .000000 |  |  |  |  |
| Notes: Dependent Variable: B-IPQ_Concern. Time 1 = 1 day pre-surgery; time 2 = 1 week post-surgery; time 3 = 6 months follow-up-assessment. a. This parameter is set to zero because it is redundant. | | | | | | | |

| *Estimates for Means (group)* | | | | | |
| --- | --- | --- | --- | --- | --- |
| group | Mean | Std. Error | df | 95% Confidence Interval | |
|  |  |  |  | Lower Bound | Upper Bound |
| SMC | 5.020 | .972 | 13955.539 | 3.114 | 6.926 |
| SUPPORT | 4.519 | .979 | 10850.544 | 2.600 | 6.438 |
| EXPECT | 4.649 | .974 | 12724.498 | 2.740 | 6.558 |
| Notes: Dependent Variable: B-IPQ_Concern. Adjusted score T0 (baseline) = 6.15. | | | | | |

| *Estimates for Means (time)* | | | | | |
| --- | --- | --- | --- | --- | --- |
| time | Mean | Std. Error | df | 95% Confidence Interval | |
|  |  |  |  | Lower Bound | Upper Bound |
| 1 | 5.896 | .957 | 29322.967 | 4.020 | 7.771 |
| 2 | 5.036 | .968 | 15911.872 | 3.140 | 6.933 |
| 3 | 3.256 | .960 | 24031.549 | 1.375 | 5.137 |
| Notes: Dependent Variable: B-IPQ_Concern. Time 1 = 1 day pre-surgery; time 2 = 1 week post-surgery; time 3 = 6 months follow-up-assessment. Adjusted score T0 (baseline) = 6.15. | | | | | |

| *Estimates for level of B-IPQ Concern (average (mean))* | | | | | | |
| --- | --- | --- | --- | --- | --- | --- |
| group | time | Mean | Std. Error | df | 95% Confidence Interval | |
|  |  |  |  |  | Lower Bound | Upper Bound |
| SMC | 1 | 5.926 | 1.007 | 4412.779 | 3.951 | 7.900 |
|  | 2 | 5.740 | 1.035 | 2591.965 | 3.711 | 7.769 |
|  | 3 | 3.395 | 1.013 | 3819.875 | 1.410 | 5.380 |
| SUPPORT | 1 | 5.705 | 1.015 | 3750.542 | 3.716 | 7.694 |
|  | 2 | 4.623 | 1.053 | 1999.222 | 2.558 | 6.689 |
|  | 3 | 3.229 | 1.027 | 2903.001 | 1.216 | 5.242 |
| EXPECT | 1 | 6.056 | 1.011 | 4050.819 | 4.074 | 8.038 |
|  | 2 | 4.745 | 1.034 | 2596.100 | 2.719 | 6.772 |
|  | 3 | 3.145 | 1.016 | 3556.438 | 1.153 | 5.137 |
| Notes: Dependent Variable: B-IPQ_Concern. Time 1 = 1 day pre-surgery; time 2 = 1 week post-surgery; time 3 = 6 months follow-up-assessment. Adjusted score T0 (baseline) = 6.15. | | | | | | |

**Mixed Model Analysis – Results for Understanding**

| *Model Dimension* | | | | | | |
| --- | --- | --- | --- | --- | --- | --- |
|  | | Number of Levels | Covariance Structure | Number of Parameters | Subject Variables | Number of Subjects |
| Fixed Effects | Intercept | 1 |  | 1 |  |  |
|  | Group | 3 |  | 2 |  |  |
|  | time | 3 |  | 2 |  |  |
|  | Group * time | 9 |  | 4 |  |  |
|  | B-IPQ_Understanding_T0 | 1 |  | 1 |  |  |
|  | Group * B-IPQ_Understanding_T0 | 3 |  | 2 |  |  |
|  | time * B-IPQ_Understanding_T0 | 3 |  | 2 |  |  |
|  | Group * time * B-IPQ_Understanding_T0 | 9 |  | 4 |  |  |
| Random Effects | Intercept | 1 | Variance Components | 1 |  |  |
| Repeated Effects | time | 3 | Compound Symmetry | 2 | id | 108 |
| Total | | 36 |  | 21 |  |  |
| Notes: Dependent Variable: B-IPQ_Understanding. | | | | | | |

| *Type III Tests of Fixed Effects* | | | | |
| --- | --- | --- | --- | --- |
| Source | Numerator df | Denominator df | F | Sig. |
| Intercept | 1 | 102.352 | 196.740 | <.001 |
| Group | 2 | 102.310 | 2.524 | .085 |
| time | 2 | 182.555 | .986 | .375 |
| Group * time | 4 | 182.219 | 1.706 | .150 |
| B-IPQ_Understanding_T0 | 1 | 101.178 | 8.153 | .005 |
| Group * B-IPQ_Understanding_T0 | 2 | 101.361 | 1.970 | .145 |
| time * B-IPQ_Understanding_T0 | 2 | 181.322 | .678 | .509 |
| Group * time * B-IPQ_Understanding_T0 | 4 | 181.222 | 1.337 | .258 |
| Notes: Dependent Variable: B-IPQ_Understanding. | | | | |

| *Estimates of Fixed Effects* | | | | | | | |
| --- | --- | --- | --- | --- | --- | --- | --- |
| Parameter | Estimate | Std. Error | df | t | Sig. | 95% Confidence Interval | |
|  |  |  |  |  |  | Lower Bound | Upper Bound |
| Intercept | 4.799595 | 1.181779 | 240.645 | 4.361 | <.001 | 2.608942 | 6.990246 |
| [Group=1] | .118846 | 1.516775 | 237.764 | .078 | .938 | -2.869187 | 3.106880 |
| [Group=2] | 4.244217 | 1.523806 | 240.608 | 2.785 | .006 | 1.242513 | 7.245921 |
| [Group=3] | 0^a^ | 0 |  |  |  |  |  |
| [time=1] | 2.290299 | 1.252663 | 183.383 | 1.828 | .069 | -.181185 | 4.761782 |
| [time=2] | .859457 | 1.202428 | 176.947 | .715 | .476 | -1.513487 | 3.232402 |
| [time=3] | 0^a^ | 0 |  |  |  |  |  |
| [time=1] * [Group=1] | -.865720 | 1.691690 | 180.527 | -.512 | .609 | -4.203750 | 2.472309 |
| [time=2] * [Group=1] | -.823294 | 1.650191 | 176.641 | -.499 | .618 | -4.079920 | 2.433333 |
| [time=3] * [Group=1] | 0^a^ | 0 |  |  |  |  |  |
| [time=1] * [Group=2] | -4.275948 | 1.762662 | 193.391 | -2.426 | .016 | -7.752457 | -.799440 |
| [time=2] * [Group=2] | -2.814516 | 1.752840 | 192.404 | -1.606 | .110 | -6.271766 | .642733 |
| [time=3] * [Group=2] | 0^a^ | 0 |  |  |  |  |  |
| [time=1] * [Group=3] | 0^a^ | 0 |  |  |  |  |  |
| [time=2] * [Group=3] | 0^a^ | 0 |  |  |  |  |  |
| [time=3] * [Group=3] | 0^a^ | 0 |  |  |  |  |  |
| B-IPQ_Understanding_T0 | .338434 | .141749 | 235.899 | 2.388 | .018 | .059178 | .617690 |
| [Group=1] * B-IPQ_Understanding_T0 | -.037069 | .203134 | 235.338 | -.182 | .855 | -.437263 | .363126 |
| [Group=2] * B-IPQ_Understanding_T0 | -.486531 | .195107 | 240.183 | -2.494 | .013 | -.870870 | -.102192 |
| [Group=3] * B-IPQ_Understanding_T0 | 0^a^ | 0 |  |  |  |  |  |
| [time=1] * B-IPQ_Understanding_T0 | -.175356 | .158528 | 180.284 | -1.106 | .270 | -.488166 | .137454 |
| [time=2] * B-IPQ_Understanding_T0 | -.071995 | .153668 | 175.477 | -.469 | .640 | -.375271 | .231281 |
| [time=3] * B-IPQ_Understanding_T0 | 0^a^ | 0 |  |  |  |  |  |
| [time=1] * [Group=1] * B-IPQ_Understanding_T0 | -.018052 | .224774 | 178.321 | -.080 | .936 | -.461611 | .425508 |
| [time=2] * [Group=1] * B-IPQ_Understanding_T0 | .102533 | .222120 | 176.429 | .462 | .645 | -.335822 | .540887 |
| [time=3] * [Group=1] * B-IPQ_Understanding_T0 | 0^a^ | 0 |  |  |  |  |  |
| [time=1] * [Group=2] * B-IPQ_Understanding_T0 | .419346 | .222482 | 191.502 | 1.885 | .061 | -.019484 | .858176 |
| [time=2] * [Group=2] * B-IPQ_Understanding_T0 | .292108 | .223233 | 190.325 | 1.309 | .192 | -.148220 | .732436 |
| [time=3] * [Group=2] * B-IPQ_Understanding_T0 | 0^a^ | 0 |  |  |  |  |  |
| [time=1] * [Group=3] * B-IPQ_Understanding_T0 | 0^a^ | 0 |  |  |  |  |  |
| [time=2] * [Group=3] * B-IPQ_Understanding_T0 | 0^a^ | 0 |  |  |  |  |  |
| [time=3] * [Group=3] * B-IPQ_Understanding_T0 | 0^a^ | 0 |  |  |  |  |  |
| Notes: Group 1 = SMC, Group 2 = SUPPORT, Group 3 = EXPECT. Time 1 = 1 day pre-surgery; time 2 = 1 week post-surgery; time 3 = 6 months follow-up-assessment. Dependent Variable: B-IPQ_Understanding. a. This parameter is set to zero because it is redundant. | | | | | | | |

| *Estimates of Covariance Parameters* | | | |  |  |  |  |
| --- | --- | --- | --- | --- | --- | --- | --- |
| Parameter | | Estimate | Std. Error | Wald Z | Sig. | 95% Confidence Interval | |
|  |  |  |  |  |  | Lower Bound | Upper Bound |
| Repeated Measures | CS diagonal offset | 2.698679 | .293688 | 9.189 | <.001 | 2.180308 | 3.340294 |
|  | CS covariance | 1.782415 | .417098 | 4.273 | <.001 | .964919 | 2.599911 |
| Intercept | Variance | ^.^159883 | 4842165,087 | .000 | 1.000 | .000000 |  |
| Notes: Dependent Variable: B-IPQ_Understanding. | | | |  |  |  |  |

| *Estimates for Means (group)* | | | | | |
| --- | --- | --- | --- | --- | --- |
| group | Mean | Std. Error | df | 95% Confidence Interval | |
|  |  |  |  | Lower Bound | Upper Bound |
| SMC | 7.160 | .275 | 95.932 | 6.614 | 7.705 |
| SUPPORT | 7.777 | .294 | 103.054 | 7.193 | 8.361 |
| EXPECT | 7.667 | .278 | 96.154 | 7.114 | 8.219 |
| Notes: Dependent Variable: B-IPQ_Understanding. Adjusted score T0 (baseline) = 7.10. | | | | | |

| *Estimates for Means (time)* | | | | | |
| --- | --- | --- | --- | --- | --- |
| time | Mean | Std. Error | df | 95% Confidence Interval | |
|  |  |  |  | Lower Bound | Upper Bound |
| 1 | 7.699 | .215 | 2226.442 | 7.275 | 8.123 |
| 2 | 7.487 | .213 | 223.040 | 7.067 | 7.908 |
| 3 | 7.418 | .218 | 229.911 | 6.989 | 7.846 |
| Notes: Dependent Variable: B-IPQ_Understanding. Time 1 = 1 day pre-surgery; time 2 = 1 week post-surgery; time 3 = 6 months follow-up-assessment. Adjusted score T0 (baseline) = 7.10. | | | | | |

| *Estimates for level of B-IPQ Understanding (average (mean))* | | | | | | |
| --- | --- | --- | --- | --- | --- | --- |
| group | time | Mean | Std. Error | df | 95% Confidence Interval | |
|  |  |  |  |  | Lower Bound | Upper Bound |
| SMC | 1 | 7.109 | .355 | 217.609 | 6.409 | 7.810 |
|  | 2 | 7.311 | .358 | 220.259 | 6.605 | 8.017 |
|  | 3 | 7.058 | .369 | 229.838 | 6.331 | 7.786 |
| SUPPORT | 1 | 7.739 | .387 | 229.201 | 6.977 | 8.501 |
|  | 2 | 7.600 | .396 | 234.104 | 6.820 | 8.380 |
|  | 3 | 7.992 | .393 | 234.036 | 7.218 | 8.767 |
| EXPECT | 1 | 8.248 | .375 | 231.246 | 7.508 | 8.987 |
|  | 2 | 7.551 | .353 | 211.503 | 6.854 | 8.247 |
|  | 3 | 7.202 | .368 | 225.170 | 6.477 | 7.927 |
| Notes: Dependent Variable: B-IPQ_Understanding. Time 1 = 1 day pre-surgery; time 2 = 1 week post-surgery; time 3 = 6 months follow-up-assessment. Adjusted score T0 (baseline) = 7.10. | | | | | | |

**Mixed Model Analysis – Results for Emotional response**

| *Model Dimension* | | | | | | | | | | | |
| --- | --- | --- | --- | --- | --- | --- | --- | --- | --- | --- | --- |
|  | | | Number of Levels | | Covariance Structure | | Number of Parameters | | Subject Variables | | Number of Subjects |
| Fixed Effects | Intercept | | 1 | |  | | 1 | |  | |  |
|  | Group | | 3 | |  | | 2 | |  | |  |
|  | time | | 3 | |  | | 2 | |  | |  |
|  | Group * time | | 9 | |  | | 4 | |  | |  |
|  | B-IPQ_Emotional response_T0 | | 1 | |  | | 1 | |  | |  |
|  | Group * B-IPQ_Emotional response_T0 | | 3 | |  | | 2 | |  | |  |
|  | time * B-IPQ_Emotional response_T0 | | 3 | |  | | 2 | |  | |  |
|  | Group * time * B-IPQ_Emotional response_T0 | | 9 | |  | | 4 | |  | |  |
| Random Effects | Intercept | | 1 | | Variance Components | | 1 | |  | |  |
| Repeated Effects | time | | 3 | | Heterogeneous First-Order Autoregressive | | 4 | | id | | 111 |
| Total | | | 36 | |  | | 23 | |  | |  |
| Notes: Dependent Variable: B-IPQ_Emotional response. | | | | | | | | | | | |
| *Type III Tests of Fixed Effects* | | | | | | | | | |  |  |
| Source | | Numerator df | | Denominator df | | F | | Sig. | |  |  |
| Intercept | | 1 | | .000 | | 2.376 | | 1.000 | |  |  |
| Group | | 2 | | 116.655 | | .841 | | .434 | |  |  |
| time | | 2 | | 164.181 | | 4.709 | | .010 | |  |  |
| Group * time | | 4 | | 165.681 | | 1.149 | | .335 | |  |  |
| B-IPQ_Emotional response_T0 | | 1 | | 117.405 | | 61.801 | | .000 | |  |  |
| Group * B-IPQ_Emotional response_T0 | | 2 | | 116.965 | | .522 | | .595 | |  |  |
| time * B-IPQ_Emotional response_T0 | | 2 | | 161.169 | | 3.721 | | .026 | |  |  |
| Group * time * B-IPQ_Emotional response_T0 | | 4 | | 162.035 | | .845 | | .498 | |  |  |
| Notes: Dependent Variable: B-IPQ_Emotional response. | | | | | | | | | |  |  |

| *Estimates of Fixed Effects* | | | | | | | |
| --- | --- | --- | --- | --- | --- | --- | --- |
| Parameter | Estimate | Std. Error | df | t | Sig. | 95% Confidence Interval | |
|  |  |  |  |  |  | Lower Bound | Upper Bound |
| Intercept | .270648 | 1.328937 | .000 | .204 | 1.000 | -4.512582 | 5.053877 |
| [Group=1] | 1.792280 | 1.038470 | 93.967 | 1.726 | .088 | -.269635 | 3.854196 |
| [Group=2] | .362117 | 1.014093 | 93.919 | .357 | .722 | -1.651412 | 2.375646 |
| [Group=3] | 0^a^ | 0 |  |  |  |  |  |
| [time=1] | 2.104861 | .966138 | 189.999 | 2.179 | .031 | .199127 | 4.010594 |
| [time=2] | 2.098651 | .934915 | 142.197 | 2.245 | .026 | .250524 | 3.946779 |
| [time=3] | 0^a^ | 0 |  |  |  |  |  |
| [time=1] * [Group=1] | -2.838642 | 1.412478 | 189.637 | -2.010 | .046 | -5.624829 | -.052456 |
| [time=2] * [Group=1] | -.562041 | 1.445577 | 145.207 | -.389 | .698 | -3.419131 | 2.295048 |
| [time=3] * [Group=1] | 0^a^ | 0 |  |  |  |  |  |
| [time=1] * [Group=2] | -1.519242 | 1.371875 | 189.689 | -1.107 | .270 | -4.225333 | 1.186848 |
| [time=2] * [Group=2] | -.409195 | 1.367121 | 141.643 | -.299 | .765 | -3.111794 | 2.293404 |
| [time=3] * [Group=2] | 0^a^ | 0 |  |  |  |  |  |
| [time=1] * [Group=3] | 0^a^ | 0 |  |  |  |  |  |
| [time=2] * [Group=3] | 0^a^ | 0 |  |  |  |  |  |
| [time=3] * [Group=3] | 0^a^ | 0 |  |  |  |  |  |
| B-IPQ_Emotional response_T0 | .423571 | .120324 | 93.683 | 3.520 | .001 | .184654 | .662488 |
| [Group=1] * B-IPQ_Emotional response_T0 | -.253631 | .198449 | 94.063 | -1.278 | .204 | -.647653 | .140391 |
| [Group=2] * B-IPQ_Emotional response_T0 | -.014173 | .178967 | 93.949 | -.079 | .937 | -.369519 | .341174 |
| [Group=3] * B-IPQ_Emotional response_T0 | 0^a^ | 0 |  |  |  |  |  |
| [time=1] * B-IPQ_Emotional response_T0 | .074435 | .167743 | 190.327 | .444 | .658 | -.256440 | .405309 |
| [time=2] * B-IPQ_Emotional response_T0 | -.022885 | .162359 | 139.190 | -.141 | .888 | -.343894 | .298124 |
| [time=3] * B-IPQ_Emotional response_T0 | 0^a^ | 0 |  |  |  |  |  |
| [time=1] * [Group=1] * B-IPQ_Emotional response_T0 | .432922 | .267316 | 189.200 | 1.620 | .107 | -.094381 | .960226 |
| [time=2] * [Group=1] * B-IPQ_Emotional response_T0 | .060951 | .272174 | 146.535 | .224 | .823 | -.476942 | .598845 |
| [time=3] * [Group=1] * B-IPQ_Emotional response_T0 | 0^a^ | 0 |  |  |  |  |  |
| [time=1] * [Group=2] * B-IPQ_Emotional response_T0 | .124971 | .244898 | 190.089 | .510 | .610 | -.358097 | .608038 |
| [time=2] * [Group=2] * B-IPQ_Emotional response_T0 | .078368 | .239356 | 141.971 | .327 | .744 | -.394794 | .551530 |
| [time=3] * [Group=2] * B-IPQ_Emotional response_T0 | 0^a^ | 0 |  |  |  |  |  |
| [time=1] * [Group=3] * B-IPQ_Emotional response_T0 | 0^a^ | 0 |  |  |  |  |  |
| [time=2] * [Group=3] * B-IPQ_Emotional response_T0 | 0^a^ | 0 |  |  |  |  |  |
| [time=3] * [Group=3] * B-IPQ_Emotional response_T0 | 0^a^ | 0 |  |  |  |  |  |
| Notes: Group 1 = SMC, Group 2 = SUPPORT, Group 3 = EXPECT. Time 1 = 1 day pre-surgery; time 2 = 1 week post-surgery; time 3 = 6 months follow-up-assessment. Dependent Variable: B-IPQ_Emotional response. a. This parameter is set to zero because it is redundant. | | | | | | | |

| *Estimates of Covariance Parameters* | | | | | | | |
| --- | --- | --- | --- | --- | --- | --- | --- |
| Parameter | | Estimate | Std. Error | Wald Z | Sig. | 95% Confidence Interval | |
|  |  |  |  |  |  | Lower Bound | Upper Bound |
| Repeated Measures | Var: [time = 1] | 5.244293 | .741297 | 7.074 | <.001 | 3.975272 | 6.918420 |
|  | Var: [time = 2] | 7.378107 | 1.056508 | 6.983 | <.001 | 5.572591 | 9.768609 |
|  | Var: [time = 3] | 5.092804 | .745867 | 6.828 | <.001 | 3.822030 | 6.786093 |
|  | ARH1, rho | .232928 | .070644 | 3.297 | .001 | .090630 | .365905 |
| Intercept | Variance | 1.273532 | 94906265.62 | .000 | 1.000 | .000000 |  |
| Notes: Dependent Variable: B-IPQ_Emotional response. Time 1 = 1 day pre-surgery; time 2 = 1 week post-surgery; time 3 = 6 months follow-up-assessment. | | | | | | | |

| *Estimates for Means (group)* | | | | | |
| --- | --- | --- | --- | --- | --- |
| group | Mean | Std. Error | df | 95% Confidence Interval | |
|  |  |  |  | Lower Bound | Upper Bound |
| SMC | 3.933 | 1.160 | .000 | -3.873 | 11.739 |
| SUPPORT | 3.643 | 1.165 | .000 | -1.287 | 8.574 |
| EXPECT | 3.680 | 1.162 | .000 | -2.922 | 1.281 |
| Notes: Dependent Variable: B-IPQ_Emotional response. Adjusted score T0 (baseline) = 4.56. | | | | | |

| *Estimates for Means (time)* | | | | | |
| --- | --- | --- | --- | --- | --- |
| time | Mean | Std. Error | df | 95% Confidence Interval | |
|  |  |  |  | Lower Bound | Upper Bound |
| 1 | 4.350 | 1.151 | .000 | -4.034 | 12.734 |
| 2 | 4.394 | 1.160 | .000 | 1.269 | 7.519 |
| 3 | 2.512 | 1.151 | .000 | -5.294 | 1.318 |
| Notes: Dependent Variable: B-IPQ_Emotional response. Time 1 = 1 day pre-surgery; time 2 = 1 week post-surgery; time 3 = 6 months follow-up-assessment. Adjusted score T0 (baseline) = 4.56. | | | | | |

| *Estimates for level of B-IPQ Emotional response (average (mean))* | | | | | | |
| --- | --- | --- | --- | --- | --- | --- |
| group | time | Mean | Std. Error | df | 95% Confidence Interval | |
|  |  |  |  |  | Lower Bound | Upper Bound |
| SMC | 1 | 4.415 | 1.190 | .000 | -2.517 | 11.346 |
|  | 2 | 4.547 | 1.215 | .000 | -.185 | 9.279 |
|  | 3 | 2.837 | 1.190 | .000 | -3.803 | 9.477 |
| SUPPORT | 1 | 3.992 | 1.197 | .000 | -.205 | 8.188 |
|  | 2 | 4.440 | 1.229 | .000 | .562 | 8.318 |
|  | 3 | 2.498 | 1.201 | .000 | -3.768 | 8.764 |
| EXPECT | 1 | 4.644 | 1.195 | .000 | -.212 | 9.500 |
|  | 2 | 4.195 | 1.216 | .000 | -.390 | 8.779 |
|  | 3 | 2.200 | 1.195 | .000 | -2.530 | 6.931 |
| Notes: Dependent Variable: B-IPQ_Emotional response. Time 1 = 1 day pre-surgery; time 2 = 1 week post-surgery; time 3 = 6 months follow-up-assessment. Adjusted score T0 (baseline) = 4.56. | | | | | | |
